# Identification of Prey Captures in Australian Fur Seals (*Arctocephalus pusillus doriferus*) Using Head-Mounted Accelerometers: Field Validation with Animal-Borne Video Cameras

**DOI:** 10.1371/journal.pone.0128789

**Published:** 2015-06-24

**Authors:** Beth L. Volpov, Andrew J. Hoskins, Brian C. Battaile, Morgane Viviant, Kathryn E. Wheatley, Greg Marshall, Kyler Abernathy, John P. Y. Arnould

**Affiliations:** 1 Deakin University, School of Life and Environmental Sciences, Burwood, Victoria, Australia; 2 University of British Columbia, Marine Mammal Research Unit, Fisheries Centre, Vancouver, BC, Canada; 3 Centre d’Etudes Biologiques de Chize', Centre National de la Recherche Scientifique, Villiers en Bois, France; 4 National Geographic, Remote Imaging Department, Washington, DC, United States of America; University of St Andrews, UNITED KINGDOM

## Abstract

This study investigated prey captures in free-ranging adult female Australian fur seals (*Arctocephalus pusillus doriferus*) using head-mounted 3-axis accelerometers and animal-borne video cameras. Acceleration data was used to identify individual attempted prey captures (APC), and video data were used to independently verify APC and prey types. Results demonstrated that head-mounted accelerometers could detect individual APC but were unable to distinguish among prey types (fish, cephalopod, stingray) or between successful captures and unsuccessful capture attempts. Mean detection rate (true positive rate) on individual animals in the testing subset ranged from 67-100%, and mean detection on the testing subset averaged across 4 animals ranged from 82-97%. Mean False positive (FP) rate ranged from 15-67% individually in the testing subset, and 26-59% averaged across 4 animals. Surge and sway had significantly greater detection rates, but also conversely greater FP rates compared to heave. Video data also indicated that some head movements recorded by the accelerometers were unrelated to APC and that a peak in acceleration variance did not always equate to an individual prey item. The results of the present study indicate that head-mounted accelerometers provide a complementary tool for investigating foraging behaviour in pinnipeds, but that detection and FP correction factors need to be applied for reliable field application.

## Introduction

Foraging success is one of the main determinants of individual survival (e.g. [1]). Factors that influence foraging success can impact reproductive success, population growth, and ultimately the survival of a species, as documented in both marine mammals [1–5] and diving birds [6–8]. Direct observation of foraging behaviour is not possible for most marine mammals and, consequently, alternate methods using animal-borne data loggers have attempted to provide this type of information.

Dive characteristics obtained from time-depth-recorders (TDR) have been used over the last two decades to obtain information on diving behaviour as an indirect measure of foraging success (e.g. [9]), typically by classifying dives into “foraging” and “non-foraging” events [5,10–14]. However, there is uncertainty as to whether data from TDRs alone can differentiate between successful foraging, searching dives, unsuccessful attempted prey captures, or prey quantity or species [5,10–14]. Researchers have also used changes in stomach temperature to estimate prey ingestion in marine mammals [15], but tests of this method’s accuracy remain inconclusive [16–19]. Stomach temperature dataloggers are also limited by their size and typically short retention times, which varies among and within species [11,20]. Although stomach sensor dataloggers can be used to complement inferred foraging from dive depth profiles, these dataloggers are not able to quantify feeding events or distinguish prey type [16,17,19].

Animal-borne video cameras have been used for direct observation of foraging behaviour in several free-ranging pinnipeds including Weddell seals [21,22], harbor seals [23], Antarctic fur seals [24], and Hawaiian monk seals [25]. The size of the camera limits battery and memory capacity and, therefore, these instruments can rarely record continuously and dives must be subsampled. The high expense of the video cameras also often limits the number of animals that video cameras can be deployed on. Furthermore, manual analysis of video or still photo data can be labor intensive and time consuming. However, animal-borne video cameras are valuable as a tool to validate the ability of other types of data loggers to detect foraging success eliminating the necessity of cameras for future deployments [26].

Head-mounted accelerometers can identify specific patterns in head and jaw movements during prey capture and prey handling events in diving pinnipeds and penguins [27–31]. Recent advances in datalogger technology have greatly reduced the size of dataloggers making it possible to glue dataloggers on animal’s heads with less drag and behavioural impacts [23,32,33]. However, adequate, species-specific validation is required under realistic conditions to develop the detection algorithms and test the effectiveness of the method. Previous validations have been undertaken on captive Steller sea lions in aquaria [28,31], but these did not mimic realistic prey intake patterns, nor did they investigate the role of multiple prey types. More importantly, as they only examined the ability to detect known prey captures, the results cannot be used to calculate meaningful measures of detection success.

The present study examined the efficacy of head-mounted accelerometers to accurately detect attempted prey captures (APC) and identify prey type in free-ranging Australian fur seals (*Arctocephalus pusillus doriferus*) using animal-borne video data loggers to provide visual validation of prey capture success/misses and prey species identification. The overall research objective was to quantify APC using head mounted 3-axis accelerometers, and determine the error (FP, false positive rate) of accelerometers relative to known foraging behavior determined from animal-borne video. Specifically, this study determined if head-mounted accelerometers could distinguish dives with APC present dives without prey present, successful from unsuccessful APC, and the possible effect of prey type. We hypothesized that the successful APC would have longer durations, greater integral area and more peaks per APC due to handling of the prey. We also hypothesized that larger prey items, such as cephalopods, would increase the same metrics compared to smaller prey due to increase prey handling. Finally, this study strived to present a transparent and repeatable method of quantifying APC that was relevant to field application.

## Materials and Methods

### Ethics statement

All work was conducted with approval of the Deakin University Animal Ethics Committee (A14/2011) and under the Department of Sustainability and Environment (Victoria, Australia) wildlife Research Permits (10005484). Kanowna Island is within the Wilsons Promontory Marine National Park and was accessed under permit from Parks Victoria.

**Fig 1:**
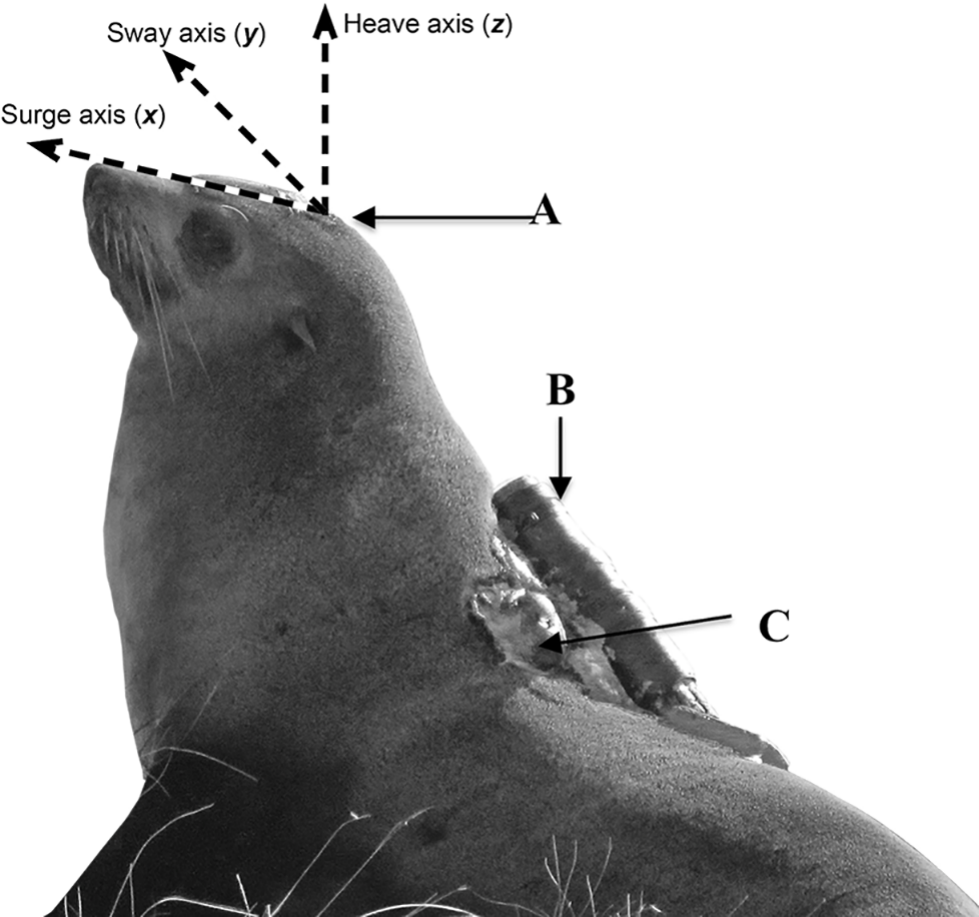
Photo of dataloggers deployed on Australian fur seals. Seals were instrumented with (A) accelerometer measuring surge (anterior-posterior), sway (lateral), and heave (dorsal-ventral) (B) National Geographic Crittercam measuring video, and (C) time-depth-recorder.

Data were collected on adult females provisioning pups from May to July (fall and winter) in 2011–2012 at Kanowna Island, northern Bass Strait (39° 9.1’S, 146° 18.5 ‘E) in southeastern Australia. Animal capture and anaesthesia methods have previously been detailed (same procedures without restraint board, [34]). Once captured, individuals were instrumented with a 3-axis accelerometer that measured surge (x, anterior-posterior), sway (y, lateral) and heave (z,dorsal-ventral) at 20 Hz (± 3g, G6A, 40 X 28 X 16.3 mm, Cefas Technology Limited, Suffolk, United Kingdom), a GPS datalogger (5 min sample rate, Fastlock 2 GPS datalogger, 69 X 28 X 21 mm, Sirtrack, NZ), a time-depth-recorder (1 Hz, TDR, MK9-TDR, 68 X 17 X 17 mm, Wildlife Computers, Redmond, WA, USA) and an animal-borne video camera (Crittercam, 25 cm length X 5.7 cm diameter, National Geographic Society, Fig 1). Maximum recording time for the accelerometers was 8 days. The Crittercams were programmed to record video when submerged > 40 m on a duty cycle of 1 hour on: 3 hour off. TDR, head accelerometers and video data were successfully recovered on 8 animals with full sets of overlapping useable data available for 4 animals (Table 1).

**Table 1:** Summary of useable dives.

			Random Training Subset			Random Testing Subset	
Animal	Mass (kg)	Useable dives	Prey Present	Prey Absent	Proportion of dives in Training Subset	Prey Present	Prey Absent
W1855	50.5	48	16	8	50%	15	9
W1859	54.5	32	14	3	53%	14	1
W1873	88.0	77	29	8	48%	26	14
W1881	88.5	36	15	3	50%	17	1
Total		193	74	22		72	25

Total useable dives (n = 193) with overlapping depth, video, and 3-axis accelerometer data per Australian fur seal. For cross-validation, each dive was randomly assigned to the training or testing subset (approximately 50% each). Dives with prey visible in video were classified as “prey present”, and dives with no prey visible on video were classified as “prey absent”. Prey chases without capture attempts on video were classified as “prey absent”.

### Data processing

The raw time and depth data were zero-offset corrected and processed using the statistical software program R 3.0.1 [35]. Maximum dive depth, and dive descent, ascent, and bottom durations were identified using changes in depth slopes (minimum dive depth threshold 15 m). Australian fur seals are predominately benthic foragers, and the maximum depth of Bass Strait on the shallow continental shelf is < 100 m [9,36]. Preliminary analysis showed that maximum dive depths of dives study ranged from 61–86 m. Therefore, the 40 m threshold for the video cameras allowed capture of all dives of interest. Depth and time data from the TDR were linearly interpolated from 1 Hz to 20 Hz to match the 20 Hz sampling frequency of the accelerometer.

**Fig 2:**
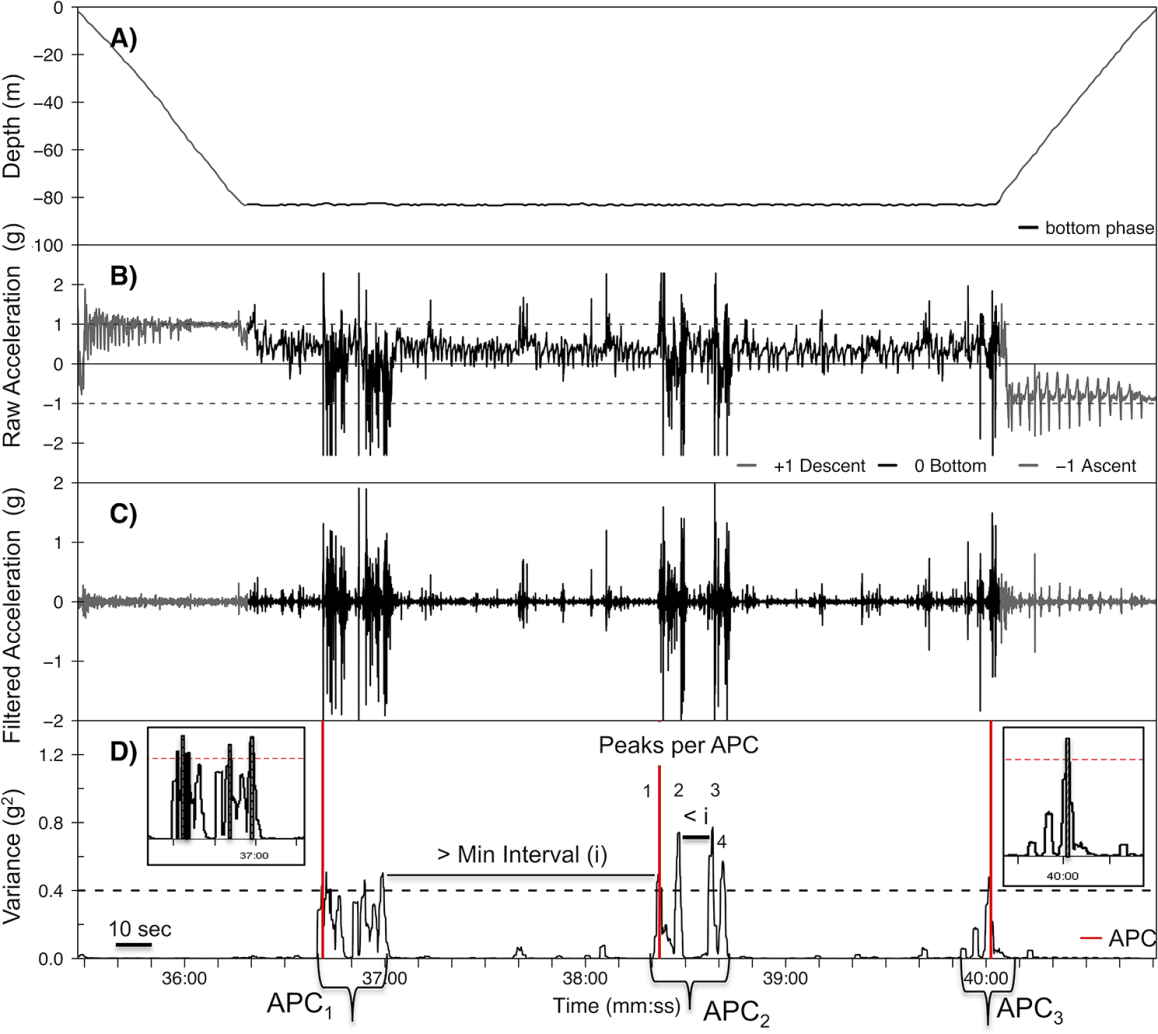
Diagram of accelerometer data processing method. Example of accelerometer identification of attempted prey captures (APC) with variance of acceleration on the surge axis. Dive depth (A) was synchronized with raw acceleration (g, B) as evident by the bottom phase of the dive matching on A and B. Head movements were isolated from body movements with a 3 Hz high-pass filter (C) and variance of acceleration (g^2^) was calculated for each individual dive (excluding surface ≥ 2 m). Peaks in variance of acceleration above a variance threshold (——) and within a minimum time interval (i) were used to estimate APC (APC, D). Consecutive peaks greater than the minimum interval apart were counted as separate APC, and events less than the interval were counted as single APC. Duration of individual APC (indicated by brackets), integral area under the peak of variance (g^2^, inset, cumulative integral of individual peaks in APC group), time of the first peak in each APC (**−**), and number of peaks per APC were also calculated.

Additional data processing was required to remove artifacts in clock alignment among the TDR, video camera, and accelerometer. The time stamps of the TDR, accelerometer, and video data were aligned visually to within ± 1 s using the raw surge acceleration with Eonfusion software (Eonfusion, v.1.2, www.myriax.com) and customized functions in R for each individual dive. Dives that did not have complete video coverage and dives not aligned within ± 1 s due to extensive clock drifts or malfunctions were excluded. Head movements were isolated from body movements and swimming movements using a 3 Hz3 Hz 3 Hz 3 Hz 3 Hz 3 Hz 3 Hz high-pass filter on each axis which also accentuated the peaks in variance (signal package in R, Fig 2C, [37]).

Identification of APC from the accelerometry data was performed using a custom-written function comprised of several custom routines in R modified from Viviant et al. (referred to as Function 1, available on request from authors, [31]). Function 1 calculated the variance of acceleration signal along a moving 1.5 s window of each dive to identify and count the peaks in head movement indicative of APCs (Fig 2D, S1 Text). This Function performed 6 general tasks: i) calculated the variance of acceleration, ii) defined the peaks in variance of acceleration using the variance threshold and minimum time between 2 successive peaks, iii) calculated the cumulative integral area under the peak of variance for each APC (g^2^), iv) estimated the duration of the APC, v) counted the number of peaks within an APC, and vi) counted the number of total APC per dive. This data was determined for each of the three axes (surge, sway, and heave) individually on all dives for each animal. In addition, the values chosen for variance thresholds and minimum intervals were selected by a model optimization procedure (see *Selection of variance threshold and minimum interval*).

### Categorization of APC on video

**Fig 3:**
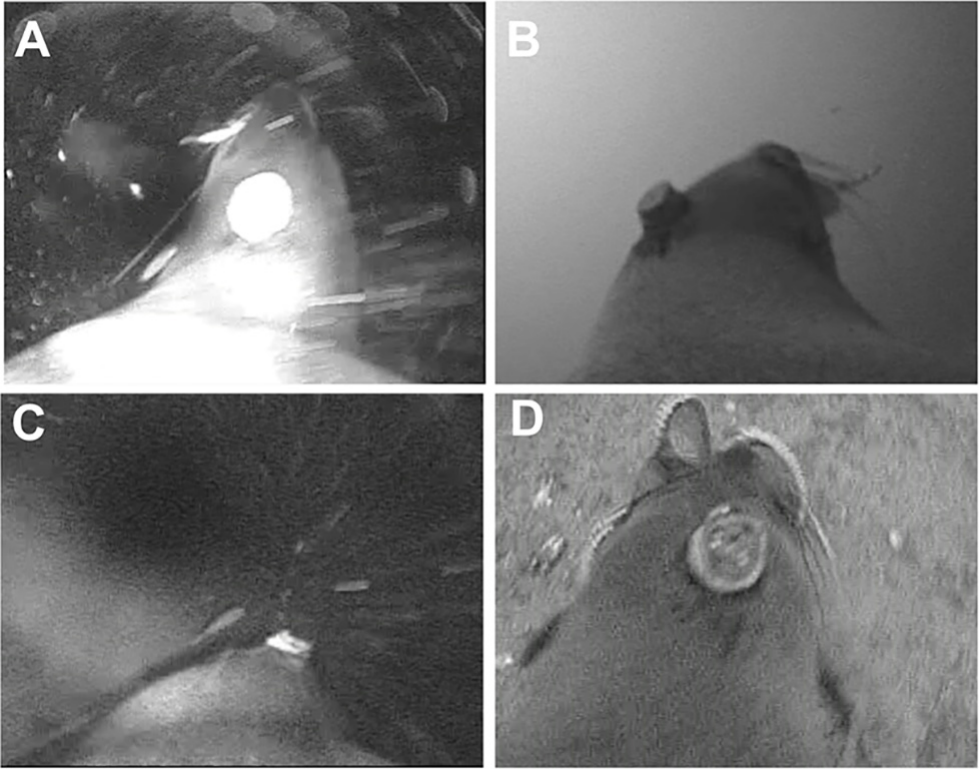
Still captures from the Crittercam video on Australian fur seals. Examples of prey and potential outcomes included a successful fish capture (A), successful stingray capture (B), unsuccessful fish capture (fish left of seal’s head, C), and successful octopus capture. A 3-dimensional accelerometer mounted on the fur seal’s head was used to measure acceleration during foraging.

Video identification was performed independent of acceleration analysis as part of a concurrent project [38]. Prey type (fish, stingray, octopus and squid combined, or unknown) and location at which the prey was consumed (benthic or during ascent) were recorded (Fig 3, [38]). APC occurred when seals attempted to capture 1 potential prey item, and included both captures and capture attempts. Video clips that contained an APC were identified and categorized into the following behavioural categories: chase and missed capture attempt, chase and capture with handling, chase and capture without handling, capture without chase. Chases followed by an unsuccessful capture attempt (head lunge towards prey, but no prey visible in mouth or no jaw movements) were identified as “chase and missed capture attempt”. Chases that were followed by resuming of search swimming (without capture attempt lunge towards prey) were excluded. Potential APC were classified as either unsuccessful (“chase and missed capture attempt”) or successful (all other combinations of chase, capture, handling). Chases were considered unsuccessful unless there was visual confirmation of the prey being captured (jaw movements during handling and/or prey visible in mouth). The depth and acceleration datasets were synchronized at 20 Hz and subset to have the same start time. The APC identified by the accelerometer were matched to events identified on video data using custom matching functions in R based on a common time vector synced to both datasets. Accuracy of matching functions were verified by plotting Fig 2 for each dive (See S1 Text for details).

Each individual APC was classified as true positive (TP), true negative (TN), false positive (FP), and false negative (FN) relative to the true values ascertained via the video to create a contingency table (i.e. confusion matrix, Table 2, [39]). A match between the accelerometer and video occurred when the prey capture time from the accelerometer occurred between the true start of the chase and the end of handling for successful events on the video (true positive) or between the start and end of chase if unsuccessful (also true positive). If the APC on the accelerometer fell outside the video start and end times of the specific event on video for that dive, then it was counted as a FP. FP also occurred when the accelerometer matched 2 or more separate APC to the same capture event on video. For example, if the accelerometer incorrectly identified 2 APC that were associated with only 1 APC on video, one event was categorized as TP, and the other matches (the extra or “multiple matches”) were categorized at FP. These were counted as a FP because the accelerometer incorrectly counted more than 1 APC when there was actually 1 APC on video, thus overestimating APC. Multiple matches occurred moderately relative to the total APC per axis (surge = 34%, sway = 42%, heave = 23% APC on testing subset of dives relative to total APC in Table 3, generic parameters). Accounting for multiple matches as FP means that the FP rate estimates are conservative. Post hoc analysis suggested that multiple matches occurred more frequently for APC with longer durations or for cephalopods and rays. If the accelerometer missed an event that occurred on video, it was counted as a FN. TN occurred when both the accelerometer and the video indicated no APC for an entire dive (i.e. no APC on video, but potentially prey chases without capture attempts). Only 1 TN could be possibly categorized for a single dive and TN vales were therefore highly affected by sample size of prey absent dives. Identifying the absence of animals foraging in the wild is not straightforward, and there are multiple interpretations of how to define the duration and behavioural criteria for a TN. All types of APC (TN, FP, FN, TP) were used for model optimization on the training subset, but due to the issues with defining TN, raw TN values that may be misapplied out of context were not reported. This study also did not report “accuracy” or “specificity” which rely on this parameter (as defined in, [31]).The matching function was repeated for each APC within each dive to determine which specific APC the accelerometer correctly identified, missed, or incorrectly classified relative to the correct classification from video (S1 Text for details). This was repeated on all 3 axes for each animal.

**Table 2:** Categorization of attempted prey captures (APC).

	Video counted prey	Accelerometer counted prey	Description
	(Truth)	(Estimate)	
True Positive (TP)	yes	yes	Accelerometer and video both identified APC
True Negative (TN)	no	no	APC not present on video or accelerometer
False Positive (FP)	no	yes	Accelerometer identified a prey that was not present on video
False Negative (FN)	yes	no	Accelerometer missed a true prey that was present on video

Each APC was classified as true positive (TP), true negative (TN), false positive (FP), and false negative (FN) relative to the actual values on the animal-borne video.

**Table 3:** Summary of accelerometer error metrics relative to video data calculated on Australian fur seals.

	Function 1	Averaged over all animals (%)						Total
Acceleration	Parameters	Detection (± SD)		FP rate (± SD)		Precision (± SD)		APC
X Surge	Generic	96.3	(3.2)	48.1	(15.3)	51.9	(15.3)	327
	Animal-specific	93.9	(3.0)	42.1	(15.0)	57.9	(15.0)	306
Y Sway	Generic	96.8	(3.2)	58.6	(5.8)	41.4	(5.8)	402
	Animal-specific	91.3	(7.1)	45.1	(18.5)	54.9	(18.5)	361
Z Heave	Generic	82.3	(10.8)	32.8	(13.9)	67.2	(13.9)	262
	Animal-specific	81.7	(9.4)	26.1	(16.1)	73.9	(16.1)	252

**Fig 4:**
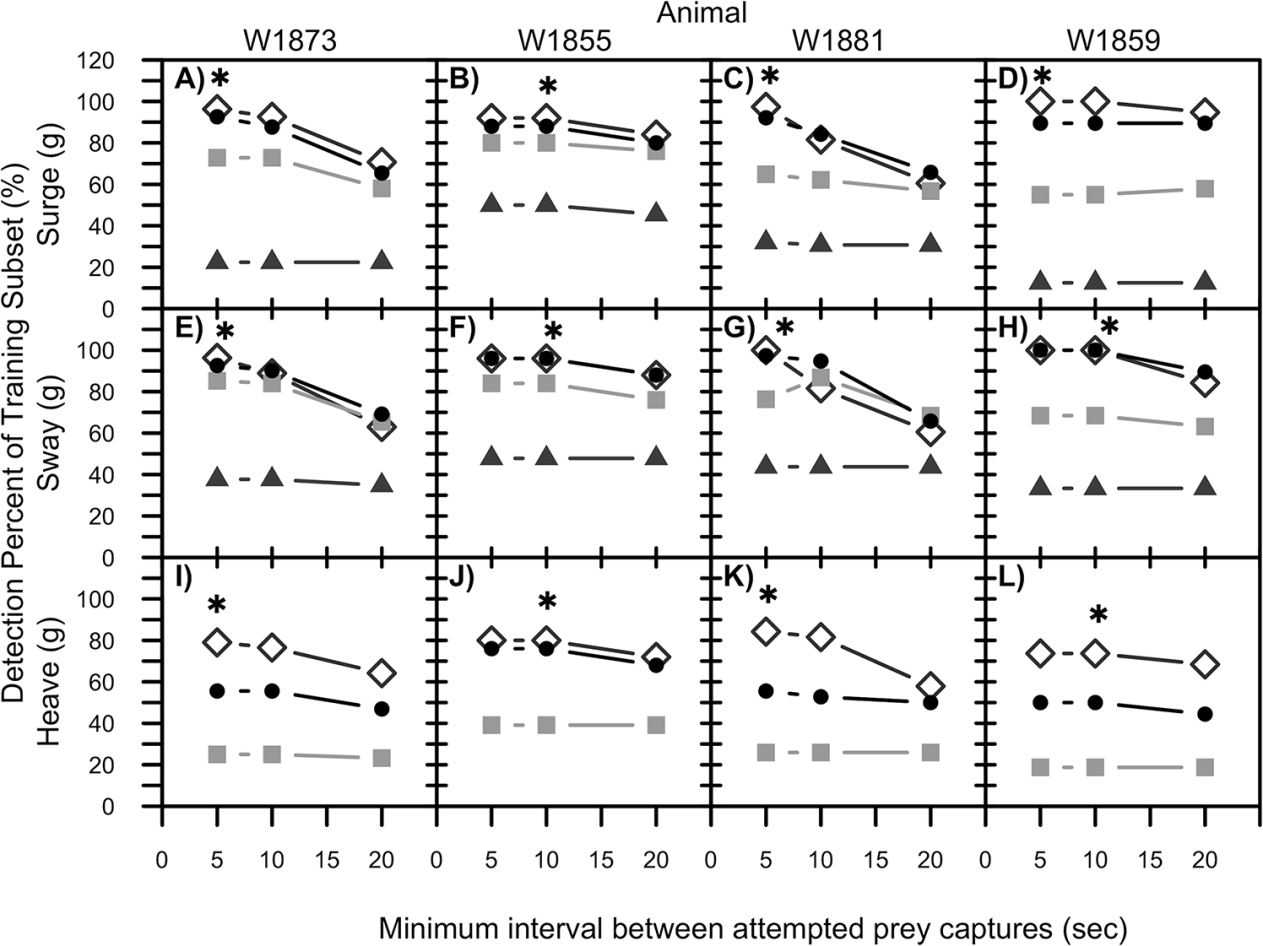
Selection of model parameters on training subset. Detection rate of Function 1 used to identify attempted prey captures (APC) during optimization on the training subset of data. Parameters with the greatest detection rate (*) on the training subset were used to test Function 1 on the other approximately 50% of the dives in the testing subset (termed animal-specific parameters). Each data point represents the mean detection over all APC per animal within the training subset for each parameter combination. Variance thresholds tested included 0.1 (open diamond), 0.2 (black circle), 0.4 (grey square), 0.8 (grey triangle; sway and heave only). Variance thresholds tested for the heave axis were lower than the surge and sway because heave acceleration had lower amplitude peaks.

Error metrics included detection, false positive rate (FP rate), and precision as defined in the abbreviations. Animal-specific parameters were the parameters of minimum interval and variance threshold that yielded the greatest detection per animal per acceleration axis (Fig 4). Generic parameters (same for all animal) were set at 0.1variance threshold with 5 second minimum interval for all animals and acceleration axis. Data are from the Random Testing subset (n = 97 dives).

### Calculation of acceleration error metrics

The total counts of TP, TN, FP, and FN were used to calculate error metrics of detection, FP rate, and precision across all prey captures for each animal. The error metrics for each axis were averaged over all of the dives and individual APC for each animal (each data point equaled the mean per animal). All error metrics described below included both successful captures and unsuccessful capture attempts combined unless noted differently. Detection rate (true positive rate) was calculated as the proportion of true positives that were correctly identified as true positives out of all potential events with feeding (TP/(TP+FN), i.e. sensitivity or recall rate). FP rate, was calculated as the proportion of true positives that “misclassified” or incorrectly classified as a TP when there was no prey on video (FP/(FP+TP)). Precision was calculated as the proportion of actual positives in the number of APC that were classified as either true positive or false negative (TP/TP+FP)). Precision and false rate are inverses of each other (FP rate + precision = 100%). The current study focused on FP rate because it was more intuitive to understand. Precision values are provided in tables and where appropriate with literature comparisons.

### Selection of variance threshold and minimum interval

The two parameters of Function 1 that were changed during optimization were variance threshold and minimum duration between two consecutive peaks above a threshold (minimum interval threshold, Fig 2D). The purpose of model optimization was to test different combinations of these parameters with the goal of maximizing the matches between “estimated” individual APCs identified by the accelerometer to the “actual” APCs observed from the video, including matching the exact timing of each APC.

Function 1 that identified each APC on the accelerometer was optimized without watching the video (independent optimization for a blind validation). Two-fold cross validation was used to partition the total dives with video for each animal into 50% training and 50% testing subsets (holdout method, [40,41]). To account for potential temporal, spatial, and prey distribution variation during a foraging trip, each dive with video was randomly assigned to either the training or testing subset. Each dive was only used once in the cross-validation process. Some dives were removed during the re-alignment of the datalogger clocks (see above), such that the actual proportion of dives in each subset ranged from 48–53% in the training subset (Table 1). Additionally, as the video camera only recorded for 1 hour every 4 hours when submerged > 40 m, video data was considered a random subsample of all dives. Overall, there were more dives that had prey present on video (74, 72 in each training or testing subset) compared to dives without potential prey present on video (22, 25 in each subset, Table 1).

Function 1 was optimized on the training subset of dives, (Table 1) using a range of variance thresholds and minimum interval thresholds. The variance threshold and minimum interval values selected on the training subsets were used to identify APCs within the testing subset for each animal [42]. The variance threshold values were selected based on: 1) visual examination of the raw acceleration on each axis; and 2) the dominant variance values in frequency histograms of the variance. The variance threshold values examined for sway and surge included 0.1, 0.2, 0.4, and 0.8. The heave axis had lower amplitude raw acceleration and lower variance of acceleration overall. Therefore, the variance thresholds tested were also lower (0.1, 0.2, and 0.4). A concurrent study on Australian fur seals outfitted with animal-borne video cameras determined that the mean APC duration was 18.7 ± 1.42 s (1653 APC, n = 18 animals, [38]). Therefore,5, 10, and 20 seconds were tested as potential minimum intervals.

The goal of optimizing Function1 using the training subset was to maximize detection and select the best parameters without prior knowledge of the video camera data. If multiple combinations of parameters subsequently resulted in similar detection within 2% (i.e. 95 vs. 97%), the combination with the highest precision (i.e. lowest FP rate) was used as the animal-specific parameters (indicated by * Fig 4). This was repeated for each axis and all animals. There was not a single set of variance threshold and minimum interval values that produced the greatest detection for all four animals.

Since it would not be feasible to parameterize Function 1 for every animal in a population, a preferred variance threshold and minimum interval is required to apply Function 1 to accelerometer data collected on other free-ranging Australian fur seals. Therefore, analysis was continued using two types of parameters: “animal-specific” parameters that yielded the greatest detection on each specific animal (parameters different for each animal), and “generic” parameters (same for all animals) that were selected based on preliminary analysis. The generic variance threshold of 0.1 was selected because all animals had the same animal-specific thresholds on surge and heave (i.e. 0.1 was the greatest detection on all animals heave and surge). The generic variance threshold was also set at 0.1 for sway because 2 of the 4 animals had the greatest detection at this threshold (also for consistency with the surge and heave). The generic minimum interval threshold of 5 s was selected because detection rates did not differ greatly between at 0.1 and 5 s vs. 0.2 and 5 s among the animal-specific parameters on each animal (Fig 4). Comparing detection and statistical results of the generic versus animal-specific parameters (using mixed-effects models described below) allowed us to determine if it was worth the additional data processing time and effort to optimize Function 1 for each animal or if the generic parameters gave similar results (i.e. minimal error in using the simple and faster “one-size-fits-all” generic parameters).

### Statistical Analysis

Statistical analysis was performed on the testing subset of data using the results of the optimization of Function 1 on the training dataset. To clarify, the training subset was used to select the animal-specific parameters by choosing the parameters with the greatest detection rate, followed by greatest precision. Next, the 50% testing subset was run on the animal-specific and generic parameters. All statistics and results below are reported for the 50% testing subset only, which was run on both generic and animal-specific parameters. Data from the testing subset were analyzed within a repeated measures framework using mixed-effects models in R 2.6.1 (nlme package, [35,43]). Mixed-effects models (LME) utilize individual animal variation relative to the mean of the population while correcting for repeated measurements within and among animals. Animal ID was treated as a random effect that allowed inferences beyond the sampled population. When the dependent variable is categorical, this is analogous to performing repeated measures ANOVA with the important addition of accounting for random effects. Statistical significance was set at α = 0.05. The significance of the fixed factor was determined using a conditional ANOVA F-Test, which also accounts for repeated measures and random effects. Model comparisons were performed using a likelihood ratio test (LRT) on two hierarchically nested models (the fixed effect model nested within the null model without a fixed effect). Tukey post hoc tests with Bonferroni adjusted p-values were used to compare the means between multiple levels within significant fixed factors (mvtnorm and multcomp R libraries).

The current study focused LME analysis of the testing subset on 4 specific questions that could be directly applied to predicting prey capture success and prey type with accelerometers on free-ranging animals (without cameras). First, LME models tested if error metrics (detection, precision, FP rate) varied between a fixed factor called function type (generic or animal-specific parameters) to determine if it was efficient to optimize a function for each animal. LME models were used to compare the generic versus animal-specific parameters across all prey types combined because Function 1 was optimized with all prey types combined (no *a priori* assumption of differences among prey). Second, LME models tested if there was a difference in error metrics among surge, sway, or heave to determine if one axis of acceleration was more useful for field application than the others. These two analyses assessed if the error of the accelerometer differed by parameters used or among axes of acceleration. The third question investigated if quantitative acceleration variables could be used to predict prey capture success. Fourth, LME models tested if quantitative acceleration variables could predict prey type on the testing subset. LME models were used to test if there were statistical differences in the number of peaks, integral area under the peaks, and duration of APC between successful events and unsuccessful events or among prey types.

## Results

### Analysis of error metrics on testing subset

Optimizing Function 1 with animal-specific parameters did not yield significantly different error metrics (detection, precision, FP rate) compared to the error metrics of the generic parameters on any axis (Table 3). There was overall greater detection, but conversely greater error (FP rate) for the generic parameters relative to the animal-specific parameters. However this difference was not significant on any metric or any axis of acceleration. On the surge axis for example, detection was similar for generic parameters and animal-specific parameters (Table 3, LRT = 1.4, P = 0.233). FP rate was also similar for the generic parameters relative to the animal-specific parameters (Table 3, LRT = 2.8, P = 0.096). Results were similar for detection and FP rate between generic and animal-specific comparisons on the sway and heave axis (Table 3).

Surge and sway had greater detection rates, but also greater error compared to heave (Table 3).

Detection significantly varied among surge, sway, and heave on the generic parameters (LRT = 12.5, P = 0.002). Detection was similar for surge and sway (Tukey, P = 1.0), but both surge and sway were significantly greater than heave detection rate (Tukey, P = 0.001 on all comparisons, generic parameters). FP rate was significantly greater for surge and sway compared to heave (LRT = 13.8, P = 0.001; Tukey, P = 0.05 for surge vs. sway, and P < 0.01 for comparisons vs. heave, generic parameters). Results were the same for detection and FP rate using the animal-specific parameters. Considering that there was no statistical difference between the mean error metrics calculated from the animal-specific or generic parameters (2 of the 4 animals had identical generic and animal-specific parameters), analysis focused on the results from the generic parameters to maximize the applicability of this method in the field. Unless noted otherwise, all of the data described subsequently are the testing subset of data using the generic parameters.

Each error metric was calculated across all dives for each animal, and then these numbers were averaged per each axis of acceleration for each animal (Table 3). The surge axis identified 327 APC total (total includes prey present and prey absent APC, Table 3, generic parameters). Based on video observations and post hoc comparison of video to accelerometer data, peaks in surge acceleration represented more than one specific behavioural category on the video including when the animal increased speed to chase prey (start of chase), the initial thrust when the animal first grabbed at the prey item (capture attempt), or prey handling. The surge axis detected 96.3% of the events with prey present (detection or TP rate). The surge axis misclassified 48.1% of the events as TP when there was no prey present on video (FP Rate, Table 3).

Overall, the sway axis showed similar trends and error metrics as surge (Table 3). The sway axis identified a greater number of APC than the surge axis with 402 events total. Video observations indicated that peaks in the sway acceleration occurred during prey handling and when the animal changed swimming patterns to chase prey. The sway axis detected 96.8% of the events with prey present, but misclassified 58.6% of the events as TP when there was no prey present on video (FP Rate). The heave axis detection (82.3%) was significantly lower than surge or sway, but heave misclassified proportionally less of the events (32.8% FP rate). Detection also did not differ by maximum APC per dive (LRT = 1.7, P = 0.19) or by mean APC per dive (LRT = 0.24, P = 0.62) on any acceleration axis (i.e. detection did not improve when there were less prey per dive when all prey types were included). Video and raw data observations showed that peaks in heave acceleration were lower in amplitude and less pronounced compared to the sway and surge. These peaks in heave acceleration represented either flipper strokes, or minor head movements during capture and handling.

### Accelerometer unable to differentiate successful from unsuccessful prey capture events

**Fig 5:**
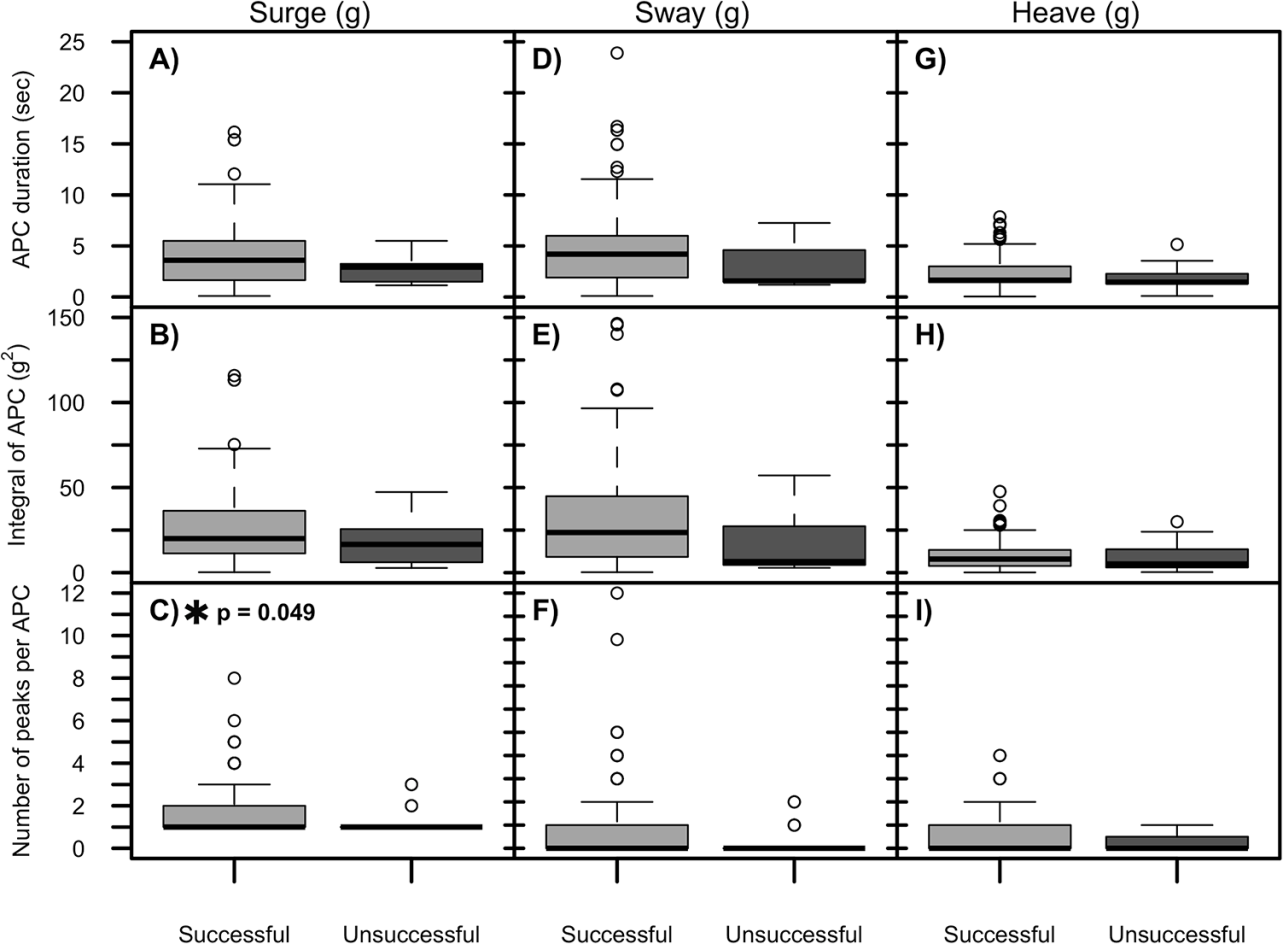
Analysis of successful prey captures by accelerometers. The accelerometer was not able to distinguish between successful and unsuccessful attempted prey captures (APC) on any axis of acceleration. Successful events included video where prey was captured, and unsuccessful events included events where the fur seal chased and missed an attempted capture attempt of the prey on video. Neither mean event duration (A, D, G), or integral area under the APC (B, E, H) significantly varied between successful or unsuccessful APC. The number of peaks in surge marginally differed between types of APC (C), but not for sway (F) or heave (I). Results are from Function 1 optimized with generic parameters (0.1 variance and 5 s). Each data point represents an individual APC.

Video analysis of the testing subset showed greater predicted success rates on surge (92.3 ± 8.0%), sway (92.5 ± 8.5%), and heave (93.8 ± 4.7%, mean on all axis = 93%) than previous video analysis on the same species (64%, [38]). On all three axes, successful events were generally longer in duration with greater amount of head movement than unsuccessful events, but these differences were only marginally significant on the surge axis and all ranges overlapped for each variable tested (Fig 5, each data point equals 1 individual APC). For example, on the surge axis, mean duration of successful APC (4.1 ± 2.7 s, n = 229) was not different than mean duration of unsuccessful APC (2.7 ± 1.2 s, n = 22, LRT = 2.25, P = 0.13). Mean integral area under the peak of variance did not differ between successful or unsuccessful APC (25.9 ± 20.3 vs. 17.4± 12.7 g^2^, LRT = 2.08, P = 0.15). Mean number of peaks per APC was marginally greater for successful APC, but the ranges of successful and unsuccessful APC overlapped thus preventing use of this variable to identify successful APC in the field (1.7 ± 1.1 vs. 1.3 ± 0.6, LRT = 3.86, P = 0.049). APC that included handling were also not significantly longer in duration (LRT = 0.47, P = 0.49), and did not have greater integral area under the peak compared to events without prey handling (LRT = 0.33, P = 0.57). On the sway and heave axis, neither mean duration of APC, mean peaks per APC, or mean integral area under the peak significantly varied between successful and unsuccessful events (Fig 5D–5I) This analysis was performed only on APC with prey present on the surge axis generic parameters.

On the surge axis, detection was similar for successful events (95.8 ± 3.6%) compared to unsuccessful events (100 ± 0%, ANOVA, F = 5.2, P = 0.12, each data point equals average per animal). Mean FP rate was greater for unsuccessful events (73.8 ± 23.2%) than for successful events (49.3 ± 15.0%), but this difference was not significant on the surge axis (LRT = 3.84, P = 0.050). The similar detection rates indicate that the surge axis has equal ability to identify for both successful and unsuccessful events when there is a capture attempt. The error metrics also did not differ significantly between successful and unsuccessful events on sway or surge.

### Accelerometer unable to differentiate among prey types

**Fig 6:**
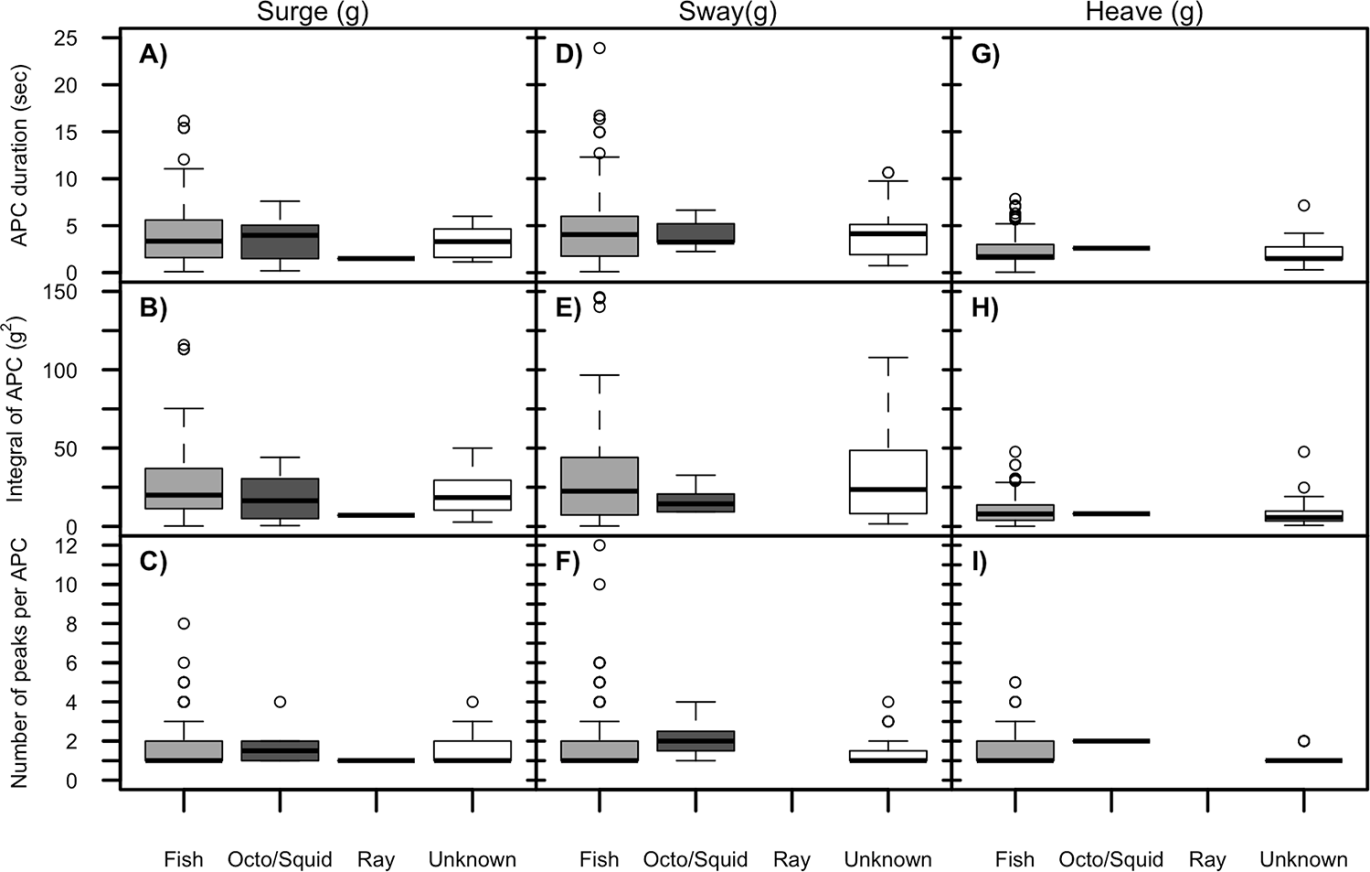
Analysis of prey types by accelerometers. The accelerometer was not able to distinguish among prey types. Neither mean APC duration (A, D, G), integral area under the APC (B, E, G), or number of variance peaks per APC (C, F, I) varied among prey types on any axis of acceleration. Results are from Function 1 optimized with generic parameters (all same per animal). Each data point represents an individual APC.

The prey composition data analysis included potential prey of both successful and unsuccessful attempts (Fig 6). The majority of potential prey identified by the accelerometer were fish (80.5%, 82.3%, 80.4% surge, sway, heave). Video analysis for a larger dataset showed that benthic gurnard fish were the predominant taxonomic family (n = 18 seals, 1653 APC, [38]). Unknown prey comprised 15.9%, 15.4%, and 0% of the prey identified on the surge (n = 251 total), sway (n = 305 total), and heave (n = 189 total) axes respectively. Octopus (*Octopus sp*) and squid (*Squid sp*.*)* represented only a small proportion of prey (0.5–3,2%), and therefore it is not surprising that subsequent analyses (using either mean APC duration, integral area under the peak of variance of acceleration, or number of peaks per APC, Fig 6) were unable to discriminate among prey types on any of the 3 acceleration axes.

Across surge (72.3%), sway (68.9%), and heave (74.3%) the majority of fish were consumed on the sea floor, but all squid, octopus, and stingrays were consumed on ascent or consumed at the surface. Video analysis showed that fish consumed on ascent were usually either relatively larger fish species or they were captured at the end of a dive after several other APC. Within the fish only on the surge axis, the accelerometer was still unable to differentiate between successful or unsuccessful APC using event duration (LRT = 1.61, P = 0.20), integral area under the peak (LRT = 1.54, P = 0.22), or number of peaks (LRT = 3.64, P = 0.056, similar results on sway and heave).

## Discussion

Direct observation of foraging behaviour is not possible for most marine mammals. Direct observation of foraging through animal-borne video cameras has recently been possible, but this is limited by battery life, memory capacity, and the size of the camera. There has been much development in the use of accelerometers to predict foraging behaviour because of their relatively small size and lower cost compared to animal-borne video. Accelerometers are also subject to battery and memory limits, although this could potentially be mitigated by only sampling one axis of acceleration. Results demonstrated that head-mounted accelerometers could identify APC, but were unable to distinguish among prey types or between successful captures and unsuccessful capture attempts. There was a significant difference in the ability of each axis to detect APC with surge and sway being equivalent and better than heave. Most notable for field application (and similar to, [24]), not all head movements at depth were related to actual APC on video as previously assumed with other studies [22,31,42]. This indicates that FP rate correction factors should be applied during field application of this method.

### Factors influencing detection and false positive rate

FP occurred when the accelerometer indicated a prey capture event that did not exist on video. Each animal had FP that occurred because head movements were not related to feeding behaviour on video and because the accelerometer matched multiple APC to a single video APC.

Video observations showed that the seals slowly move their head from side to side (sway) when searching for prey. This motion appeared much slower and more consistent than the faster sway movement observed on the video during handling. The current study measured variance in sway acceleration rather than absolute sway acceleration. It is possible that the increased sway during normal searching behaviour could increase the acceleration “noise” and, thus, decreased the acceleration variance when a greater peak in acceleration occurred during handling. It is probable that movements in the sway axis during searching influenced the greater number of FP for sway relative to heave (Table 3). It is also possible that the gain or “clipping” settings of ± 3g set during manufacturing of the datalogger influenced the detection or FP rates (Fig S2 in, [29]). Although a lower gain of ± 3g could have reduced the maximum-recorded amplitude of some of the larger peaks, the methods used in the current study appear robust enough to still yield detection rates 82–97% (Table 3). Video also showed “head bobs” in the heave axis during searching behaviour that were potentially linked to flipper strokes, although the heave axis did not show an increased FP rate. The rates of FP observed in the present study are greater than those obtained on southern elephant seals when accelerometers were compared to assumed foraging via dive shapes (0–21%, [42]), and also greater than the FP rate on captive Steller sea lions in the surge axis (13.3%, [31]). While validations on captive animals such as Viviant et al. [31] provide valuable data they can potentially result artificially lower FPs due to decreased variability in head movement caused by the animals consuming dead prey in a controlled pool environment, with 1 prey per dive.

FP rates are also influenced by the total number of potential events with prey present (TP+FP). If there are fewer total events with potential prey, then there will be a lower rate of FP because there are less total positives to be classified as FP or TP. On the surge axis, the present study had a greater FP rate (48.1%) and also a greater proportional number of APC with prey present (Table 1) compared to a similar study on Steller sea lions (FP rate 13.3%, 37% of 51 total APC had prey present, [31]) likely contributing to a higher FP rate in the current study. The lower FP rates observed in captive and wild penguins feeding on fish (0.09–9.8%, [30]) could be due to the lack of live prey as well as differences in study species foraging movements and accelerometer position.

FP rate for presence or absence of prey is distinct from FP rate for identification of each individual APC. Previous research on a single trained Steller sea lion reported < 1% FP rates [28], although this was based on only the presence or absence of prey (not individual APC) and was relative to the first animal’s predictive model tested on a second animal, not relative to video. Recalculating FP rate from the events where prey was present (FP rate = 100- precision), yields FP rates of 53.4% and 56.5% for Skinner et al. [28] which are comparable to those observed in our study (Table 3).

Maximum mean detection rate on surge was 96.3%, and the maximum on sway was 96.8% (Table 3). These are greater than the detection calculated using a similar function on a single captive Steller sea lion [31].Notably, the mean detection for dives with only 1 fish APC per dive in the present study (72 ± 28%) was greater than the detection for 1 dead fish per dive (68.4%, [31]). However, the Viviant et al. study differed from the present study in three key aspects: the state of prey (alive or dead); visibility of prey, and number of prey per dive. A major difference between the current study and previous validation studies is the presence of multiple prey items. (range 1–7, mean = 2.6 ± 1.6). Even examining on the events with fish prey, 78% of all APC identified by the surge axis had more than 1 prey per dive. Mean detection was highly variable for dives with 1 fish PCE per dive (72 ± 28%, 33–100% range), but was constant at 100% for dives with 2–7 fish per dive. FP rate oscillated with the number of prey per dive, indicating the number of prey per dive only impacts the detection when there is 1 APC per dive, but has little effect on FP rate. Hence, the greater range of prey items could potentially explain the greater detection rates in the current study compared to other studies [28,31].

Ultimately, differences among studies (primarily number of prey per dive, live or dead fish, and wild versus captive settings) likely combined with inter and intra-animal variation to influence the FP rate and detection when using head-accelerometers to predict APC. The comparable detection ranges for individual animals (67–100%, S1 Table) compared to the mean across all animals (82–97%, Table 3) indicates that head accelerometers are able to account for the inter-animal variation that occurs in free-ranging populations on multiple animals. Inter-animal variation plays a key role in accurately modeling foraging behaviour of wild populations, but studies that only have 1 animal are unable to account for this random variation due to differences among animals. Including multiple animals as random effects within mixed-effect models allowed us to account for this inter-animal variation and also make inferences beyond the study sample. In order for the accelerometer to correctly detect APC on video, there needs to be a consistent pattern in head movements not only within a single animal, but also among several animals, which was observed in the current study. Adding the additional error from inter-animal variation produced error rates that are likely more realistic when applying this method to the larger wild population.

This study endeavored to select the parameters of Function 1 using a quantitative and objective cross-validation method so that parameter selection could be repeated on animals without accompanying video data. No single function is error free, due to the great variability of circumstances and behaviours it needs to quantify simultaneously. The current study chose to maximize detection primarily, followed by precision (i.e. minimizing FP rate), but future studies could choose to optimize the function using other metrics. The specific metric used to optimize a function would vary based on predator and prey behaviour, as well as the specific research question and types of error researchers are willing to assume. This study focused analysis on using surge, sway, or heave individually to predict APC, but future research could explore if a single index of movement improves detection or FP rate. It’s possible that combining all 3 axis into a single index such as overall-dynamic body acceleration (ODBA), or Euclidean distances could improve the detection and FP rate for future research.

### Identification of successful prey captures

We hypothesized that the successful APC would have longer durations, greater integral areas under the peak of variance, and more peaks per event due to handling of the prey, but only peaks per APC marginally varied among successful or unsuccessful events on any axis. Because the range of peaks per APC overlapped, it was not possible to distinguish successful peaks apart using only this variable. These results agree with a recent study that was also unable to distinguish successful from unsuccessful APC using lower jaw acceleration on Antarctic fur seals, *Arctocephalus gazella*, although this study was unable to validate their method due to poor quality camera images [24]. Based on video observations, individual peaks per each APC likely represented the multiple head movements in a side-to-side motion while the seal was handling the prey. Examining the successful APC of fish on the sway axis (axis that showed the most handling on video) revealed that 90% of the successful APC included prey handling. The remaining 10% of successful events were classified as “chase and capture” with no handling present on video. On the sway axis within the successful APC, the range of peaks per APC was greater for events with handling (1–8 peaks) compared to successful events without handling (1–5 peaks), but the mean values did not differ enough for this to be statistically significant (handling mean = 1.8 ± 1.2 peaks; no handling mean = 1.6 ± 1.1 peaks, LRT = 0.55, P = 0.46). APC that included handling were not longer in duration and did not have greater integral areas under the peak compared to events without prey handling. This suggests that differences in APC duration, number of peaks per APC, or integral areas with handling did not mask potential differences between successful and unsuccessful APC. Future research could partition the APC into more detailed behavioural phases (chase, capture, handling) and test if the duration or presence of each phase could be used to distinguish successful APC.

### Field application

Practically, the generic parameters of Function 1 are more relevant in field application because they would allow future researchers to know in advance which specific variance threshold and minimum duration interval to use for this species, rather than having to deploy video cameras and determine a new set of animal-specific values. Future studies validating head-mounted accelerometers could reduce data analysis time without significantly increasing error by using a single set of generic parameters on all animals, but only if the parameters are selected quantitatively from among all animals. Future research could potentially add on-board processing of acceleration data with Function 1 to manufactured tags to reduce memory required for archival dataloggers or to condense data that can be relayed via telemetry.

Surge and sway had similar detection and FP rates, and were significantly better than heave for maximizing detection and total APC. However, each axis likely identified different parts of the APC. Based on video analysis, surge identified the initial head strike and sway identified the prey handling. Previous studies have found that surge is the most useful in identifying prey captures in Steller sea lions [31] and hooded seals [27], but no difference between surge and sway was found in Weddell seals [22]. Considering that 90% of all successful APC included prey handling, future field applications could use either surge or sway depending on the prey and predator species and which phase of the APC is of interest. Accelerometers were unable to distinguish among prey types due to low prey diversity. Most of the APC analyzed were fish (80–82%), which is consistent with research indicating that fish dominate Australian fur seal diets in the winter and cephalopods dominate in the summer [44]. We hypothesize that a greater diversity in prey consumed would permit us to differentiate among prey with different handling characteristics.

### Estimation of foraging dives

Using accelerometers to identify foraging dives and multiplying by the mean number of APC per dive is an alternate approach that could be used to estimate the total number of APC without the analysis to identify each individual APC. However, this method is only possible if the foraging dive identification error and average APC per dive are previously known from concurrent acceleration and video analysis. Foraging dives included all dives with at least 1 APC incorporating both successful and unsuccessful APC and dives with multiple APC. Non-foraging dives were dives that had no attempted captures during the entire dive on video, although they included searching behaviour and prey chases without capture attempts. All prey types and categories of success (unsuccessful or successful) were re-examined at the level of the dive for this separate analysis (i.e. same dataset but organized across each dive rather than each individual APC like above). On all animals, video identified 71 foraging dives and 26 non-foraging dives (n = 97 dives total in testing subset, Table 1). The surge axis identified 70 foraging dives and 27 non-foraging dives (n = 97 dives total with generic parameters). At the level of the dive head-mounted accelerometers underestimated the number of foraging dives by 1.4% on the surge axis (foraging dive identification error).

## Conclusion

Results demonstrated that it was possible to identify individual APC on free-ranging pinnipeds with a head-mounted accelerometer using an automated and quantitative parameter selection process. Head-mounted accelerometers can be used to estimate the number of APCs in free-ranging Australian fur seals, but this method required post processing to correct clock misalignment errors. Appropriate detection correction factors can be applied whether at the scale of each individual APC or the scale of each dive to offset the detection (82–97%) and FP rates (26–59%, Table 3) of this method. The current study strived to provide transparent data analysis details so that future research can expand on the quantitative approach of parameter selections and cross-validation to hopefully improve the reliability and detection rates of this method for field use. Accelerometers were unable differentiate among prey types or between successful or unsuccessful APCs using the methods tested. The number of prey successfully captured (out of attempted captures) could be estimated using the mean success rate of 64% from video analysis [38] or the axis specific success rates of the present study (92,93,94% for surge, sway, heave). The number of prey identified varied depending on both the function parameters set and the axis of acceleration used with the surge and sway axis being most useful for field application. Furthermore, results demonstrated that a single peak in acceleration variance did not equal an individual prey item and not all mouth openings were feeding activity. This highlights the importance of using a quantitative and repeatable parameter selection method for functions used to identify foraging behaviour in the future.

## Supporting Information

S1 Table Summary of accelerometer error metrics relative to video data for individual Australian fur seals on the testing subset. Summary of accelerometer error metrics relative to video data calculated for each animal illustrating inter and intra-animal variability in accelerometer metrics on the surge axis. Generic parameters were set at 0.1variance threshold with 5 second minimum interval for all animals and acceleration axis. Animal-specific parameters were those that yielded the greatest detection rate for each animal. Function 1 was first optimized on the training subset and statistics were executed on testing subset. Data are from the Random Testing subset.
(PDF)

S2 Table Acceleration, depth, and prey capture data from testing subset. Data from head-mounted 3D accelerometers, time-depth recorders (TDRs) and animal-borne video in Australian fur seals (n = 4 animals). Accelerometers were used to identify individual attempted prey captures (APC), and animal-borne video data were used to independently verify APC and prey types. APC were identified by peaks in the variance of acceleration using the variance threshold and the minimum interval between consecutive APC (Function 1, see methods). Metric classes were calculated by comparing each individual APC identified by the accelerometer (estimate) verses APC identified in the video (actual value, with 0 = absent and 1 = present, see methods). Dataset provided is for the generic parameters of Function 1 on the testing subset of data.
(PDF)

S1 Text Description of Function 1 that identified attempted prey captures (APC) and details on how video was matched to accelerometer data. (DOCX)
