# Supplementary material for: Identification of Prey Captures in Australian Fur Seals (Arctocephalus pusillus doriferus) Using Head-Mounted Accelerometers: Field Validation with Animal-Borne Video Cameras
Source: PLoS One. 2015 Jun 24;10(6):e0128789. doi: 10.1371/journal.pone.0128789 (PMC4479472; doi:10.1371/journal.pone.0128789)
Supplement: S1 Table — Summary of accelerometer error metrics relative to video data calculated for each animal illustrating inter and intra-animal variability in accelerometer metrics on the surge axis. Generic parameters were set at 0.1variance threshold with 5 second minimum interval for all animals and acceleration axis. Animal-specific parameters were those that yielded the greatest detection rate for each animal. Function 1 was first optimized on the training subset and statistics were executed on testing subset. Data are from the Random Testing subset. (PDF) [file pone.0128789.s001.pdf]

Supporting information

S1 Table. Summary of accelerometer error metrics relative to video data for individual Australian fur seals on the testing subset. Summary of accelerometer error metrics relative to video data calculated for each animal illustrating inter and intra-animal variability in accelerometer metrics on the surge axis. Generic parameters were set at 0.1variance threshold with 5 second minimum interval for all animals and acceleration axis. Animal-specific parameters were those that yielded the greatest detection rate for each animal. Function 1 was first optimized on the training subset and statistics were executed on testing subset. Data are from the Random Testing subset.

		Function 1	Variance	Minimum	Total	Total	Averaged over all dives per animal (%)		
Animal	Acceleration	Parameters	Threshold	Interval (sec)	Dives	APC	Detection	FP rate	Precision
W1855	X Surge	Animal-specific	0.1	10	24	45	90.5	47.2	52.8
W1855		Generic	0.1	5	24	59	100.0	59.6	40.4
W1859		Animal-specific	0.1	10	15	38	92.3	31.4	68.6
W1859		Generic	0.1	5	15	45	92.3	42.9	57.1
W1873		Animal-specific=Generic	0.1	5	40	108	97.1	28.7	71.3
W1881		Animal-specific=Generic	0.1	5	18	115	95.7	61.1	38.9
W1855	Y Sway	Animal-specific	0.2	10	24	38	85.7	33.3	66.7
W1855		Generic	0.1	5	24	55	100.0	56.3	43.8
W1859		Animal-specific	0.2	10	15	35	84.6	26.7	73.3
W1859		Generic	0.1	5	15	59	92.3	57.9	42.1
W1873		Animal-specific=Generic	0.1	5	40	151	97.1	53.5	46.5
W1881		Animal-specific=Generic	0.1	5	18	137	97.8	66.9	33.1
W1855	Z Heave	Animal-specific	0.1	10	24	34	81.0	15.0	85.0
W1855		Generic	0.1	5	24	42	85.7	37.9	62.1
W1859		Animal-specific	0.1	10	15	31	69.2	18.2	81.8
W1859		Generic	0.1	5	15	33	66.7	21.7	78.3
W1873		Animal-specific=Generic	0.1	5	40	98	85.5	21.3	78.7
W1881		Animal-specific=Generic	0.1	5	18	89	91.3	50.0	50.0
