# Supplementary material for: Identification of Prey Captures in Australian Fur Seals (Arctocephalus pusillus doriferus) Using Head-Mounted Accelerometers: Field Validation with Animal-Borne Video Cameras
Source: PLoS One. 2015 Jun 24;10(6):e0128789. doi: 10.1371/journal.pone.0128789 (PMC4479472; doi:10.1371/journal.pone.0128789)
Supplement: S2 Table — Data from head-mounted 3D accelerometers, time-depth recorders (TDRs) and animal-borne video in Australian fur seals (n = 4 animals). Accelerometers were used to identify individual attempted prey captures (APC), and animal-borne video data were used to independently verify APC and prey types. APC were identified by peaks in the variance of acceleration using the variance threshold and the minimum interval between consecutive APC (Function 1, see methods). Metric classes were calculated by comparing each individual APC identified by the accelerometer (estimate) verses APC identified in the video (actual value, with 0 = absent and 1 = present, see methods). Dataset provided is for the generic parameters of Function 1 on the testing subset of data. (PDF) [file pone.0128789.s002.pdf]

S2 Table. Acceleration, depth, and prey capture data from testing subset. Data from head-mounted 3D accelerometers, time-depth recorders (TDRs) and animal-borne video in Australian fur seals (n=4 animals). Accelerometers were used to identify individual attempted prey captures (APC), and animal-borne video data were used to independently verify APC and prey types. APC were identified by peaks in the variance of acceleration using the variance threshold and the minimum interval between consecutive APC (Function 1, see methods). Metric classes were calculated by comparing each individual APC identified by the accelerometer (estimate) verses APC identified in the video (actual value, with 0=absent and 1=present, see methods). Dataset provided is for the generic parameters of Function 1 on the testing subset of data.

Animal	Acceleration Axis	Data subset testing.generic	Variance Threshold	Minimum Interval (sec)	Video APC number	Accelerometer APC number	Dive Number	APC Depth (m)	Elapsed time of APC (sec)	Integral Value	Duration (sec)	Number of Peaks per APC	Total APC per dive on Video	Total APC per dive on Accelerometer	Dive Type	Prey Type	Peak Identified on Video	Peak Identified on Accelerometer	Behaviour	Success Type	Location of APC	Day or Night	Metric Class
W1855	surge.x	testing.generic	0.1	5	0	0	31	NA	NA	NA	NA	NA	NA	NA	control	no prey	0	0	no prey	no prey	no prey	Day	true.neg
W1855	surge.x	testing.generic	0.1	5	1	1	33	80.5	65.65	9.54	1.5	1	1	3	forage	unknown	1	1	chase.cap.hand	successful	ascent	Day	true.pos
W1855	surge.x	testing.generic	0.1	5	1	2	33	80.5	71.25	28.31	5.05	1	1	3	forage	unknown	0	1	chase.cap.hand	successful	ascent	Day	false.pos
W1855	surge.x	testing.generic	0.1	5	1	3	33	80.5	76.4	9.54	1.5	2	1	3	forage	unknown	0	1	chase.cap.hand	successful	ascent	Day	false.pos
W1855	surge.x	testing.generic	0.1	5	0	0	34	NA	NA	NA	NA	NA	NA	NA	control	no prey	0	0	no prey	no prey	no prey	Day	true.neg
W1855	surge.x	testing.generic	0.1	5	0	1	69	81	98.05	9.97	1.5	1	0	1	control	no prey	0	1	no prey	no prey	no prey	Night	false.pos
W1855	surge.x	testing.generic	0.1	5	0	1	71	81	97.15	4.56	1.45	1	0	1	control	no prey	0	1	no prey	no prey	no prey	Night	false.pos
W1855	surge.x	testing.generic	0.1	5	0	0	76	NA	NA	NA	NA	NA	NA	NA	control	no prey	0	0	no prey	no prey	no prey	Night	true.neg
W1855	surge.x	testing.generic	0.1	5	1	1	77	81	112.2	30.2	4.75	2	1	1	forage	unknown	1	1	chase.cap.hand	successful	ascent	Night	true.pos
W1855	surge.x	testing.generic	0.1	5	1	1	115	79.9	138.8	16.38	2.25	1	1	1	forage	fish	1	1	chase.cap	successful	ascent	Night	true.pos
W1855	surge.x	testing.generic	0.1	5	1	1	118	79.4	60.65	115.83	16.15	8	1	1	forage	fish	1	1	chase.cap.hand	successful	benthic	Night	true.pos
W1855	surge.x	testing.generic	0.1	5	1	1	120	79.4	90.65	5.95	1.5	1	2	7	forage	fish	1	1	chase.cap.hand	successful	benthic	Night	true.pos
W1855	surge.x	testing.generic	0.1	5	1	2	120	79.4	104.4	72.95	8.9	4	2	7	forage	fish	0	1	chase.cap.hand	successful	benthic	Night	false.pos
W1855	surge.x	testing.generic	0.1	5	1	3	120	79.4	120.55	6.52	1.55	1	2	7	forage	fish	0	1	chase.cap.hand	successful	benthic	Night	false.pos
W1855	surge.x	testing.generic	0.1	5	1	4	120	79.4	131.95	5.95	1.5	1	2	7	forage	fish	0	1	chase.cap.hand	successful	benthic	Night	false.pos
W1855	surge.x	testing.generic	0.1	5	2	5	120	79.4	178.05	72.95	8.9	1	2	7	forage	fish	1	1	chase.cap.hand	successful	ascent	Night	true.pos
W1855	surge.x	testing.generic	0.1	5	2	6	120	79.4	199	6.52	1.55	1	2	7	forage	fish	0	1	chase.cap.hand	successful	ascent	Night	false.pos
W1855	surge.x	testing.generic	0.1	5	2	7	120	79.4	204.35	5.95	1.5	1	2	7	forage	fish	0	1	chase.cap.hand	successful	ascent	Night	false.pos
W1855	surge.x	testing.generic	0.1	5	1	1	122	78.9	87.55	9.56	1.55	1	2	3	forage	unknown	1	1	chase(miss)	unsuccessful	benthic	Night	true.pos
W1855	surge.x	testing.generic	0.1	5	0	2	122	78.9	150.25	27.81	5.45	1	2	3	forage	no prey	0	1	no prey	no prey	no prey	Night	false.pos
W1855	surge.x	testing.generic	0.1	5	2	3	122	78.9	159.15	9.56	1.55	3	2	3	forage	fish	1	1	chase.cap	successful	ascent	Night	true.pos
W1855	surge.x	testing.generic	0.1	5	1	1	123	78.9	48.55	19.09	5.2	2	3	6	forage	fish	1	1	chase.cap	successful	benthic	Night	true.pos
W1855	surge.x	testing.generic	0.1	5	2	2	123	78.9	72.5	26.44	3.25	1	3	6	forage	unknown	1	1	chase.cap.hand	successful	benthic	Night	true.pos
W1855	surge.x	testing.generic	0.1	5	2	3	123	78.9	81.6	15.98	4.5	2	3	6	forage	unknown	0	1	chase.cap.hand	successful	benthic	Night	false.pos
W1855	surge.x	testing.generic	0.1	5	2	4	123	78.9	91.2	19.09	5.2	1	3	6	forage	unknown	0	1	chase.cap.hand	successful	benthic	Night	false.pos
W1855	surge.x	testing.generic	0.1	5	3	5	123	78.9	135	26.44	3.25	1	3	6	forage	fish	1	1	chase.cap.hand	successful	ascent	Night	true.pos
W1855	surge.x	testing.generic	0.1	5	3	6	123	78.9	143.5	15.98	4.5	3	3	6	forage	fish	0	1	chase.cap.hand	successful	ascent	Night	false.pos
W1855	surge.x	testing.generic	0.1	5	1	1	124	79.4	46.05	17.33	1.7	1	2	6	forage	fish	1	1	chase.cap.hand	successful	benthic	Night	true.pos
W1855	surge.x	testing.generic	0.1	5	1	2	124	79.4	53.25	35.4	6.5	1	2	6	forage	fish	0	1	chase.cap.hand	successful	benthic	Night	false.pos
W1855	surge.x	testing.generic	0.1	5	1	3	124	79.4	58.55	49.57	7.15	2	2	6	forage	fish	0	1	chase.cap.hand	successful	benthic	Night	false.pos
W1855	surge.x	testing.generic	0.1	5	2	4	124	79.4	69.95	2.49	1.2	3	2	6	forage	fish	1	1	chase.cap.hand	successful	benthic	Night	true.pos
W1855	surge.x	testing.generic	0.1	5	0	5	124	79.4	108.8	17.33	1.7	1	2	6	forage	no prey	0	1	no prey	no prey	no prey	Night	false.pos
W1855	surge.x	testing.generic	0.1	5	0	6	124	79.4	137.9	35.4	6.5	1	2	6	forage	no prey	0	1	no prey	no prey	no prey	Night	false.pos
W1855	surge.x	testing.generic	0.1	5	1	1	146	67.7	72.1	16.42	4.15	2	1	5	forage	octo	1	1	cap.hand	successful	ascent	Day	true.pos
W1855	surge.x	testing.generic	0.1	5	1	2	146	67.7	88.9	37.36	7.6	4	1	5	forage	octo	0	1	cap.hand	successful	ascent	Day	false.pos
W1855	surge.x	testing.generic	0.1	5	1	3	146	67.7	108.55	0.53	0.2	1	1	5	forage	octo	0	1	cap.hand	successful	ascent	Day	false.pos
W1855	surge.x	testing.generic	0.1	5	1	4	146	67.7	122.5	3.68	1.45	2	1	5	forage	octo	0	1	cap.hand	successful	ascent	Day	false.pos
W1855	surge.x	testing.generic	0.1	5	1	5	146	67.7	129.2	16.42	4.15	1	1	5	forage	octo	0	1	cap.hand	successful	ascent	Day	false.pos
W1855	surge.x	testing.generic	0.1	5	0	0	166	NA	NA	NA	NA	NA	NA	NA	control	no prey	0	0	no prey	no prey	no prey	Day	true.neg
W1855	surge.x	testing.generic	0.1	5	0	0	167	NA	NA	NA	NA	NA	NA	NA	control	no prey	0	0	no prey	no prey	no prey	Day	true.neg
W1855	surge.x	testing.generic	0.1	5	0	1	168	76.9	72.65	5.47	2.25	4	0	4	control	no prey	0	1	no prey	no prey	no prey	Day	false.pos
W1855	surge.x	testing.generic	0.1	5	0	2	168	76.9	148.55	4.5	1.75	1	0	4	control	no prey	0	1	no prey	no prey	no prey	Day	false.pos
W1855	surge.x	testing.generic	0.1	5	0	3	168	76.9	193.1	5.47	2.25	1	0	4	control	no prey	0	1	no prey	no prey	no prey	Day	false.pos
W1855	surge.x	testing.generic	0.1	5	0	4	168	76.9	208.6	4.5	1.75	2	0	4	control	no prey	0	1	no prey	no prey	no prey	Day	false.pos
W1855	surge.x	testing.generic	0.1	5	1	1	171	77.9	58.25	15.55	4.6	3	1	1	forage	fish	1	1	chase.cap.hand	successful	benthic	Day	true.pos
W1855	surge.x	testing.generic	0.1	5	1	1	172	77.9	91.45	23.53	5.95	1	1	3	forage	squid	1	1	chase.cap	successful	ascent	Day	true.pos
W1855	surge.x	testing.generic	0.1	5	1	2	172	77.9	98.6	44.1	3.8	2	1	3	forage	squid	0	1	chase.cap	successful	ascent	Day	false.pos
W1855	surge.x	testing.generic	0.1	5	1	3	172	77.9	115.75	6.36	1.55	1	1	3	forage	squid	0	1	chase.cap	successful	ascent	Day	false.pos
W1855	surge.x	testing.generic	0.1	5	0	0	173	NA	NA	NA	NA	NA	NA	NA	control	no prey	0	0	no prey	no prey	no prey	Day	true.neg
W1855	surge.x	testing.generic	0.1	5	1	1	204	76	78.5	0.26	0.1	1	2	6	forage	fish	1	1	chase.cap.hand	successful	benthic	Night	true.pos
W1855	surge.x	testing.generic	0.1	5	1	2	204	76	83.75	48.81	4.85	1	2	6	forage	fish	0	1	chase.cap.hand	successful	benthic	Night	false.pos
W1855	surge.x	testing.generic	0.1	5	2	3	204	76	104.65	11.02	1.55	1	2	6	forage	fish	1	1	chase.cap.hand	successful	benthic	Night	true.pos
W1855	surge.x	testing.generic	0.1	5	2	4	204	76	110.95	34.37	5.1	1	2	6	forage	fish	0	1	chase.cap.hand	successful	benthic	Night	false.pos
W1855	surge.x	testing.generic	0.1	5	2	5	204	76	118	19.05	2.15	1	2	6	forage	fish	0	1	chase.cap.hand	successful	benthic	Night	false.pos
W1855	surge.x	testing.generic	0.1	5	2	6	204	76	166.5	3.24	1.3	1	2	6	forage	fish	0	1	chase.cap.hand	successful	benthic	Night	false.pos
W1855	surge.x	testing.generic	0.1	5	0	0	236	NA	NA	NA	NA	NA	NA	NA	control	no prey	0	0	no prey	no prey	no prey	Night	true.neg
W1855	surge.x	testing.generic	0.1	5	1	1	239	76.4	48.95	63.52	11.05	6	2	2	forage	fish	1	1	chase.cap.hand	successful	benthic	Night	true.pos
W1855	surge.x	testing.generic	0.1	5	2	2	239	76.4	126.75	14.98	1.85	1	2	2	forage	fish	1	1	chase.cap.hand	successful	ascent	Night	true.pos
W1855	surge.x	testing.generic	0.1	5	1	1	243	76.4	128.4	43.54	5.35	4	1	1	forage	fish	1	1	chase.cap	successful	ascent	Night	true.pos
W1855	sway.y	testing.generic	0.1	5	0	0	31	NA	NA	NA	NA	NA	NA	NA	control	no prey	0	0	no prey	no prey	no prey	Night	true.neg
W1855	sway.y	testing.generic	0.1	5	1	1	33	80.5	65.35	3.76	1.45	1	1	2	forage	unknown	1	1	chase.cap.hand	successful	ascent	Day	true.pos
W1855	sway.y	testing.generic	0.1	5	1	2	33	80.5	71.7	48.34	6.75	3	1	2	forage	unknown	0	1	chase.cap.hand	successful	ascent	Day	false.pos
W1855	sway.y	testing.generic	0.1	5	0	0	34	NA	NA	NA	NA	NA	NA	NA	control	no prey	0	0	no prey	no prey	no prey	Day	true.neg
W1855	sway.y	testing.generic	0.1	5	0	1	69	81	97.9	6.66	1.5	1	0	1	control	no prey	0	1	no prey	no prey	no prey	Night	false.pos
W1855	sway.y	testing.generic	0.1	5	0	0	71	NA	NA	NA	NA	NA	NA	NA	control	no prey	0	0	no prey	no prey	no prey	Night	true.neg
W1855	sway.y	testing.generic	0.1	5	0	0	76	NA	NA	NA	NA	NA	NA	NA	control	no prey	0	0	no prey	no prey	no prey	Night	true.neg
W1855	sway.y	testing.generic	0.1	5	1	1	77	81	113.35	24.03	5.8	1	1	1	forage	unknown	1	1	chase.cap.hand	successful	ascent	Night	true.pos
W1855	sway.y	testing.generic	0.1	5	0	1	115	79.9	53	3.04	1.35	1	1	2	forage	no prey	0	1	no prey	no prey	no prey	Night	false.pos
W1855	sway.y	testing.generic	0.1	5	1	2	115	79.9	138.75	20.25	1.8	1	1	2	forage	fish	1	1	chase.cap	successful	ascent	Night	true.pos
W1855	sway.y	testing.generic	0.1	5	1	1	118	79.4	61.7	94.94	12.3	3	1	2	forage	fish	1	1	chase.cap.hand	successful	benthic	Night	true.pos
W1855	sway.y	testing.generic	0.1	5	1	2	118	79.4	75.7	33.75	5.35	5	1	2	forage	fish	0	1	chase.cap.hand	successful	benthic	Night	false.pos
W1855	sway.y	testing.generic	0.1	5	1	1	120	79.4	90.45	9.95	1.9	1	2	7	forage	fish	1	1	chase.cap.hand	successful	benthic	Night	true.pos
W1855	sway.y	testing.generic	0.1	5	1	2	120	79.4	107.6	62.67	7.7	2	2	7	forage	fish	0	1	chase.cap.hand	successful	benthic	Night	false.pos
W1855	sway.y	testing.generic	0.1	5	1	3	120	79.4	122.2	69.59	11.55	3	2	7	forage	fish	0	1	chase.cap.hand	successful	benthic	Night	false.pos
W1855	sway.y	testing.generic	0.1	5	2	4	120	79.4	177.75	8.54	1.55	1	2	7	forage	fish	1	1	chase.cap.hand	successful	ascent	Night	true.pos
W1855	sway.y	testing.generic	0.1	5	2	5	120	79.4	187.15	9.95	1.9	1	2	7	forage	fish	0	1	chase.cap.hand	successful	ascent	Night	false.pos
W1855	sway.y	testing.generic	0.1	5	2	6	120	79.4	199	62.67	7.7	1	2	7	forage	fish	0	1	chase.cap.hand	successful	ascent	Night	false.pos
W1855	sway.y	testing.generic	0.1	5	2	7	120	79.4	204.3	69.59	11.55	1	2	7	forage	fish	0	1	chase.cap.hand	successful	ascent	Night	false.pos
W1855	sway.y	testing.generic	0.1	5	1	1	122	78.9	87.55	2.96	1.2	1	2	3	forage	unknown	1	1	chase(miss)	unsuccessful	benthic	Night	true.pos
W1855	sway.y	testing.generic	0.1	5	0	2	122	78.9	149.75	41.84	5.2	1	2	3	forage	no prey	0	1	no prey	no prey	no prey	Night	false.pos
W1855	sway.y	testing.generic	0.1	5	2	3	122	78.9	159.45	2.96	1.2	3	2	3	forage	fish	1	1	chase.cap	successful	ascent	Night	true.pos
W1855	sway.y	testing.generic	0.1	5	1	1	123	78.9	48.7	44.92	4.8	2	3	6	forage	fish	1	1	chase.cap	successful	benthic	Night	true.pos
W1855	sway.y	testing.generic	0.1	5	2	2	123	78.9	72.55	26.97	3.3	1	3	6	forage	unknown	1	1	chase.cap.hand	successful	benthic	Night	true.pos
W1855	sway.y	testing.generic	0.1	5	2	3	123	78.9	81.6	51.48	8.85	2	3	6	forage	unknown	0	1	chase.cap.hand	successful	benthic	Night	false.pos
W1855	sway.y	testing.generic	0.1	5	2	4	123	78.9	91.25	44.92	4.8	1	3	6	forage	unknown	0	1	chase.cap.hand	successful	benthic	Night	false.pos
W1855	sway.y	testing.generic	0.1	5	3	5	123	78.9	134.95	26.97	3.3	1	3	6	forage	fish	1	1	chase.cap.hand	successful	ascent	Night	true.pos
W1855	sway.y	testing.generic	0.1	5	3	6	123	78.9	145.65	51.48	8.85	3	3	6	forage	fish	0	1	chase.cap.hand	successful	ascent	Night	false.pos
W1855	sway.y	testing.generic	0.1	5	1	1	124	79.4	46.15	73.91	10.05	6	2	3	forage	fish	1	1	chase.cap.hand	successful	benthic	Night	true.pos
W1855	sway.y	testing.generic	0.1	5	2	2	124	79.4	69.95	69.65	8.6	2	2	3	forage	fish	1	1	chase.cap.hand	successful	benthic	Night	true.pos
W1855	sway.y	testing.generic	0.1	5	0	3	124	79.4	109.1	7.79	1.6	1	2	3	forage	no prey	0	1	no prey	no prey	no prey	Night	false.pos
W1855	sway.y	testing.generic	0.1	5	1	1	146	67.7	74.55	9.25	3.25	2	1	5	forage	octo	1	1	cap.hand	successful	ascent	Day	true.pos
W1855	sway.y	testing.generic	0.1	5	1	2	146	67.7	88.85	14.41	3.25	2	1	5	forage	octo	0	1	cap.hand	successful	ascent	Day	false.pos
W1855	sway.y	testing.generic	0.1	5	1	3	146	67.7	96.15	20.01	4.7	2	1	5	forage	octo	0	1	cap.hand	successful	ascent	Day	false.pos
W1855	sway.y	testing.generic	0.1	5	1	4	146	67.7	108.5	21.48	5.7	1	1	5	forage	octo	0	1	cap.hand	successful	ascent	Day	false.pos
W1855	sway.y	testing.generic	0.1	5	1	5	146	67.7	122.8	9.25	3.25	3	1	5	forage	octo	0	1	cap.hand	successful	ascent	Day	false.pos
W1855	sway.y	testing.generic	0.1	5	0	0	166	NA	NA	NA	NA	NA	NA	NA	control	no prey	0	0	no prey	no prey	no prey	Day	true.neg
W1855	sway.y	testing.generic	0.1	5	0	0	167	NA	NA	NA	NA	NA	NA	NA	control	no prey	0	0	no prey	no prey	no prey	Day	true.neg
W1855	sway.y	testing.generic	0.1	5	0	1	168	76.9	72.45	5.83	2.4	2	0	3	control	no prey	0	1	no prey	no prey	no prey	Day	false.pos
W1855	sway.y	testing.generic	0.1	5	0	2	168	76.9	141.55	2.43	0.9	1	0	3	control	no prey	0	1	no prey	no prey	no prey	Day	false.pos
W1855	sway.y	testing.generic	0.1	5	0	3	168	76.9	147.35	5.83	2.4	1	0	3	control	no prey	0	1	no prey	no prey	no prey	Day	false.pos
W1855	sway.y	testing.generic	0.1	5	1	1	171	77.9	59	20.55	4.1	3	1	1	forage	fish	1	1	chase.cap.hand	successful	benthic	Day	true.pos
W1855	sway.y	testing.generic	0.1	5	1	1	172	77.9	90.95	32.71	6.65	4	1	2	forage	squid	1	1	chase.cap	successful	ascent	Day	true.pos
W1855	sway.y	testing.generic	0.1	5	1	2	172	77.9	115.85	9.33	2.25	1	1	2	forage	squid	0	1	chase.cap	successful	ascent	Day	false.pos
W1855	sway.y	testing.generic	0.1	5	0	1	173	77.4	52.2	3.36	1.45	1	0	2	control	no prey	0	1	no prey	no prey	no prey	Day	false.pos
W1855	sway.y	testing.generic	0.1	5	0	2	173	77.4	208.1	2.84	1.2	2	0	2	control	no prey	0	1	no prey	no prey	no prey	Day	false.pos
W1855	sway.y	testing.generic	0.1	5	1	1	204	76	84.45	46.58	4.7	1	2	5	forage	fish	1	1	chase.cap.hand	successful	benthic	Night	true.pos
W1855	sway.y	testing.generic	0.1	5	2	2	204	76	104.45	12.75	1.85	1	2	5	forage	fish	1	1	chase.cap.hand	successful	benthic	Night	true.pos
W1855	sway.y	testing.generic	0.1	5	2	3	204	76	111.05	31.86	5.4	1	2	5	forage	fish	0	1	chase.cap.hand	successful	benthic	Night	false.pos
W1855	sway.y	testing.generic	0.1	5	2	4	204	76	118.15	14.94	1.8	1	2	5	forage	fish	0	1	chase.cap.hand	successful	benthic	Night	false.pos
W1855	sway.y	testing.generic	0.1	5	2	5	204	76	166.7	1.28	0.6	1	2	5	forage	fish	0	1	chase.cap.hand	successful	benthic	Night	false.pos
W1855	sway.y	testing.generic	0.1	5	0	0	236	NA	NA	NA	NA	NA	NA	NA	control	no prey	0	0	no prey	no prey	no prey	Night	true.neg
W1855	sway.y	testing.generic	0.1	5	1	1	239	76.4	53.9	90.18	10.5	5	2	2	forage	fish	1	1	chase.cap.hand	successful	benthic	Night	true.pos
W1855	sway.y	testing.generic	0.1	5	2	2	239	76.4	122.35	10.01	3.25	2	2	2	forage	fish	1	1	chase.cap.hand	successful	ascent	Night	true.pos
W1855	sway.y	testing.generic	0.1	5	1	1	243	76.4	128.25	66.32	6.85	4	1	1	forage	fish	1	1	chase.cap	successful	ascent	Night	true.pos
W1855	heave.z	testing.generic	0.1	5	0	0	31	NA	NA	NA	NA	NA	NA	NA	control	no prey	0	0	no prey	no prey	no prey	Night	true.neg
W1855	heave.z	testing.generic	0.1	5	1	1	33	80.5	65.3	3.17	1.45	1	1	3	forage	unknown	1	1	chase.cap.hand	successful	ascent	Day	true.pos
W1855	heave.z	testing.generic	0.1	5	1	2	33	80.5	71.6	6.04	1.5	1	1	3	forage	unknown	0	1	chase.cap.hand	successful	ascent	Day	false.pos
W1855	heave.z	testing.generic	0.1	5	1	3	33	80.5	79.9	4.92	1.5	1	1	3	forage	unknown	0	1	chase.cap.hand	successful	ascent	Day	false.pos
W1855	heave.z	testing.generic	0.1	5	0	0	34	NA	NA	NA	NA	NA	NA	NA	control	no prey	0	0	no prey	no prey	no prey	Day	true.neg
W1855	heave.z	testing.generic	0.1	5	0	0	69	NA	NA	NA	NA	NA	NA	NA	control	no prey	0	0	no prey	no prey	no prey	Day	true.neg
W1855	heave.z	testing.generic	0.1	5	0	0	71	NA	NA	NA	NA	NA	NA	NA	control	no prey	0	0	no prey	no prey	no prey	Day	true.neg
W1855	heave.z	testing.generic	0.1	5	0	0	76	NA	NA	NA	NA	NA	NA	NA	control	no prey	0	0	no prey	no prey	no prey	Day	true.neg
W1855	heave.z	testing.generic	0.1	5	1	1	77	81	114.4	0.65	0.3	1	1	1	forage	unknown	1	1	chase.cap.hand	successful	ascent	Night	true.pos
W1855	heave.z	testing.generic	0.1	5	1	1	115	79.9	138.7	4.81	1.5	1	1	1	forage	fish	1	1	chase.cap	successful	ascent	Night	true.pos
W1855	heave.z	testing.generic	0.1	5	1	1	118	79.4	61.75	30.64	7	3	1	2	forage	fish	1	1	chase.cap.hand	successful	benthic	Night	true.pos
W1855	heave.z	testing.generic	0.1	5	1	2	118	79.4	80.4	7.75	2.1	1	1	2	forage	fish	0	1	chase.cap.hand	successful	benthic	Night	false.pos
W1855	heave.z	testing.generic	0.1	5	1	1	120	79.4	90.6	5.15	1.5	1	2	5	forage	fish	1	1	chase.cap.hand	successful	benthic	Night	true.pos
W1855	heave.z	testing.generic	0.1	5	1	2	120	79.4	107.45	19.34	4.25	5	2	5	forage	fish	0	1	chase.cap.hand	successful	benthic	Night	false.pos
W1855	heave.z	testing.generic	0.1	5	1	3	120	79.4	120.75	5.65	1.6	1	2	5	forage	fish	0	1	chase.cap.hand	successful	benthic	Night	false.pos
W1855	heave.z	testing.generic	0.1	5	1	4	120	79.4	131.55	5.15	1.5	1	2	5	forage	fish	0	1	chase.cap.hand	successful	benthic	Night	false.pos
W1855	heave.z	testing.generic	0.1	5	2	5	120	79.4	198.9	19.34	4.25	1	2	5	forage	fish	1	1	chase.cap.hand	successful	ascent	Night	true.pos
W1855	heave.z	testing.generic	0.1	5	2	1	122	78.9	159.25	4.13	1.4	1	2	2	forage	fish	1	1	chase.cap	successful	ascent	Night	true.pos
W1855	heave.z	testing.generic	0.1	5	2	2	122	78.9	166.5	5.1	1.5	1	2	2	forage	fish	0	1	chase.cap	successful	ascent	Night	false.pos
W1855	heave.z	testing.generic	0.1	5	1	0	122	NA	NA	NA	NA	NA	NA	NA	forage	unknown	1	0	chase(miss)	unsuccessful	benthic	Night	false.neg
W1855	heave.z	testing.generic	0.1	5	1	1	123	78.9	50.7	7.14	1.6	1	3	4	forage	fish	1	1	chase.cap	successful	benthic	Night	true.pos
W1855	heave.z	testing.generic	0.1	5	2	2	123	78.9	84.6	7.6	1.5	1	3	4	forage	unknown	1	1	chase.cap.hand	successful	benthic	Night	true.pos
W1855	heave.z	testing.generic	0.1	5	2	3	123	78.9	91.05	9.41	1.55	1	3	4	forage	unknown	0	1	chase.cap.hand	successful	benthic	Night	false.pos
W1855	heave.z	testing.generic	0.1	5	3	4	123	78.9	143.25	2.6	1.25	1	3	4	forage	fish	1	1	chase.cap.hand	successful	ascent	Night	true.pos
W1855	heave.z	testing.generic	0.1	5	1	1	124	79.4	46.15	8.41	1.55	1	2	4	forage	fish	1	1	chase.cap.hand	successful	benthic	Night	true.pos
W1855	heave.z	testing.generic	0.1	5	1	2	124	79.4	53.35	9.48	2.9	1	2	4	forage	fish	0	1	chase.cap.hand	successful	benthic	Night	false.pos
W1855	heave.z	testing.generic	0.1	5	1	3	124	79.4	58.5	29	5.2	2	2	4	forage	fish	0	1	chase.cap.hand	successful	benthic	Night	false.pos
W1855	heave.z	testing.generic	0.1	5	2	4	124	79.4	70	8.41	1.55	4	2	4	forage	fish	1	1	chase.cap.hand	successful	benthic	Night	true.pos
W1855	heave.z	testing.generic	0.1	5	1	0	146	NA	NA	NA	NA	NA	NA	NA	forage	octo	1	0	cap.hand	successful	ascent	Day	false.neg
W1855	heave.z	testing.generic	0.1	5	0	0	166	NA	NA	NA	NA	NA	NA	NA	control	no prey	0	0	no prey	no prey	no prey	Day	true.neg
W1855	heave.z	testing.generic	0.1	5	0	0	167	NA	NA	NA	NA	NA	NA	NA	control	no prey	0	0	no prey	no prey	no prey	Day	true.neg
W1855	heave.z	testing.generic	0.1	5	0	0	168	NA	NA	NA	NA	NA	NA	NA	control	no prey	0	0	no prey	no prey	no prey	Day	true.neg
W1855	heave.z	testing.generic	0.1	5	1	0	171	NA	NA	NA	NA	NA	NA	NA	forage	fish	1	0	chase.cap.hand	successful	benthic	Day	false.neg
W1855	heave.z	testing.generic	0.1	5	1	1	172	77.9	98.6	8.12	2.6	2	1	1	forage	squid	1	1	chase.cap	successful	ascent	Day	true.pos
W1855	heave.z	testing.generic	0.1	5	0	0	173	NA	NA	NA	NA	NA	NA	NA	control	no prey	0	0	no prey	no prey	no prey	Day	true.neg
W1855	heave.z	testing.generic	0.1	5	1	1	204	76	83.85	8.23	2.7	1	2	3	forage	fish	1	1	chase.cap.hand	successful	benthic	Night	true.pos
W1855	heave.z	testing.generic	0.1	5	2	2	204	76	104.45	3.24	1.4	1	2	3	forage	fish	1	1	chase.cap.hand	successful	benthic	Night	true.pos

W1855	heave.z	testing.generic	0.1	5	2	3	204	76	110.95	18.39	2.95	1	2	3	forage	fish	0	1	chase.cap.hand	successful	benthic	Night	false.pos
W1855	heave.z	testing.generic	0.1	5	0	0	236	NA	NA	NA	NA	NA	NA	NA	control	no prey	0	0	no prey	no prey	no prey	Night	true.neg
W1855	heave.z	testing.generic	0.1	5	1	1	239	76.4	58.15	17.18	4.45	3	2	2	forage	fish	1	1	chase.cap.hand	successful	benthic	Night	true.pos
W1855	heave.z	testing.generic	0.1	5	2	2	239	76.4	127.25	0.3	0.1	1	2	2	forage	fish	1	1	chase.cap.hand	successful	ascent	Night	true.pos
W1855	heave.z	testing.generic	0.1	5	1	1	243	76.4	133.15	13.32	3	2	1	1	forage	fish	1	1	chase.cap	successful	ascent	Night	true.pos
W1859	surge.x	testing.generic	0.1	5	1	1	2	81.9	86.4	7.11	1.5	1	1	1	forage	stingray	1	1	chase.cap	successful	ascent	Day	true.pos
W1859	surge.x	testing.generic	0.1	5	1	1	157	83.3	59.75	13.7	1.7	1	3	4	forage	fish	1	1	chase.cap.hand	successful	benthic	Night	true.pos
W1859	surge.x	testing.generic	0.1	5	1	2	157	83.3	66.55	40.42	7.5	1	3	4	forage	fish	0	1	chase.cap.hand	successful	benthic	Night	false.pos
W1859	surge.x	testing.generic	0.1	5	2	3	157	83.3	87.95	17.49	3.2	5	3	4	forage	fish	1	1	chase.cap	successful	benthic	Night	true.pos
W1859	surge.x	testing.generic	0.1	5	3	4	157	83.3	118.35	13.7	1.7	2	3	4	forage	unknown	1	1	chase.cap.hand	successful	ascent	Night	true.pos
W1859	surge.x	testing.generic	0.1	5	1	1	167	83.3	56.15	19.55	3.6	3	3	5	forage	fish	1	1	chase.cap.hand	successful	benthic	Night	true.pos
W1859	surge.x	testing.generic	0.1	5	1	2	167	83.3	69.6	17.51	2.55	1	3	5	forage	fish	0	1	chase.cap.hand	successful	benthic	Night	false.pos
W1859	surge.x	testing.generic	0.1	5	1	3	167	83.3	76.1	19.55	3.6	1	3	5	forage	fish	0	1	chase.cap.hand	successful	benthic	Night	false.pos
W1859	surge.x	testing.generic	0.1	5	2	4	167	83.3	89.15	17.51	2.55	1	3	5	forage	fish	1	1	cap.hand	successful	benthic	Night	true.pos
W1859	surge.x	testing.generic	0.1	5	3	5	167	83.3	121.35	19.55	3.6	1	3	5	forage	fish	1	1	chase.cap.hand	successful	ascent	Night	true.pos
W1859	surge.x	testing.generic	0.1	5	1	1	168	83.3	90.6	6.5	1.5	1	2	2	forage	fish	1	1	chase.cap.hand	successful	benthic	Night	true.pos
W1859	surge.x	testing.generic	0.1	5	1	2	168	83.3	103.45	7.09	1.95	1	2	2	forage	fish	0	1	chase.cap.hand	successful	benthic	Night	false.pos
W1859	surge.x	testing.generic	0.1	5	2	0	168	NA	NA	NA	NA	NA	NA	NA	forage	fish	1	0	chase.cap.hand	successful	ascent	Night	false.neg
W1859	surge.x	testing.generic	0.1	5	1	1	169	83.8	50.7	4.1	1.45	1	2	3	forage	fish	1	1	chase.cap.hand	successful	benthic	Night	true.pos
W1859	surge.x	testing.generic	0.1	5	1	2	169	83.8	58.6	26.61	4.75	1	2	3	forage	fish	0	1	chase.cap.hand	successful	benthic	Night	false.pos
W1859	surge.x	testing.generic	0.1	5	2	3	169	83.8	129.6	4.1	1.45	3	2	3	forage	fish	1	1	chase.cap.hand	successful	ascent	Night	true.pos
W1859	surge.x	testing.generic	0.1	5	1	1	172	83.8	78.15	21.15	4.2	2	2	2	forage	fish	1	1	chase(miss)	unsuccessful	benthic	Night	true.pos
W1859	surge.x	testing.generic	0.1	5	2	2	172	83.8	127.4	27.09	4.5	2	2	2	forage	fish	1	1	chase.cap.hand	successful	ascent	Night	true.pos
W1859	surge.x	testing.generic	0.1	5	1	1	174	83.8	82.45	15.71	1.55	1	2	2	forage	fish	1	1	chase.cap.hand	successful	benthic	Night	true.pos
W1859	surge.x	testing.generic	0.1	5	2	2	174	83.8	119.15	2.77	1.15	1	2	2	forage	unknown	1	1	chase(miss)	unsuccessful	benthic	Night	true.pos
W1859	surge.x	testing.generic	0.1	5	1	1	261	84.5	119.7	15.13	1.6	1	1	2	forage	fish	1	1	cap.hand	successful	ascent	Day	true.pos
W1859	surge.x	testing.generic	0.1	5	1	2	261	84.5	130	4.88	1.5	1	1	2	forage	fish	0	1	cap.hand	successful	ascent	Day	false.pos
W1859	surge.x	testing.generic	0.1	5	1	1	263	84.5	65	22.24	3.25	2	2	4	forage	fish	1	1	chase.cap.hand	successful	benthic	Day	true.pos
W1859	surge.x	testing.generic	0.1	5	1	2	263	84.5	77.75	18.04	1.85	1	2	4	forage	fish	0	1	chase.cap.hand	successful	benthic	Day	false.pos
W1859	surge.x	testing.generic	0.1	5	2	3	263	84.5	97.1	22.24	3.25	1	2	4	forage	fish	1	1	chase(no cap)	unsuccessful	ascent	Day	true.pos
W1859	surge.x	testing.generic	0.1	5	2	4	263	84.5	108.05	18.04	1.85	1	2	4	forage	fish	0	1	chase.cap.hand	successful	ascent	Day	false.pos
W1859	surge.x	testing.generic	0.1	5	0	1	266	84.5	54.3	35	7.7	5	1	2	forage	no prey	0	1	no prey	no prey	no prey	Day	false.pos
W1859	surge.x	testing.generic	0.1	5	1	2	266	84.5	87.95	5.13	1.5	1	1	2	forage	fish	1	1	chase.cap	successful	ascent	Day	true.pos
W1859	surge.x	testing.generic	0.1	5	1	1	267	84.5	94.45	11.66	1.65	1	1	2	forage	fish	1	1	chase.cap.hand	successful	ascent	Day	true.pos
W1859	surge.x	testing.generic	0.1	5	1	2	267	84.5	99.55	15.84	1.55	1	1	2	forage	fish	0	1	chase.cap.hand	successful	ascent	Day	false.pos
W1859	surge.x	testing.generic	0.1	5	0	0	269	NA	NA	NA	NA	NA	NA	NA	control	no prey	0	0	no prey	no prey	no prey	Day	true.neg
W1859	surge.x	testing.generic	0.1	5	1	1	329	85.8	47.9	11.51	4.05	4	4	6	forage	fish	1	1	chase.cap.hand	successful	benthic	Night	true.pos
W1859	surge.x	testing.generic	0.1	5	2	2	329	85.8	70.45	13.87	2.25	1	4	6	forage	fish	1	1	cap.hand	successful	benthic	Night	true.pos
W1859	surge.x	testing.generic	0.1	5	2	3	329	85.8	83.25	28.98	2.65	1	4	6	forage	fish	0	1	cap.hand	successful	benthic	Night	false.pos
W1859	surge.x	testing.generic	0.1	5	4	4	329	85.8	119	11.51	4.05	1	4	6	forage	fish	1	1	chase.cap	successful	ascent	Night	true.pos
W1859	surge.x	testing.generic	0.1	5	4	5	329	85.8	135.2	13.87	2.25	2	4	6	forage	fish	0	1	chase.cap	successful	ascent	Night	false.pos
W1859	surge.x	testing.generic	0.1	5	4	6	329	85.8	146.8	28.98	2.65	1	4	6	forage	fish	0	1	chase.cap	successful	ascent	Night	false.pos
W1859	surge.x	testing.generic	0.1	5	3	0	329	NA	NA	NA	NA	NA	NA	NA	forage	fish	1	0	cap.hand	successful	benthic	Night	false.neg
W1859	surge.x	testing.generic	0.1	5	0	1	335	85.8	52.15	4.51	1.45	1	1	2	forage	no prey	0	1	no prey	no prey	no prey	Night	false.pos
W1859	surge.x	testing.generic	0.1	5	1	2	335	85.8	79.65	14.16	1.7	1	1	2	forage	fish	1	1	chase.cap.hand	successful	ascent	Night	true.pos
W1859	surge.x	testing.generic	0.1	5	1	1	336	85.8	83.6	4.25	1.5	1	1	5	forage	fish	1	1	chase.cap.hand	successful	ascent	Night	true.pos
W1859	surge.x	testing.generic	0.1	5	1	2	336	85.8	104	11.85	3.05	2	1	5	forage	fish	0	1	chase.cap.hand	successful	ascent	Night	false.pos
W1859	surge.x	testing.generic	0.1	5	1	3	336	85.8	123.95	57.58	7.4	1	1	5	forage	fish	0	1	chase.cap.hand	successful	ascent	Night	false.pos
W1859	surge.x	testing.generic	0.1	5	1	4	336	85.8	134.1	4.25	1.5	1	1	5	forage	fish	0	1	chase.cap.hand	successful	ascent	Night	false.pos
W1859	surge.x	testing.generic	0.1	5	1	5	336	85.8	142.4	11.85	3.05	4	1	5	forage	fish	0	1	chase.cap.hand	successful	ascent	Night	false.pos
W1859	sway.y	testing.generic	0.1	5	1	0	2	NA	NA	NA	NA	NA	NA	NA	forage	stingray	1	0	chase.cap	successful	ascent	Day	false.neg
W1859	sway.y	testing.generic	0.1	5	1	1	157	83.3	47.6	4.23	1.5	1	3	7	forage	fish	1	1	chase.cap.hand	successful	benthic	Night	true.pos
W1859	sway.y	testing.generic	0.1	5	1	2	157	83.3	53.55	28.24	5.65	3	3	7	forage	fish	0	1	chase.cap.hand	successful	benthic	Night	false.pos
W1859	sway.y	testing.generic	0.1	5	1	3	157	83.3	65.05	15.78	4.75	2	3	7	forage	fish	0	1	chase.cap.hand	successful	benthic	Night	false.pos
W1859	sway.y	testing.generic	0.1	5	2	4	157	83.3	71.8	50.47	11.25	1	3	7	forage	fish	1	1	chase.cap	successful	benthic	Night	true.pos
W1859	sway.y	testing.generic	0.1	5	2	5	157	83.3	79.25	44.07	5	1	3	7	forage	fish	0	1	chase.cap	successful	benthic	Night	false.pos
W1859	sway.y	testing.generic	0.1	5	2	6	157	83.3	88	4.23	1.5	6	3	7	forage	fish	0	1	chase.cap	successful	benthic	Night	false.pos
W1859	sway.y	testing.generic	0.1	5	3	7	157	83.3	119.7	28.24	5.65	1	3	7	forage	unknown	1	1	chase.cap.hand	successful	ascent	Night	true.pos
W1859	sway.y	testing.generic	0.1	5	1	1	167	83.3	59.8	22.85	6	4	3	4	forage	fish	1	1	chase.cap.hand	successful	benthic	Night	true.pos
W1859	sway.y	testing.generic	0.1	5	1	2	167	83.3	76.15	44.86	8.85	4	3	4	forage	fish	0	1	chase.cap.hand	successful	benthic	Night	false.pos
W1859	sway.y	testing.generic	0.1	5	2	3	167	83.3	89.1	12.88	4.15	3	3	4	forage	fish	1	1	cap.hand	successful	benthic	Night	true.pos
W1859	sway.y	testing.generic	0.1	5	3	4	167	83.3	115.5	27.18	4.25	5	3	4	forage	fish	1	1	chase.cap.hand	successful	ascent	Night	true.pos
W1859	sway.y	testing.generic	0.1	5	1	1	168	83.3	50.1	1.85	0.9	1	2	5	forage	fish	1	1	chase.cap.hand	successful	benthic	Night	true.pos
W1859	sway.y	testing.generic	0.1	5	1	2	168	83.3	86.45	9.37	2.2	2	2	5	forage	fish	0	1	chase.cap.hand	successful	benthic	Night	false.pos
W1859	sway.y	testing.generic	0.1	5	1	3	168	83.3	99.15	35.76	8.2	5	2	5	forage	fish	0	1	chase.cap.hand	successful	benthic	Night	false.pos
W1859	sway.y	testing.generic	0.1	5	2	4	168	83.3	117.6	11.62	3	1	2	5	forage	fish	1	1	chase.cap.hand	successful	ascent	Night	true.pos
W1859	sway.y	testing.generic	0.1	5	2	5	168	83.3	140.8	1.85	0.9	1	2	5	forage	fish	0	1	chase.cap.hand	successful	ascent	Night	false.pos
W1859	sway.y	testing.generic	0.1	5	1	1	169	83.8	50.65	39.11	6.55	4	2	4	forage	fish	1	1	chase.cap.hand	successful	benthic	Night	true.pos
W1859	sway.y	testing.generic	0.1	5	1	2	169	83.8	67.3	48.47	5.9	1	2	4	forage	fish	0	1	chase.cap.hand	successful	benthic	Night	false.pos
W1859	sway.y	testing.generic	0.1	5	1	3	169	83.8	72.9	39.11	6.55	1	2	4	forage	fish	0	1	chase.cap.hand	successful	benthic	Night	false.pos
W1859	sway.y	testing.generic	0.1	5	2	4	169	83.8	129.65	48.47	5.9	2	2	4	forage	fish	1	1	chase.cap.hand	successful	ascent	Night	true.pos
W1859	sway.y	testing.generic	0.1	5	1	1	172	83.8	78.25	41.89	6.15	2	2	2	forage	fish	1	1	chase(miss)	unsuccessful	benthic	Night	true.pos
W1859	sway.y	testing.generic	0.1	5	2	2	172	83.8	127.35	26.92	5.2	3	2	2	forage	fish	1	1	chase.cap.hand	successful	ascent	Night	true.pos
W1859	sway.y	testing.generic	0.1	5	1	1	174	83.8	83.6	29.62	4.4	1	2	3	forage	fish	1	1	chase.cap.hand	successful	benthic	Night	true.pos
W1859	sway.y	testing.generic	0.1	5	1	2	174	83.8	88.8	24.47	4.3	1	2	3	forage	fish	0	1	chase.cap.hand	successful	benthic	Night	false.pos
W1859	sway.y	testing.generic	0.1	5	2	3	174	83.8	118.95	29.62	4.4	3	2	3	forage	unknown	1	1	chase(miss)	unsuccessful	benthic	Night	true.pos
W1859	sway.y	testing.generic	0.1	5	1	1	261	84.5	119.85	10.65	1.55	1	1	2	forage	fish	1	1	cap.hand	successful	ascent	Day	true.pos
W1859	sway.y	testing.generic	0.1	5	1	2	261	84.5	130	7.59	2.75	2	1	2	forage	fish	0	1	cap.hand	successful	ascent	Day	false.pos
W1859	sway.y	testing.generic	0.1	5	1	1	263	84.5	65	14.19	3.1	2	2	5	forage	fish	1	1	chase.cap.hand	successful	benthic	Day	true.pos
W1859	sway.y	testing.generic	0.1	5	1	2	263	84.5	72.9	26.48	6.55	4	2	5	forage	fish	0	1	chase.cap.hand	successful	benthic	Day	false.pos
W1859	sway.y	testing.generic	0.1	5	2	3	263	84.5	102.6	4.03	1.5	1	2	5	forage	fish	1	1	chase.cap.hand	successful	ascent	Day	true.pos
W1859	sway.y	testing.generic	0.1	5	2	4	263	84.5	108.1	14.19	3.1	1	2	5	forage	fish	0	1	chase.cap.hand	successful	ascent	Day	false.pos
W1859	sway.y	testing.generic	0.1	5	2	5	263	84.5	113.15	26.48	6.55	1	2	5	forage	fish	0	1	chase.cap.hand	successful	ascent	Day	false.pos
W1859	sway.y	testing.generic	0.1	5	0	1	266	84.5	54.25	52.52	8.85	4	1	2	forage	no prey	0	1	no prey	no prey	no prey	Day	false.pos
W1859	sway.y	testing.generic	0.1	5	1	2	266	84.5	86.5	3.96	1.75	2	1	2	forage	fish	1	1	chase.cap	successful	ascent	Day	true.pos
W1859	sway.y	testing.generic	0.1	5	1	1	267	84.5	94.7	17.62	4	2	1	1	forage	fish	1	1	chase.cap.hand	successful	ascent	Day	true.pos
W1859	sway.y	testing.generic	0.1	5	0	1	269	84.5	80.75	1.49	0.65	1	0	1	control	no prey	0	1	no prey	no prey	no prey	Day	false.pos
W1859	sway.y	testing.generic	0.1	5	1	1	329	85.8	52.45	29.88	5.15	2	4	8	forage	fish	1	1	chase.cap.hand	successful	benthic	Night	true.pos
W1859	sway.y	testing.generic	0.1	5	1	2	329	85.8	61.9	22.49	2.65	1	4	8	forage	fish	0	1	chase.cap.hand	successful	benthic	Night	false.pos
W1859	sway.y	testing.generic	0.1	5	2	3	329	85.8	70.4	15.59	2.8	1	4	8	forage	fish	1	1	cap.hand	successful	benthic	Night	true.pos
W1859	sway.y	testing.generic	0.1	5	2	4	329	85.8	79.25	29.88	5.15	4	4	8	forage	fish	0	1	cap.hand	successful	benthic	Night	false.pos
W1859	sway.y	testing.generic	0.1	5	4	5	329	85.8	118.2	22.49	2.65	1	4	8	forage	fish	1	1	chase.cap	successful	ascent	Night	true.pos
W1859	sway.y	testing.generic	0.1	5	4	6	329	85.8	128.65	15.59	2.8	1	4	8	forage	fish	0	1	chase.cap	successful	ascent	Night	false.pos
W1859	sway.y	testing.generic	0.1	5	4	7	329	85.8	139.6	29.88	5.15	1	4	8	forage	fish	0	1	chase.cap	successful	ascent	Night	false.pos
W1859	sway.y	testing.generic	0.1	5	4	8	329	85.8	147.1	22.49	2.65	1	4	8	forage	fish	0	1	chase.cap	successful	ascent	Night	false.pos
W1859	sway.y	testing.generic	0.1	5	3	0	329	NA	NA	NA	NA	NA	NA	NA	forage	fish	1	0	cap.hand	successful	benthic	Night	false.neg
W1859	sway.y	testing.generic	0.1	5	1	1	335	85.8	71.25	4.99	1.45	1	1	2	forage	fish	1	1	chase.cap.hand	successful	ascent	Night	true.pos
W1859	sway.y	testing.generic	0.1	5	1	2	335	85.8	79.65	20.46	4.65	3	1	2	forage	fish	0	1	chase.cap.hand	successful	ascent	Night	false.pos
W1859	sway.y	testing.generic	0.1	5	1	1	336	85.8	54.7	1.02	0.4	1	1	11	forage	fish	1	1	chase.cap.hand	successful	ascent	Night	true.pos
W1859	sway.y	testing.generic	0.1	5	1	2	336	85.8	68.75	1.88	0.8	1	1	11	forage	fish	0	1	chase.cap.hand	successful	ascent	Night	false.pos
W1859	sway.y	testing.generic	0.1	5	1	3	336	85.8	76.4	29.68	4.6	2	1	11	forage	fish	0	1	chase.cap.hand	successful	ascent	Night	false.pos
W1859	sway.y	testing.generic	0.1	5	1	4	336	85.8	83.25	4.59	1.8	2	1	11	forage	fish	0	1	chase.cap.hand	successful	ascent	Night	false.pos
W1859	sway.y	testing.generic	0.1	5	1	5	336	85.8	94.15	13.8	3.15	2	1	11	forage	fish	0	1	chase.cap.hand	successful	ascent	Night	false.pos
W1859	sway.y	testing.generic	0.1	5	1	6	336	85.8	103.9	6.34	2.15	2	1	11	forage	fish	0	1	chase.cap.hand	successful	ascent	Night	false.pos
W1859	sway.y	testing.generic	0.1	5	1	7	336	85.8	117.45	17.8	4.85	1	1	11	forage	fish	0	1	chase.cap.hand	successful	ascent	Night	false.pos
W1859	sway.y	testing.generic	0.1	5	1	8	336	85.8	123.85	77.26	7.6	1	1	11	forage	fish	0	1	chase.cap.hand	successful	ascent	Night	false.pos
W1859	sway.y	testing.generic	0.1	5	1	9	336	85.8	130.05	1.02	0.4	3	1	11	forage	fish	0	1	chase.cap.hand	successful	ascent	Night	false.pos
W1859	sway.y	testing.generic	0.1	5	1	10	336	85.8	141.8	1.88	0.8	4	1	11	forage	fish	0	1	chase.cap.hand	successful	ascent	Night	false.pos
W1859	sway.y	testing.generic	0.1	5	1	11	336	85.8	151.95	29.68	4.6	3	1	11	forage	fish	0	1	chase.cap.hand	successful	ascent	Night	false.pos
W1859	heave.z	testing.generic	0.1	5	1	0	2	NA	NA	NA	NA	NA	NA	NA	forage	stingray	1	0	chase.cap	successful	ascent	Day	false.neg
W1859	heave.z	testing.generic	0.1	5	1	1	157	83.3	55.85	4.99	1.6	1	3	4	forage	fish	1	1	chase.cap.hand	successful	benthic	Night	true.pos
W1859	heave.z	testing.generic	0.1	5	1	2	157	83.3	61	5.5	1.5	1	3	4	forage	fish	0	1	chase.cap.hand	successful	benthic	Night	false.pos
W1859	heave.z	testing.generic	0.1	5	2	3	157	83.3	94.05	8.73	2.45	1	3	4	forage	fish	1	1	chase.cap	successful	benthic	Night	true.pos
W1859	heave.z	testing.generic	0.1	5	3	4	157	83.3	119.65	8.6	1.75	1	3	4	forage	unknown	1	1	chase.cap.hand	successful	ascent	Night	true.pos
W1859	heave.z	testing.generic	0.1	5	1	1	167	83.3	76.1	4.15	1.4	1	3	1	forage	fish	1	1	chase.cap.hand	successful	benthic	Night	true.pos
W1859	heave.z	testing.generic	0.1	5	2	0	167	NA	NA	NA	NA	NA	NA	NA	forage	fish	1	0	cap.hand	successful	benthic	Night	false.neg
W1859	heave.z	testing.generic	0.1	5	3	0	167	NA	NA	NA	NA	NA	NA	NA	forage	fish	1	0	chase.cap.hand	successful	ascent	Night	false.neg
W1859	heave.z	testing.generic	0.1	5	1	1	168	83.3	103.4	2.66	1	1	2	1	forage	fish	1	1	chase.cap.hand	successful	benthic	Night	true.pos
W1859	heave.z	testing.generic	0.1	5	2	0	168	NA	NA	NA	NA	NA	NA	NA	forage	fish	1	0	chase.cap.hand	successful	ascent	Night	false.neg
W1859	heave.z	testing.generic	0.1	5	1	1	169	83.8	58.05	3.08	1.3	2	2	2	forage	fish	1	1	chase.cap.hand	successful	benthic	Night	true.pos
W1859	heave.z	testing.generic	0.1	5	2	2	169	83.8	129.5	4.25	1.9	2	2	2	forage	fish	1	1	chase.cap.hand	successful	ascent	Night	true.pos
W1859	heave.z	testing.generic	0.1	5	1	1	172	83.8	78.3	12.19	1.7	2	2	1	forage	fish	1	1	chase(miss)	unsuccessful	benthic	Night	true.pos
W1859	heave.z	testing.generic	0.1	5	2	0	172	NA	NA	NA	NA	NA	NA	NA	forage	fish	1	0	chase.cap.hand	successful	ascent	Night	false.neg
W1859	heave.z	testing.generic	0.1	5	1	1	174	83.8	82.45	3.1	1.45	1	2	2	forage	fish	1	1	chase.cap.hand	successful	benthic	Night	true.pos
W1859	heave.z	testing.generic	0.1	5	2	2	174	83.8	119	3.32	1.35	1	2	2	forage	unknown	1	1	chase(miss)	unsuccessful	benthic	Night	true.pos
W1859	heave.z	testing.generic	0.1	5	1	1	261	84.5	119.7	8.75	1.5	1	1	1	forage	fish	1	1	cap.hand	successful	ascent	Day	true.pos
W1859	heave.z	testing.generic	0.1	5	1	1	263	84.5	64.95	9.96	3	2	2	3	forage	fish	1	1	chase.cap.hand	successful	benthic	Day	true.pos
W1859	heave.z	testing.generic	0.1	5	1	2	263	84.5	77.8	7.01	1.5	1	2	3	forage	fish	0	1	chase.cap.hand	successful	benthic	Day	false.pos
W1859	heave.z	testing.generic	0.1	5	2	3	263	84.5	107.85	9.96	3	1	2	3	forage	fish	1	1	chase.cap.hand	successful	ascent	Day	true.pos
W1859	heave.z	testing.generic	0.1	5	0	1	266	84.5	54.2	17.79	5.65	4	1	1	forage	no prey	0	1	no prey	no prey	no prey	Day	false.pos
W1859	heave.z	testing.generic	0.1	5	1	0	266	NA	NA	NA	NA	NA	NA	NA	forage	fish	1	0	chase.cap	successful	ascent	Day	false.neg
W1859	heave.z	testing.generic	0.1	5	1	1	267	84.5	94.4	9.36	3	2	1	1	forage	fish	1	1	chase.cap.hand	successful	ascent	Day	true.pos
W1859	heave.z	testing.generic	0.1	5	0	0	269	NA	NA	NA	NA	NA	NA	NA	control	no prey	0	0	no prey	no prey	no prey	Day	true.neg
W1859	heave.z	testing.generic	0.1	5	2	1	329	85.8	70.3	0.85	0.4	1	4	3	forage	fish	1	1	cap.hand	successful	benthic	Night	true.pos
W1859	heave.z	testing.generic	0.1	5	2	2	329	85.8	83.1	12	1.55	1	4	3	forage	fish	0	1	cap.hand	successful	benthic	Night	false.pos
W1859	heave.z	testing.generic	0.1	5	4	3	329	85.8	146.6	5.44	1.5	1	4	3	forage	fish	1	1	chase.cap	successful	ascent	Night	true.pos
W1859	heave.z	testing.generic	0.1	5	1	0	329	NA	NA	NA	NA	NA	NA	NA	forage	fish	1	0	chase.cap.hand	successful	benthic	Night	false.neg
W1859	heave.z	testing.generic	0.1	5	3	0	329	NA	NA	NA	NA	NA	NA	NA	forage	fish	1	0	cap.hand	successful	benthic	Night	false.neg
W1859	heave.z	testing.generic	0.1	5	1	1	335	85.8	79.6	4.47	1.4	1	1	1	forage	fish	1	1	chase.cap.hand	successful	ascent	Night	true.pos
W1859	heave.z	testing.generic	0.1	5	1	1	336	85.8	83.55	1.93	0.8	1	1	2	forage	fish	1	1	chase.cap.hand	successful	ascent	Night	true.pos
W1859	heave.z	testing.generic	0.1	5	1	2	336	85.8	151.95	25.03	4.15	2	1	2	forage	fish	0	1	chase.cap.hand	successful	ascent	Night	false.pos
W1873	surge.x	testing.generic	0.1	5	0	0	27	NA	NA	NA	NA	NA	NA	NA	control	no prey	0	0	no prey	no prey	no prey	Night	true.neg
W1873	surge.x	testing.generic	0.1	5	0	1	28	80.5	81.1	1.56	0.75	1	0	1	control	no prey	0	1	no prey	no prey	no prey	Day	false.pos
W1873	surge.x	testing.generic	0.1	5	0	0	29	NA	NA	NA	NA	NA	NA	NA	control	no prey	0	0	no prey	no prey	no prey	Day	true.neg
W1873	surge.x	testing.generic	0.1	5	0	0	31	NA	NA	NA	NA	NA	NA	NA	control	no prey	0	0	no prey	no prey	no prey	Day	true.neg
W1873	surge.x	testing.generic	0.1	5	1	1	58	81	106.65	5.46	1.5	1	1	2	forage	fish	1	1	chase.cap.hand	successful	ascent	Day	true.pos
W1873	surge.x	testing.generic	0.1	5	1	2	58	81	112.1	24.95	6.9	2	1	2	forage	fish	0	1	chase.cap.hand	successful	ascent	Day	false.pos
W1873	surge.x	testing.generic	0.1	5	0	0	59	NA	NA	NA	NA	NA	NA	NA	control	no prey	0	0	no prey	no prey	no prey	Day	true.neg
W1873	surge.x	testing.generic	0.1	5	0	0	86	NA	NA	NA	NA	NA	NA	NA	control	no prey	0	0	no prey	no prey	no prey	Day	true.neg
W1873	surge.x	testing.generic	0.1	5	1	1	107	83.4	68.6	44.27	7.5	2	4	6	forage	fish	1	1	chase.cap.hand	successful	benthic	Night	true.pos
W1873	surge.x	testing.generic	0.1	5	1	2	107	83.4	78.05	53.22	9	4	4	6	forage	fish	0	1	chase.cap.hand	successful	benthic	Night	false.pos
W1873	surge.x	testing.generic	0.1	5	2	3	107	83.4	168.2	42.59	5.1	1	4	6	forage	fish	1	1	chase.cap.hand	successful	benthic	Night	true.pos
W1873	surge.x	testing.generic	0.1	5	3	4	107	83.4	174.55	21	4.05	1	4	6	forage	fish	1	1	chase.cap.hand	successful	benthic	Night	true.pos
W1873	surge.x	testing.generic	0.1	5	3	5	107	83.4	184.55	44.27	7.5	2	4	6	forage	fish	0	1	chase.cap.hand	successful	benthic	Night	false.pos
W1873	surge.x	testing.generic	0.1	5	4	6	107	83.4	263.4	53.22	9	2	4	6	forage	fish	1	1	chase.cap.hand	successful	ascent	Night	true.pos
W1873	surge.x	testing.generic	0.1	5	0	1	112	83.4	180.95	82.28	7.85	4	0	1	control	no prey	0	1	no prey	no prey	no prey	Night	false.pos
W1873	surge.x	testing.generic	0.1	5	1	1	113	83.9	61.3	113.19	15.4	5	4	5	forage	fish	1	1	chase.cap.hand	successful	benthic	Night	true.pos

W1873	surge.x	testing.generic	0.1	5	2	2	113	83.9	100.4	46.56	5.4	1	4	5	forage	fish	1	1	chase.cap.hand	successful	benthic	Night	true.pos
W1873	surge.x	testing.generic	0.1	5	2	3	113	83.9	116.05	113.19	15.4	1	4	5	forage	fish	0	1	chase.cap.hand	successful	benthic	Night	false.pos
W1873	surge.x	testing.generic	0.1	5	2	4	113	83.9	147.3	46.56	5.4	1	4	5	forage	fish	0	1	chase.cap.hand	successful	benthic	Night	false.pos
W1873	surge.x	testing.generic	0.1	5	4	5	113	83.9	163.65	113.19	15.4	1	4	5	forage	fish	1	1	chase.cap	successful	ascent	Night	true.pos
W1873	surge.x	testing.generic	0.1	5	3	0	113	NA	NA	NA	NA	NA	NA	NA	forage	fish	1	0	cap.hand	successful	benthic	Night	false.neg
W1873	surge.x	testing.generic	0.1	5	1	1	130	84.9	52.9	18.33	5	2	2	2	forage	fish	1	1	chase.cap.hand	successful	benthic	Night	true.pos
W1873	surge.x	testing.generic	0.1	5	2	2	130	84.9	111.4	51.11	7.55	4	2	2	forage	fish	1	1	chase.cap.hand	successful	benthic	Night	true.pos
W1873	surge.x	testing.generic	0.1	5	1	1	131	85.4	61.3	25.88	4.6	1	7	11	forage	unknown	1	1	chase.cap.hand	successful	benthic	Night	true.pos
W1873	surge.x	testing.generic	0.1	5	2	2	131	85.4	103.8	25.92	7.1	4	7	11	forage	fish	1	1	cap.hand	successful	benthic	Night	true.pos
W1873	surge.x	testing.generic	0.1	5	3	3	131	85.4	117.5	46.67	6.45	2	7	11	forage	fish	1	1	chase.cap.hand	successful	benthic	Night	true.pos
W1873	surge.x	testing.generic	0.1	5	4	4	131	85.4	126.45	20.51	4.1	1	7	11	forage	fish	1	1	chase.cap.hand	successful	benthic	Night	true.pos
W1873	surge.x	testing.generic	0.1	5	4	5	131	85.4	131.65	33.49	6.15	1	7	11	forage	fish	0	1	chase.cap.hand	successful	benthic	Night	false.pos
W1873	surge.x	testing.generic	0.1	5	5	6	131	85.4	148.95	1.35	0.65	1	7	11	forage	fish	1	1	chase.cap.hand	successful	benthic	Night	true.pos
W1873	surge.x	testing.generic	0.1	5	5	7	131	85.4	156.1	25.88	4.6	4	7	11	forage	fish	0	1	chase.cap.hand	successful	benthic	Night	false.pos
W1873	surge.x	testing.generic	0.1	5	6	8	131	85.4	169.95	25.92	7.1	1	7	11	forage	fish	1	1	chase.cap.hand	successful	benthic	Night	true.pos
W1873	surge.x	testing.generic	0.1	5	6	9	131	85.4	177.75	46.67	6.45	4	7	11	forage	fish	0	1	chase.cap.hand	successful	benthic	Night	false.pos
W1873	surge.x	testing.generic	0.1	5	7	10	131	85.4	207.35	20.51	4.1	1	7	11	forage	fish	1	1	chase.cap	successful	ascent	Night	true.pos
W1873	surge.x	testing.generic	0.1	5	0	11	131	85.4	252.35	33.49	6.15	1	7	11	forage	no prey	0	1	no prey	no prey	no prey	Night	false.pos
W1873	surge.x	testing.generic	0.1	5	1	1	132	84.9	102	50.02	6	2	5	7	forage	unknown	1	1	chase.cap.hand	successful	benthic	Night	true.pos
W1873	surge.x	testing.generic	0.1	5	2	2	132	84.9	111.9	28.94	5.5	1	5	7	forage	fish	1	1	chase.cap.hand	successful	benthic	Night	true.pos
W1873	surge.x	testing.generic	0.1	5	2	3	132	84.9	120.05	50.02	6	1	5	7	forage	fish	0	1	chase.cap.hand	successful	benthic	Night	false.pos
W1873	surge.x	testing.generic	0.1	5	3	4	132	84.9	127.65	28.94	5.5	1	5	7	forage	fish	1	1	chase.cap.hand	successful	benthic	Night	true.pos
W1873	surge.x	testing.generic	0.1	5	4	5	132	84.9	136.5	50.02	6	1	5	7	forage	unknown	1	1	chase.cap.hand	successful	benthic	Night	true.pos
W1873	surge.x	testing.generic	0.1	5	5	6	132	84.9	146.35	28.94	5.5	1	5	7	forage	unknown	1	1	cap.hand	successful	benthic	Night	true.pos
W1873	surge.x	testing.generic	0.1	5	0	7	132	84.9	198.05	50.02	6	3	5	7	forage	no prey	0	1	no prey	no prey	no prey	Night	false.pos
W1873	surge.x	testing.generic	0.1	5	1	1	133	84.9	57.3	12.4	3.2	1	4	5	forage	fish	1	1	chase.cap.hand	successful	benthic	Night	true.pos
W1873	surge.x	testing.generic	0.1	5	2	2	133	84.9	102.65	16.1	3.55	1	4	5	forage	fish	1	1	chase.cap.hand	successful	benthic	Night	true.pos
W1873	surge.x	testing.generic	0.1	5	2	3	133	84.9	108.8	15.33	3.45	2	4	5	forage	fish	0	1	chase.cap.hand	successful	benthic	Night	false.pos
W1873	surge.x	testing.generic	0.1	5	3	4	133	84.9	131.35	24.07	4.5	2	4	5	forage	fish	1	1	cap.hand	successful	benthic	Night	true.pos
W1873	surge.x	testing.generic	0.1	5	4	5	133	84.9	149.3	12.4	3.2	3	4	5	forage	fish	1	1	chase.cap.hand	successful	ascent	Night	true.pos
W1873	surge.x	testing.generic	0.1	5	1	1	136	85.4	61.35	58.74	7.75	4	5	6	forage	fish	1	1	chase.cap.hand	successful	benthic	Night	true.pos
W1873	surge.x	testing.generic	0.1	5	2	2	136	85.4	103.7	18.7	4.5	2	5	6	forage	fish	1	1	chase.cap.hand	successful	benthic	Night	true.pos
W1873	surge.x	testing.generic	0.1	5	3	3	136	85.4	111.9	10.18	3	2	5	6	forage	fish	1	1	chase.cap.hand	successful	benthic	Night	true.pos
W1873	surge.x	testing.generic	0.1	5	3	4	136	85.4	120.5	51.1	7.6	2	5	6	forage	fish	0	1	chase.cap.hand	successful	benthic	Night	false.pos
W1873	surge.x	testing.generic	0.1	5	4	5	136	85.4	158.65	34.1	4.8	2	5	6	forage	fish	1	1	chase.cap.hand	successful	benthic	Night	true.pos
W1873	surge.x	testing.generic	0.1	5	5	6	136	85.4	191.6	25.97	4.7	3	5	6	forage	fish	1	1	chase(miss)	unsuccessful	benthic	Night	true.pos
W1873	surge.x	testing.generic	0.1	5	1	1	138	85.4	104.75	15.61	3.35	2	4	5	forage	unknown	1	1	chase.cap.hand	successful	benthic	Night	true.pos
W1873	surge.x	testing.generic	0.1	5	2	2	138	85.4	123.95	26.35	3.35	1	4	5	forage	fish	1	1	chase.cap.hand	successful	benthic	Night	true.pos
W1873	surge.x	testing.generic	0.1	5	3	3	138	85.4	134.5	17.76	1.65	2	4	5	forage	unknown	1	1	cap.hand	successful	benthic	Night	true.pos
W1873	surge.x	testing.generic	0.1	5	4	4	138	85.4	180.55	15.61	3.35	1	4	5	forage	fish	1	1	chase.cap	successful	ascent	Night	true.pos
W1873	surge.x	testing.generic	0.1	5	4	5	138	85.4	187.15	26.35	3.35	1	4	5	forage	fish	0	1	chase.cap	successful	ascent	Night	false.pos
W1873	surge.x	testing.generic	0.1	5	1	1	181	85.4	164.8	26.46	4.25	4	1	3	forage	fish	1	1	chase.cap.hand	successful	ascent	Day	true.pos
W1873	surge.x	testing.generic	0.1	5	1	2	181	85.4	179.2	0.73	0.2	1	1	3	forage	fish	0	1	chase.cap.hand	successful	ascent	Day	false.pos
W1873	surge.x	testing.generic	0.1	5	0	3	181	85.4	229.55	26.46	4.25	1	1	3	forage	no prey	0	1	no prey	no prey	no prey	Day	false.pos
W1873	surge.x	testing.generic	0.1	5	1	0	182	NA	NA	NA	NA	NA	NA	NA	forage	unknown	1	0	cap.hand	successful	ascent	Day	false.neg
W1873	surge.x	testing.generic	0.1	5	0	0	183	NA	NA	NA	NA	NA	NA	NA	control	no prey	0	0	no prey	no prey	no prey	Day	true.neg
W1873	surge.x	testing.generic	0.1	5	0	0	184	NA	NA	NA	NA	NA	NA	NA	control	no prey	0	0	no prey	no prey	no prey	Day	true.neg
W1873	surge.x	testing.generic	0.1	5	0	0	225	NA	NA	NA	NA	NA	NA	NA	control	no prey	0	0	no prey	no prey	no prey	Day	true.neg
W1873	surge.x	testing.generic	0.1	5	0	0	226	NA	NA	NA	NA	NA	NA	NA	control	no prey	0	0	no prey	no prey	no prey	Day	true.neg
W1873	surge.x	testing.generic	0.1	5	0	0	228	NA	NA	NA	NA	NA	NA	NA	control	no prey	0	0	no prey	no prey	no prey	Day	true.neg
W1873	surge.x	testing.generic	0.1	5	1	1	232	84.4	143.45	12.16	1.9	1	1	1	forage	unknown	1	1	chase.cap.hand	successful	benthic	Day	true.pos
W1873	surge.x	testing.generic	0.1	5	0	0	233	NA	NA	NA	NA	NA	NA	NA	control	no prey	0	0	no prey	no prey	no prey	Day	true.neg
W1873	surge.x	testing.generic	0.1	5	1	1	235	84.4	109.6	32.33	4.35	2	1	1	forage	unknown	1	1	chase.cap.hand	successful	benthic	Day	true.pos
W1873	surge.x	testing.generic	0.1	5	1	1	236	84.4	87.3	3.14	1.4	1	1	1	forage	unknown	1	1	cap.hand	successful	benthic	Day	true.pos
W1873	surge.x	testing.generic	0.1	5	1	1	269	85.3	63.35	26.63	5.15	1	1	2	forage	fish	1	1	chase.cap.hand	successful	benthic	Night	true.pos
W1873	surge.x	testing.generic	0.1	5	1	2	269	85.3	68.85	4.77	1.55	1	1	2	forage	fish	0	1	chase.cap.hand	successful	benthic	Night	false.pos
W1873	surge.x	testing.generic	0.1	5	1	1	271	83.8	81.9	13.27	1.75	1	2	3	forage	fish	1	1	chase.cap.hand	successful	benthic	Night	true.pos
W1873	surge.x	testing.generic	0.1	5	1	2	271	83.8	86.95	36.35	6.65	2	2	3	forage	fish	0	1	chase.cap.hand	successful	benthic	Night	false.pos
W1873	surge.x	testing.generic	0.1	5	2	3	271	83.8	119.1	9.31	1.55	1	2	3	forage	fish	1	1	chase.cap.hand	successful	benthic	Night	true.pos
W1873	surge.x	testing.generic	0.1	5	0	1	272	83.8	60.3	5.12	1.5	1	1	2	forage	no prey	0	1	no prey	no prey	no prey	Night	false.pos
W1873	surge.x	testing.generic	0.1	5	1	2	272	83.8	141.85	71.88	8.8	3	1	2	forage	fish	1	1	chase.cap.hand	successful	ascent	Night	true.pos
W1873	surge.x	testing.generic	0.1	5	1	1	273	83.8	67.3	29.64	4.7	1	3	5	forage	unknown	1	1	chase.cap.hand	successful	benthic	Night	true.pos
W1873	surge.x	testing.generic	0.1	5	1	2	273	83.8	92.3	16.81	3.85	1	3	5	forage	unknown	0	1	chase.cap.hand	successful	benthic	Night	false.pos
W1873	surge.x	testing.generic	0.1	5	2	3	273	83.8	125.75	47.96	3.2	1	3	5	forage	fish	1	1	chase.cap.hand	successful	benthic	Night	true.pos
W1873	surge.x	testing.generic	0.1	5	2	4	273	83.8	131.1	38.72	6.2	1	3	5	forage	fish	0	1	chase.cap.hand	successful	benthic	Night	false.pos
W1873	surge.x	testing.generic	0.1	5	3	5	273	83.8	220.95	10.76	1.6	1	3	5	forage	fish	1	1	chase.cap.hand	successful	ascent	Night	true.pos
W1873	surge.x	testing.generic	0.1	5	1	1	280	84.3	67.05	39.21	6.9	4	3	3	forage	fish	1	1	chase.cap.hand	successful	benthic	Night	true.pos
W1873	surge.x	testing.generic	0.1	5	2	2	280	84.3	108.7	30.26	5.7	2	3	3	forage	fish	1	1	chase.cap.hand	successful	benthic	Night	true.pos
W1873	surge.x	testing.generic	0.1	5	3	3	280	84.3	147.75	55.4	5.95	2	3	3	forage	fish	1	1	chase.cap.hand	successful	benthic	Night	true.pos
W1873	surge.x	testing.generic	0.1	5	1	1	290	84.8	71.55	59.8	7.8	2	2	2	forage	fish	1	1	chase.cap.hand	successful	benthic	Night	true.pos
W1873	surge.x	testing.generic	0.1	5	2	2	290	84.8	141.8	24.42	8.15	5	2	2	forage	fish	1	1	chase.cap.hand	successful	ascent	Night	true.pos
W1873	surge.x	testing.generic	0.1	5	1	1	294	84.8	61.45	49.45	5.95	2	3	6	forage	fish	1	1	chase.cap.hand	successful	benthic	Night	true.pos
W1873	surge.x	testing.generic	0.1	5	2	2	294	84.8	71.35	36.3	6.3	1	3	6	forage	fish	1	1	chase.cap.hand	successful	benthic	Night	true.pos
W1873	surge.x	testing.generic	0.1	5	2	3	294	84.8	78.95	75.3	8.25	3	3	6	forage	fish	0	1	chase.cap.hand	successful	benthic	Night	false.pos
W1873	surge.x	testing.generic	0.1	5	2	4	294	84.8	91.75	2.84	0.6	1	3	6	forage	fish	0	1	chase.cap.hand	successful	benthic	Night	false.pos
W1873	surge.x	testing.generic	0.1	5	3	5	294	84.8	119.2	49.45	5.95	2	3	6	forage	fish	1	1	chase.cap.hand	successful	ascent	Night	true.pos
W1873	surge.x	testing.generic	0.1	5	0	6	294	84.8	169.2	36.3	6.3	1	3	6	forage	no prey	0	1	no prey	no prey	no prey	Night	false.pos
W1873	surge.x	testing.generic	0.1	5	1	1	295	84.8	68.8	40.82	6.95	2	3	4	forage	fish	1	1	chase.cap.hand	successful	benthic	Night	true.pos
W1873	surge.x	testing.generic	0.1	5	2	2	295	84.8	110.4	33.17	6.45	3	3	4	forage	fish	1	1	chase.cap.hand	successful	benthic	Night	true.pos
W1873	surge.x	testing.generic	0.1	5	2	3	295	84.8	120.35	11.19	3	2	3	4	forage	fish	0	1	chase.cap.hand	successful	benthic	Night	false.pos
W1873	surge.x	testing.generic	0.1	5	3	4	295	84.8	164.85	10.31	1.55	1	3	4	forage	fish	1	1	chase.cap	successful	ascent	Night	true.pos
W1873	surge.x	testing.generic	0.1	5	1	1	297	84.8	53.05	24.83	3.95	2	6	6	forage	fish	1	1	chase.cap.hand	successful	benthic	Night	true.pos
W1873	surge.x	testing.generic	0.1	5	2	2	297	84.8	81.35	27.09	3.7	1	6	6	forage	fish	1	1	chase.cap.hand	successful	benthic	Night	true.pos
W1873	surge.x	testing.generic	0.1	5	3	3	297	84.8	89.05	49.93	7.25	2	6	6	forage	fish	1	1	chase.cap.hand	successful	benthic	Night	true.pos
W1873	surge.x	testing.generic	0.1	5	4	4	297	84.8	106.45	28.5	4.4	3	6	6	forage	fish	1	1	chase.cap.hand	successful	benthic	Night	true.pos
W1873	surge.x	testing.generic	0.1	5	5	5	297	84.8	136.65	7.47	1.55	2	6	6	forage	fish	1	1	chase.cap.hand	successful	benthic	Night	true.pos
W1873	surge.x	testing.generic	0.1	5	6	6	297	84.8	160.45	24.83	3.95	1	6	6	forage	fish	1	1	chase.cap	successful	ascent	Night	true.pos
W1873	surge.x	testing.generic	0.1	5	1	1	327	84.3	65.4	10.26	1.55	1	1	1	forage	unknown	1	1	cap.hand	successful	ascent	Day	true.pos
W1873	surge.x	testing.generic	0.1	5	0	0	360	NA	NA	NA	NA	NA	NA	NA	control	no prey	0	0	no prey	no prey	no prey	Day	true.neg
W1873	surge.x	testing.generic	0.1	5	1	1	362	85.2	80.4	49.27	5.6	3	1	1	forage	fish	1	1	chase.cap.hand	successful	ascent	Night	true.pos
W1873	surge.x	testing.generic	0.1	5	1	1	363	85.2	87.85	37	5.25	2	2	2	forage	fish	1	1	chase.cap.hand	successful	benthic	Night	true.pos
W1873	surge.x	testing.generic	0.1	5	2	2	363	85.2	137.3	26.19	2.9	1	2	2	forage	fish	1	1	chase(miss)	unsuccessful	benthic	Night	true.pos
W1873	sway.y	testing.generic	0.1	5	0	0	27	NA	NA	NA	NA	NA	NA	NA	control	no prey	0	0	no prey	no prey	no prey	Night	true.neg
W1873	sway.y	testing.generic	0.1	5	0	1	28	80.5	70.95	3.12	1.4	1	0	1	control	no prey	0	1	no prey	no prey	no prey	Day	false.pos
W1873	sway.y	testing.generic	0.1	5	0	0	29	NA	NA	NA	NA	NA	NA	NA	control	no prey	0	0	no prey	no prey	no prey	Day	true.neg
W1873	sway.y	testing.generic	0.1	5	0	0	31	NA	NA	NA	NA	NA	NA	NA	control	no prey	0	0	no prey	no prey	no prey	Day	true.neg
W1873	sway.y	testing.generic	0.1	5	1	1	58	81	106.6	9.66	1.55	1	1	3	forage	fish	1	1	chase.cap.hand	successful	ascent	Day	true.pos
W1873	sway.y	testing.generic	0.1	5	1	2	58	81	111.8	44.96	6.25	3	1	3	forage	fish	0	1	chase.cap.hand	successful	ascent	Day	false.pos
W1873	sway.y	testing.generic	0.1	5	1	3	58	81	124.75	0.27	0.1	1	1	3	forage	fish	0	1	chase.cap.hand	successful	ascent	Day	false.pos
W1873	sway.y	testing.generic	0.1	5	0	1	59	81.5	141.75	3.46	1.45	1	0	1	control	no prey	0	1	no prey	no prey	no prey	Day	false.pos
W1873	sway.y	testing.generic	0.1	5	0	1	86	82.9	95.3	2.89	1.35	1	0	2	control	no prey	0	1	no prey	no prey	no prey	Night	false.pos
W1873	sway.y	testing.generic	0.1	5	0	2	86	82.9	119.9	3.83	1.45	1	0	2	control	no prey	0	1	no prey	no prey	no prey	Night	false.pos
W1873	sway.y	testing.generic	0.1	5	1	1	107	83.4	45.05	2.79	1.15	2	4	13	forage	fish	1	1	chase.cap.hand	successful	benthic	Night	true.pos
W1873	sway.y	testing.generic	0.1	5	1	2	107	83.4	67.3	57.74	7.3	1	4	13	forage	fish	0	1	chase.cap.hand	successful	benthic	Night	false.pos
W1873	sway.y	testing.generic	0.1	5	1	3	107	83.4	72.85	42.37	8.1	1	4	13	forage	fish	0	1	chase.cap.hand	successful	benthic	Night	false.pos
W1873	sway.y	testing.generic	0.1	5	1	4	107	83.4	78	43.21	5.65	2	4	13	forage	fish	0	1	chase.cap.hand	successful	benthic	Night	false.pos
W1873	sway.y	testing.generic	0.1	5	1	5	107	83.4	85.9	37.92	5.45	1	4	13	forage	fish	0	1	chase.cap.hand	successful	benthic	Night	false.pos
W1873	sway.y	testing.generic	0.1	5	0	6	107	83.4	98.15	2.79	1.15	1	4	13	forage	no prey	0	1	no prey	no prey	no prey	Night	false.pos
W1873	sway.y	testing.generic	0.1	5	0	7	107	83.4	126.05	57.74	7.3	1	4	13	forage	no prey	0	1	no prey	no prey	no prey	Night	false.pos
W1873	sway.y	testing.generic	0.1	5	2	8	107	83.4	151.45	42.37	8.1	1	4	13	forage	fish	1	1	chase.cap.hand	successful	benthic	Night	true.pos
W1873	sway.y	testing.generic	0.1	5	2	9	107	83.4	170.3	43.21	5.65	2	4	13	forage	fish	0	1	chase.cap.hand	successful	benthic	Night	false.pos
W1873	sway.y	testing.generic	0.1	5	3	10	107	83.4	181.4	37.92	5.45	3	4	13	forage	fish	1	1	chase.cap.hand	successful	benthic	Night	true.pos
W1873	sway.y	testing.generic	0.1	5	4	11	107	83.4	246.6	2.79	1.15	1	4	13	forage	fish	1	1	chase.cap.hand	successful	ascent	Night	true.pos
W1873	sway.y	testing.generic	0.1	5	4	12	107	83.4	259.75	57.74	7.3	1	4	13	forage	fish	0	1	chase.cap.hand	successful	ascent	Night	false.pos
W1873	sway.y	testing.generic	0.1	5	4	13	107	83.4	267.3	42.37	8.1	3	4	13	forage	fish	0	1	chase.cap.hand	successful	ascent	Night	false.pos
W1873	sway.y	testing.generic	0.1	5	0	1	112	83.4	74.4	4.97	1.5	1	0	5	control	no prey	0	1	no prey	no prey	no prey	Night	false.pos
W1873	sway.y	testing.generic	0.1	5	0	2	112	83.4	107.5	1.9	0.8	1	0	5	control	no prey	0	1	no prey	no prey	no prey	Night	false.pos
W1873	sway.y	testing.generic	0.1	5	0	3	112	83.4	138.15	101.12	9.35	1	0	5	control	no prey	0	1	no prey	no prey	no prey	Night	false.pos
W1873	sway.y	testing.generic	0.1	5	0	4	112	83.4	146.4	4.97	1.5	2	0	5	control	no prey	0	1	no prey	no prey	no prey	Night	false.pos
W1873	sway.y	testing.generic	0.1	5	0	5	112	83.4	180.95	1.9	0.8	2	0	5	control	no prey	0	1	no prey	no prey	no prey	Night	false.pos
W1873	sway.y	testing.generic	0.1	5	1	1	113	83.9	60.35	145.72	16.35	3	4	6	forage	fish	1	1	chase.cap.hand	successful	benthic	Night	true.pos
W1873	sway.y	testing.generic	0.1	5	2	2	113	83.9	100.55	25.97	4.85	3	4	6	forage	fish	1	1	chase.cap.hand	successful	benthic	Night	true.pos
W1873	sway.y	testing.generic	0.1	5	2	3	113	83.9	116	32	4.75	1	4	6	forage	fish	0	1	chase.cap.hand	successful	benthic	Night	false.pos
W1873	sway.y	testing.generic	0.1	5	2	4	113	83.9	133.65	51.96	6.55	1	4	6	forage	fish	0	1	chase.cap.hand	successful	benthic	Night	false.pos
W1873	sway.y	testing.generic	0.1	5	2	5	113	83.9	147.35	145.72	16.35	2	4	6	forage	fish	0	1	chase.cap.hand	successful	benthic	Night	false.pos
W1873	sway.y	testing.generic	0.1	5	4	6	113	83.9	163.6	25.97	4.85	3	4	6	forage	fish	1	1	chase.cap	successful	ascent	Night	true.pos
W1873	sway.y	testing.generic	0.1	5	3	0	113	NA	NA	NA	NA	NA	NA	NA	forage	fish	1	0	cap.hand	successful	benthic	Night	false.neg
W1873	sway.y	testing.generic	0.1	5	1	1	130	84.9	47.85	17.43	4.7	3	2	4	forage	fish	1	1	chase.cap.hand	successful	benthic	Night	true.pos
W1873	sway.y	testing.generic	0.1	5	1	2	130	84.9	58.35	50.03	8.3	1	2	4	forage	fish	0	1	chase.cap.hand	successful	benthic	Night	false.pos
W1873	sway.y	testing.generic	0.1	5	2	3	130	84.9	111.5	7.75	1.55	3	2	4	forage	fish	1	1	chase.cap.hand	successful	benthic	Night	true.pos
W1873	sway.y	testing.generic	0.1	5	0	4	130	84.9	198.25	17.43	4.7	1	2	4	forage	no prey	0	1	no prey	no prey	no prey	Night	false.pos
W1873	sway.y	testing.generic	0.1	5	1	1	131	85.4	61.35	59.87	5	1	7	10	forage	unknown	1	1	chase.cap.hand	successful	benthic	Night	true.pos
W1873	sway.y	testing.generic	0.1	5	2	2	131	85.4	103.1	83.95	14.95	6	7	10	forage	fish	1	1	cap.hand	successful	benthic	Night	true.pos
W1873	sway.y	testing.generic	0.1	5	4	3	131	85.4	126.5	38.79	6.55	1	7	10	forage	fish	1	1	chase.cap.hand	successful	benthic	Night	true.pos
W1873	sway.y	testing.generic	0.1	5	4	4	131	85.4	131.7	22.12	1.8	1	7	10	forage	fish	0	1	chase.cap.hand	successful	benthic	Night	false.pos
W1873	sway.y	testing.generic	0.1	5	5	5	131	85.4	149.2	59.87	5	1	7	10	forage	fish	1	1	chase.cap.hand	successful	benthic	Night	true.pos
W1873	sway.y	testing.generic	0.1	5	5	6	131	85.4	156.05	83.95	14.95	3	7	10	forage	fish	0	1	chase.cap.hand	successful	benthic	Night	false.pos
W1873	sway.y	testing.generic	0.1	5	6	7	131	85.4	169.95	38.79	6.55	1	7	10	forage	fish	1	1	chase(no cap)	unsuccessful	benthic	Night	true.pos
W1873	sway.y	testing.generic	0.1	5	6	8	131	85.4	176.95	22.12	1.8	1	7	10	forage	fish	0	1	chase.cap.hand	successful	benthic	Night	false.pos
W1873	sway.y	testing.generic	0.1	5	6	9	131	85.4	182.45	59.87	5	1	7	10	forage	fish	0	1	chase.cap.hand	successful	benthic	Night	false.pos
W1873	sway.y	testing.generic	0.1	5	7	10	131	85.4	207.75	83.95	14.95	1	7	10	forage	fish	1	1	chase.cap	successful	ascent	Night	true.pos
W1873	sway.y	testing.generic	0.1	5	3	0	131	NA	NA	NA	NA	NA	NA	NA	forage	fish	1	0	chase.cap.hand	successful	benthic	Night	false.neg
W1873	sway.y	testing.generic	0.1	5	1	1	132	84.9	102.05	58.58	6.8	2	5	7	forage	unknown	1	1	chase.cap.hand	successful	benthic	Night	true.pos
W1873	sway.y	testing.generic	0.1	5	2	2	132	84.9	111.8	39.5	6.15	3	5	7	forage	fish	1	1	chase.cap.hand	successful	benthic	Night	true.pos
W1873	sway.y	testing.generic	0.1	5	3	3	132	84.9	125.7	35.57	6.75	2	5	7	forage	fish	1	1	chase.cap.hand	successful	benthic	Night	true.pos
W1873	sway.y	testing.generic	0.1	5	4	4	132	84.9	136.45	23.36	4	2	5	7	forage	unknown	1	1	chase.cap.hand	successful	benthic	Night	true.pos
W1873	sway.y	testing.generic	0.1	5	5	5	132	84.9	146	107.87	9.75	1	5	7	forage	unknown	1	1	cap.hand	successful	benthic	Night	true.pos
W1873	sway.y	testing.generic	0.1	5	0	6	132	84.9	168.2	58.58	6.8	1	5	7	forage	no prey	0	1	no prey	no prey	no prey	Night	false.pos
W1873	sway.y	testing.generic	0.1	5	0	7	132	84.9	198.1	39.5	6.15	4	5	7	forage	no prey	0	1	no prey	no prey	no prey	Night	false.pos
W1873	sway.y	testing.generic	0.1	5	1	1	133	84.9	56.5	25.16	3.75	1	4	5	forage	fish	1	1	chase.cap.hand	successful	benthic	Night	true.pos
W1873	sway.y	testing.generic	0.1	5	2	2	133	84.9	99.55	58.9	5.25	3	4	5	forage	fish	1	1	chase.cap.hand	successful	benthic	Night	true.pos
W1873	sway.y	testing.generic	0.1	5	2	3	133	84.9	108.8	24.97	5.55	1	4	5	forage	fish	0	1	chase.cap.hand	successful	benthic	Night	false.pos
W1873	sway.y	testing.generic	0.1	5	3	4	133	84.9	132	25.16	3.75	1	4	5	forage	fish	1	1	cap.hand	successful	benthic	Night	true.pos
W1873	sway.y	testing.generic	0.1	5	4	5	133	84.9	149.3	58.9	5.25	2	4	5	forage	fish	1	1	chase.cap.hand	successful	ascent	Night	true.pos
W1873	sway.y	testing.generic	0.1	5	1	1	136	85.4	55.65	5.59	1.55	1	5	8	forage	fish	1	1	chase.cap.hand	successful	benthic	Night	true.pos
W1873	sway.y	testing.generic	0.1	5	1	2	136	85.4	61.85	42.17	7.25	3	5	8	forage	fish	0	1	chase.cap.hand	successful	benthic	Night	false.pos
W1873	sway.y	testing.generic	0.1	5	2	3	136	85.4	103.75	24.41	4.6	2	5	8	forage	fish	1	1	chase.cap.hand	successful	benthic	Night	true.pos

W1873	sway.y	testing.generic	0.1	5	3	4	136	85.4	111.9	19.37	4.45	2	5	8	forage	fish	1	1	chase.cap.hand	successful	benthic	Night	true.pos
W1873	sway.y	testing.generic	0.1	5	3	5	136	85.4	120.7	89.87	9.95	2	5	8	forage	fish	0	1	chase.cap.hand	successful	benthic	Night	false.pos
W1873	sway.y	testing.generic	0.1	5	4	6	136	85.4	160.05	14.13	3.2	1	5	8	forage	fish	1	1	chase.cap.hand	successful	benthic	Night	true.pos
W1873	sway.y	testing.generic	0.1	5	5	7	136	85.4	191.7	5.59	1.55	1	5	8	forage	fish	1	1	chase(miss)	unsuccessful	benthic	Night	true.pos
W1873	sway.y	testing.generic	0.1	5	5	8	136	85.4	196.85	42.17	7.25	2	5	8	forage	fish	0	1	chase(miss)	unsuccessful	benthic	Night	false.pos
W1873	sway.y	testing.generic	0.1	5	1	1	138	85.4	104.8	39.12	4.65	1	4	6	forage	unknown	1	1	chase.cap.hand	successful	benthic	Night	true.pos
W1873	sway.y	testing.generic	0.1	5	2	2	138	85.4	124.15	28.42	4.85	1	4	6	forage	fish	1	1	chase.cap.hand	successful	benthic	Night	true.pos
W1873	sway.y	testing.generic	0.1	5	3	3	138	85.4	134.55	18.17	3.6	1	4	6	forage	unknown	1	1	cap.hand	successful	benthic	Night	true.pos
W1873	sway.y	testing.generic	0.1	5	4	4	138	85.4	148.45	0.51	0.2	1	4	6	forage	fish	1	1	chase.cap	successful	ascent	Night	true.pos
W1873	sway.y	testing.generic	0.1	5	4	5	138	85.4	180.6	37.05	2.65	1	4	6	forage	fish	0	1	chase.cap	successful	ascent	Night	false.pos
W1873	sway.y	testing.generic	0.1	5	4	6	138	85.4	187	17.12	2	1	4	6	forage	fish	0	1	chase.cap	successful	ascent	Night	false.pos
W1873	sway.y	testing.generic	0.1	5	1	1	181	85.4	167.4	38.94	5.7	1	1	3	forage	fish	1	1	chase.cap.hand	successful	ascent	Day	true.pos
W1873	sway.y	testing.generic	0.1	5	1	2	181	85.4	173.6	20.95	3.85	1	1	3	forage	fish	0	1	chase.cap.hand	successful	ascent	Day	false.pos
W1873	sway.y	testing.generic	0.1	5	1	3	181	85.4	178.8	7.66	2.6	1	1	3	forage	fish	0	1	chase.cap.hand	successful	ascent	Day	false.pos
W1873	sway.y	testing.generic	0.1	5	1	1	182	84.9	122.5	1.67	0.75	1	1	1	forage	unknown	1	1	cap.hand	successful	ascent	Day	true.pos
W1873	sway.y	testing.generic	0.1	5	0	1	183	85.4	190.65	3.05	1.25	1	0	1	control	no prey	0	1	no prey	no prey	no prey	Day	false.pos
W1873	sway.y	testing.generic	0.1	5	0	1	184	84.9	71.55	2.23	1	1	0	1	control	no prey	0	1	no prey	no prey	no prey	Day	false.pos
W1873	sway.y	testing.generic	0.1	5	0	0	225	NA	NA	NA	NA	NA	NA	NA	control	no prey	0	0	no prey	no prey	no prey	Day	true.neg
W1873	sway.y	testing.generic	0.1	5	0	1	226	84.4	118.2	1.88	0.9	1	0	3	control	no prey	0	1	no prey	no prey	no prey	Day	false.pos
W1873	sway.y	testing.generic	0.1	5	0	2	226	84.4	151.1	2.11	0.95	1	0	3	control	no prey	0	1	no prey	no prey	no prey	Day	false.pos
W1873	sway.y	testing.generic	0.1	5	0	3	226	84.4	168	3.78	1.5	1	0	3	control	no prey	0	1	no prey	no prey	no prey	Day	false.pos
W1873	sway.y	testing.generic	0.1	5	0	1	228	84.4	213.55	0.88	0.4	1	0	1	control	no prey	0	1	no prey	no prey	no prey	Day	false.pos
W1873	sway.y	testing.generic	0.1	5	1	1	232	84.4	135.1	10.3	1.6	1	1	2	forage	unknown	1	1	chase.cap.hand	successful	benthic	Day	true.pos
W1873	sway.y	testing.generic	0.1	5	1	2	232	84.4	143.4	19.44	4.15	1	1	2	forage	unknown	0	1	chase.cap.hand	successful	benthic	Day	false.pos
W1873	sway.y	testing.generic	0.1	5	0	0	233	NA	NA	NA	NA	NA	NA	NA	control	no prey	0	0	no prey	no prey	no prey	Day	true.neg
W1873	sway.y	testing.generic	0.1	5	1	1	235	84.4	109.45	71.57	7.65	2	1	2	forage	unknown	1	1	chase.cap.hand	successful	benthic	Day	true.pos
W1873	sway.y	testing.generic	0.1	5	0	2	235	84.4	181.6	3.1	1.2	1	1	2	forage	no prey	0	1	no prey	no prey	no prey	Day	false.pos
W1873	sway.y	testing.generic	0.1	5	1	1	236	84.4	88.5	10.8	3	1	1	1	forage	unknown	1	1	cap.hand	successful	benthic	Day	true.pos
W1873	sway.y	testing.generic	0.1	5	1	1	269	85.3	63.25	30.01	5.9	5	1	3	forage	fish	1	1	chase.cap.hand	successful	benthic	Night	true.pos
W1873	sway.y	testing.generic	0.1	5	0	2	269	85.3	100.6	2.52	1	1	1	3	forage	no prey	0	1	no prey	no prey	no prey	Night	false.pos
W1873	sway.y	testing.generic	0.1	5	0	3	269	85.3	151.75	30.01	5.9	1	1	3	forage	no prey	0	1	no prey	no prey	no prey	Night	false.pos
W1873	sway.y	testing.generic	0.1	5	1	1	271	83.8	82.05	58.75	9.25	3	2	3	forage	fish	1	1	chase.cap.hand	successful	benthic	Night	true.pos
W1873	sway.y	testing.generic	0.1	5	2	2	271	83.8	107.4	9.23	1.9	1	2	3	forage	fish	1	1	chase.cap.hand	successful	benthic	Night	true.pos
W1873	sway.y	testing.generic	0.1	5	2	3	271	83.8	119.55	58.75	9.25	1	2	3	forage	fish	0	1	chase.cap.hand	successful	benthic	Night	false.pos
W1873	sway.y	testing.generic	0.1	5	0	1	272	83.8	59.95	12.73	2.6	1	1	3	forage	no prey	0	1	no prey	no prey	no prey	Night	false.pos
W1873	sway.y	testing.generic	0.1	5	1	2	272	83.8	141.95	50.41	8.8	3	1	3	forage	fish	1	1	chase.cap.hand	successful	ascent	Night	true.pos
W1873	sway.y	testing.generic	0.1	5	0	3	272	83.8	204	0.39	0.15	1	1	3	forage	no prey	0	1	no prey	no prey	no prey	Night	false.pos
W1873	sway.y	testing.generic	0.1	5	1	1	273	83.8	67.35	49.59	4.7	1	3	6	forage	unknown	1	1	chase.cap.hand	successful	benthic	Night	true.pos
W1873	sway.y	testing.generic	0.1	5	1	2	273	83.8	89.9	64.66	4.9	1	3	6	forage	unknown	0	1	chase.cap.hand	successful	benthic	Night	false.pos
W1873	sway.y	testing.generic	0.1	5	2	3	273	83.8	123.5	32.12	4.05	2	3	6	forage	fish	1	1	chase.cap.hand	successful	benthic	Night	true.pos
W1873	sway.y	testing.generic	0.1	5	2	4	273	83.8	131.1	49.59	4.7	1	3	6	forage	fish	0	1	chase.cap.hand	successful	benthic	Night	false.pos
W1873	sway.y	testing.generic	0.1	5	0	5	273	83.8	162.9	64.66	4.9	1	3	6	forage	no prey	0	1	no prey	no prey	no prey	Night	false.pos
W1873	sway.y	testing.generic	0.1	5	3	6	273	83.8	220.95	32.12	4.05	2	3	6	forage	fish	1	1	chase.cap.hand	successful	ascent	Night	true.pos
W1873	sway.y	testing.generic	0.1	5	0	1	280	84.3	20.3	6.45	1.55	1	3	4	forage	no prey	0	1	no prey	no prey	no prey	Night	false.pos
W1873	sway.y	testing.generic	0.1	5	1	2	280	84.3	67.2	66.31	8.85	3	3	4	forage	fish	1	1	chase.cap.hand	successful	benthic	Night	true.pos
W1873	sway.y	testing.generic	0.1	5	2	3	280	84.3	105.8	32.62	5.55	3	3	4	forage	fish	1	1	chase.cap.hand	successful	benthic	Night	true.pos
W1873	sway.y	testing.generic	0.1	5	3	4	280	84.3	149.15	29.75	6	1	3	4	forage	fish	1	1	chase.cap.hand	successful	benthic	Night	true.pos
W1873	sway.y	testing.generic	0.1	5	0	1	290	84.8	50.65	12.31	1.75	1	2	4	forage	no prey	0	1	no prey	no prey	no prey	Night	false.pos
W1873	sway.y	testing.generic	0.1	5	1	2	290	84.8	66.9	51.93	8.95	2	2	4	forage	fish	1	1	chase.cap.hand	successful	benthic	Night	true.pos
W1873	sway.y	testing.generic	0.1	5	1	3	290	84.8	78.4	54.87	10.95	1	2	4	forage	fish	0	1	chase.cap.hand	successful	benthic	Night	false.pos
W1873	sway.y	testing.generic	0.1	5	2	4	290	84.8	134.45	12.31	1.75	6	2	4	forage	fish	1	1	chase.cap.hand	successful	ascent	Night	true.pos
W1873	sway.y	testing.generic	0.1	5	1	1	294	84.8	57.2	44.06	7	3	3	6	forage	fish	1	1	chase.cap.hand	successful	benthic	Night	true.pos
W1873	sway.y	testing.generic	0.1	5	2	2	294	84.8	71.55	33.72	5.5	1	3	6	forage	fish	1	1	chase.cap.hand	successful	benthic	Night	true.pos
W1873	sway.y	testing.generic	0.1	5	2	3	294	84.8	79.1	24.72	5.35	3	3	6	forage	fish	0	1	chase.cap.hand	successful	benthic	Night	false.pos
W1873	sway.y	testing.generic	0.1	5	2	4	294	84.8	91.8	4.04	0.75	3	3	6	forage	fish	0	1	chase.cap.hand	successful	benthic	Night	false.pos
W1873	sway.y	testing.generic	0.1	5	3	5	294	84.8	122.3	44.06	7	1	3	6	forage	fish	1	1	chase.cap.hand	successful	ascent	Night	true.pos
W1873	sway.y	testing.generic	0.1	5	0	6	294	84.8	169.2	33.72	5.5	1	3	6	forage	no prey	0	1	no prey	no prey	no prey	Night	false.pos
W1873	sway.y	testing.generic	0.1	5	1	1	295	84.8	68.65	36.77	5.9	1	3	7	forage	fish	1	1	chase.cap.hand	successful	benthic	Night	true.pos
W1873	sway.y	testing.generic	0.1	5	1	2	295	84.8	74.7	6.07	1.95	1	3	7	forage	fish	0	1	chase.cap.hand	successful	benthic	Night	false.pos
W1873	sway.y	testing.generic	0.1	5	2	3	295	84.8	96.75	2.46	0.75	1	3	7	forage	fish	1	1	chase.cap.hand	successful	benthic	Night	true.pos
W1873	sway.y	testing.generic	0.1	5	2	4	295	84.8	111.5	36.77	5.9	1	3	7	forage	fish	0	1	chase.cap.hand	successful	benthic	Night	false.pos
W1873	sway.y	testing.generic	0.1	5	2	5	295	84.8	120.4	6.07	1.95	2	3	7	forage	fish	0	1	chase.cap.hand	successful	benthic	Night	false.pos
W1873	sway.y	testing.generic	0.1	5	3	6	295	84.8	164.85	2.46	0.75	1	3	7	forage	fish	1	1	chase.cap	successful	ascent	Night	true.pos
W1873	sway.y	testing.generic	0.1	5	0	7	295	84.8	208.4	36.77	5.9	1	3	7	forage	no prey	0	1	no prey	no prey	no prey	Night	false.pos
W1873	sway.y	testing.generic	0.1	5	1	1	297	84.8	53.05	16.39	2.75	1	6	7	forage	fish	1	1	chase.cap.hand	successful	benthic	Night	true.pos
W1873	sway.y	testing.generic	0.1	5	2	2	297	84.8	76.5	14.44	2.45	1	6	7	forage	fish	1	1	chase.cap.hand	successful	benthic	Night	true.pos
W1873	sway.y	testing.generic	0.1	5	3	3	297	84.8	82.35	50.27	8.65	2	6	7	forage	fish	1	1	chase.cap.hand	successful	benthic	Night	true.pos
W1873	sway.y	testing.generic	0.1	5	3	4	297	84.8	88.85	7.14	1.85	1	6	7	forage	fish	0	1	chase.cap.hand	successful	benthic	Night	false.pos
W1873	sway.y	testing.generic	0.1	5	4	5	297	84.8	106.45	16.39	2.75	3	6	7	forage	fish	1	1	chase.cap.hand	successful	benthic	Night	true.pos
W1873	sway.y	testing.generic	0.1	5	5	6	297	84.8	136.75	14.44	2.45	1	6	7	forage	fish	1	1	chase.cap.hand	successful	benthic	Night	true.pos
W1873	sway.y	testing.generic	0.1	5	6	7	297	84.8	160.4	50.27	8.65	1	6	7	forage	fish	1	1	chase.cap	successful	ascent	Night	true.pos
W1873	sway.y	testing.generic	0.1	5	1	1	327	84.3	65.75	18.17	4.3	1	1	3	forage	unknown	1	1	cap.hand	successful	ascent	Day	true.pos
W1873	sway.y	testing.generic	0.1	5	1	2	327	84.3	148.8	2.72	1.15	1	1	3	forage	unknown	0	1	cap.hand	successful	ascent	Day	false.pos
W1873	sway.y	testing.generic	0.1	5	0	3	327	84.3	217.25	2.47	1.2	1	1	3	forage	no prey	0	1	no prey	no prey	no prey	Day	false.pos
W1873	sway.y	testing.generic	0.1	5	0	1	360	85.3	55.45	1.01	0.45	1	0	4	control	no prey	0	1	no prey	no prey	no prey	Night	false.pos
W1873	sway.y	testing.generic	0.1	5	0	2	360	85.3	103.25	5.2	1.5	1	0	4	control	no prey	0	1	no prey	no prey	no prey	Night	false.pos
W1873	sway.y	testing.generic	0.1	5	0	3	360	85.3	169.75	3.86	1.45	1	0	4	control	no prey	0	1	no prey	no prey	no prey	Night	false.pos
W1873	sway.y	testing.generic	0.1	5	0	4	360	85.3	180.4	4.05	1.5	1	0	4	control	no prey	0	1	no prey	no prey	no prey	Night	false.pos
W1873	sway.y	testing.generic	0.1	5	1	1	362	85.2	80.35	20.39	2.8	1	1	4	forage	fish	1	1	chase.cap.hand	successful	ascent	Night	true.pos
W1873	sway.y	testing.generic	0.1	5	1	2	362	85.2	85.55	40	4.7	1	1	4	forage	fish	0	1	chase.cap.hand	successful	ascent	Night	false.pos
W1873	sway.y	testing.generic	0.1	5	1	3	362	85.2	132.1	3.17	1.45	1	1	4	forage	fish	0	1	chase.cap.hand	successful	ascent	Night	false.pos
W1873	sway.y	testing.generic	0.1	5	1	4	362	85.2	161.2	3.47	1.45	1	1	4	forage	fish	0	1	chase.cap.hand	successful	ascent	Night	false.pos
W1873	sway.y	testing.generic	0.1	5	0	1	363	85.2	43.6	3.29	1.45	1	2	4	forage	no prey	0	1	no prey	no prey	no prey	Night	false.pos
W1873	sway.y	testing.generic	0.1	5	0	2	363	85.2	49.95	42.18	6.75	1	2	4	forage	no prey	0	1	no prey	no prey	no prey	Night	false.pos
W1873	sway.y	testing.generic	0.1	5	1	3	363	85.2	85.95	12.06	2.8	4	2	4	forage	fish	1	1	chase.cap.hand	successful	benthic	Night	true.pos
W1873	sway.y	testing.generic	0.1	5	2	4	363	85.2	137.4	3.29	1.45	1	2	4	forage	fish	1	1	chase(miss)	unsuccessful	benthic	Night	true.pos
W1873	heave.z	testing.generic	0.1	5	0	0	27	NA	NA	NA	NA	NA	NA	NA	control	no prey	0	0	no prey	no prey	no prey	Night	true.neg
W1873	heave.z	testing.generic	0.1	5	0	0	28	NA	NA	NA	NA	NA	NA	NA	control	no prey	0	0	no prey	no prey	no prey	Night	true.neg
W1873	heave.z	testing.generic	0.1	5	0	0	29	NA	NA	NA	NA	NA	NA	NA	control	no prey	0	0	no prey	no prey	no prey	Night	true.neg
W1873	heave.z	testing.generic	0.1	5	0	0	31	NA	NA	NA	NA	NA	NA	NA	control	no prey	0	0	no prey	no prey	no prey	Night	true.neg
W1873	heave.z	testing.generic	0.1	5	1	1	58	81	116.15	12.02	3.85	2	1	1	forage	fish	1	1	chase.cap.hand	successful	ascent	Day	true.pos
W1873	heave.z	testing.generic	0.1	5	0	0	59	NA	NA	NA	NA	NA	NA	NA	control	no prey	0	0	no prey	no prey	no prey	Day	true.neg
W1873	heave.z	testing.generic	0.1	5	0	0	86	NA	NA	NA	NA	NA	NA	NA	control	no prey	0	0	no prey	no prey	no prey	Day	true.neg
W1873	heave.z	testing.generic	0.1	5	1	1	107	83.4	67.3	9.28	1.5	1	4	5	forage	fish	1	1	chase.cap.hand	successful	benthic	Night	true.pos
W1873	heave.z	testing.generic	0.1	5	1	2	107	83.4	78.05	8.21	3	2	4	5	forage	fish	0	1	chase.cap.hand	successful	benthic	Night	false.pos
W1873	heave.z	testing.generic	0.1	5	3	3	107	83.4	174.55	12.19	2	1	4	5	forage	fish	1	1	chase.cap.hand	successful	benthic	Night	true.pos
W1873	heave.z	testing.generic	0.1	5	3	4	107	83.4	184.65	4.29	1.5	2	4	5	forage	fish	0	1	chase.cap.hand	successful	benthic	Night	false.pos
W1873	heave.z	testing.generic	0.1	5	4	5	107	83.4	267.35	9.28	1.5	1	4	5	forage	fish	1	1	chase.cap.hand	successful	ascent	Night	true.pos
W1873	heave.z	testing.generic	0.1	5	2	0	107	NA	NA	NA	NA	NA	NA	NA	forage	fish	1	0	chase.cap.hand	successful	benthic	Night	false.neg
W1873	heave.z	testing.generic	0.1	5	0	1	112	83.4	180.95	27.35	5.05	3	0	1	control	no prey	0	1	no prey	no prey	no prey	Night	false.pos
W1873	heave.z	testing.generic	0.1	5	1	1	113	83.9	61.25	23.65	5.85	5	4	3	forage	fish	1	1	chase.cap.hand	successful	benthic	Night	true.pos
W1873	heave.z	testing.generic	0.1	5	2	2	113	83.9	100.6	3.01	1.35	1	4	3	forage	fish	1	1	chase.cap.hand	successful	benthic	Night	true.pos
W1873	heave.z	testing.generic	0.1	5	4	3	113	83.9	171.2	23.65	5.85	1	4	3	forage	fish	1	1	chase.cap	successful	ascent	Night	true.pos
W1873	heave.z	testing.generic	0.1	5	3	0	113	NA	NA	NA	NA	NA	NA	NA	forage	fish	1	0	cap.hand	successful	benthic	Night	false.neg
W1873	heave.z	testing.generic	0.1	5	1	1	130	84.9	58.45	3.4	1.45	2	2	2	forage	fish	1	1	chase.cap.hand	successful	benthic	Night	true.pos
W1873	heave.z	testing.generic	0.1	5	2	2	130	84.9	110.3	3.96	1.45	1	2	2	forage	fish	1	1	chase.cap.hand	successful	benthic	Night	true.pos
W1873	heave.z	testing.generic	0.1	5	1	1	131	85.4	61.3	16.14	1.65	1	7	8	forage	unknown	1	1	chase.cap.hand	successful	benthic	Night	true.pos
W1873	heave.z	testing.generic	0.1	5	2	2	131	85.4	103	6.94	1.5	1	7	8	forage	fish	1	1	cap.hand	successful	benthic	Night	true.pos
W1873	heave.z	testing.generic	0.1	5	4	3	131	85.4	121.25	1.79	0.75	1	7	8	forage	fish	1	1	chase.cap.hand	successful	benthic	Night	true.pos
W1873	heave.z	testing.generic	0.1	5	4	4	131	85.4	133.7	3.79	1.45	1	7	8	forage	fish	0	1	chase.cap.hand	successful	benthic	Night	false.pos
W1873	heave.z	testing.generic	0.1	5	5	5	131	85.4	149.05	6.19	1.5	1	7	8	forage	fish	1	1	chase.cap.hand	successful	benthic	Night	true.pos
W1873	heave.z	testing.generic	0.1	5	5	6	131	85.4	163.3	0.15	0.05	1	7	8	forage	fish	0	1	chase.cap.hand	successful	benthic	Night	false.pos
W1873	heave.z	testing.generic	0.1	5	6	7	131	85.4	169.9	0.16	0.05	1	7	8	forage	fish	1	1	chase.cap.hand	successful	benthic	Night	true.pos
W1873	heave.z	testing.generic	0.1	5	6	8	131	85.4	182.3	3.25	1.45	1	7	8	forage	fish	0	1	chase.cap.hand	successful	benthic	Night	false.pos
W1873	heave.z	testing.generic	0.1	5	3	0	131	NA	NA	NA	NA	NA	NA	NA	forage	fish	1	0	chase.cap.hand	successful	benthic	Night	false.neg
W1873	heave.z	testing.generic	0.1	5	7	0	131	NA	NA	NA	NA	NA	NA	NA	forage	fish	1	0	chase.cap	successful	ascent	Night	false.neg
W1873	heave.z	testing.generic	0.1	5	1	1	132	84.9	102.05	13.07	3.05	2	5	5	forage	unknown	1	1	chase.cap.hand	successful	benthic	Night	true.pos
W1873	heave.z	testing.generic	0.1	5	2	2	132	84.9	120.15	2.09	0.9	1	5	5	forage	fish	1	1	chase.cap.hand	successful	benthic	Night	true.pos
W1873	heave.z	testing.generic	0.1	5	3	3	132	84.9	127.7	13.07	3.05	1	5	5	forage	fish	1	1	chase.cap.hand	successful	benthic	Night	true.pos
W1873	heave.z	testing.generic	0.1	5	4	4	132	84.9	136.5	2.09	0.9	1	5	5	forage	unknown	1	1	chase.cap.hand	successful	benthic	Night	true.pos
W1873	heave.z	testing.generic	0.1	5	0	5	132	84.9	201.15	13.07	3.05	1	5	5	forage	no prey	0	1	no prey	no prey	no prey	Night	false.pos
W1873	heave.z	testing.generic	0.1	5	5	0	132	NA	NA	NA	NA	NA	NA	NA	forage	unknown	1	0	cap.hand	successful	benthic	Night	false.neg
W1873	heave.z	testing.generic	0.1	5	1	1	133	84.9	56.45	0.26	0.1	1	4	4	forage	fish	1	1	chase.cap.hand	successful	benthic	Night	true.pos
W1873	heave.z	testing.generic	0.1	5	2	2	133	84.9	102.6	22.21	4.55	3	4	4	forage	fish	1	1	chase.cap.hand	successful	benthic	Night	true.pos
W1873	heave.z	testing.generic	0.1	5	3	3	133	84.9	131.45	9.33	2.6	2	4	4	forage	fish	1	1	cap.hand	successful	benthic	Night	true.pos
W1873	heave.z	testing.generic	0.1	5	4	4	133	84.9	149.3	8.56	2.95	2	4	4	forage	fish	1	1	chase.cap.hand	successful	ascent	Night	true.pos
W1873	heave.z	testing.generic	0.1	5	1	1	136	85.4	61.7	24.07	5.15	4	5	5	forage	fish	1	1	chase.cap.hand	successful	benthic	Night	true.pos
W1873	heave.z	testing.generic	0.1	5	3	2	136	85.4	105.6	9.61	2.85	1	5	5	forage	fish	1	1	chase.cap.hand	successful	benthic	Night	true.pos
W1873	heave.z	testing.generic	0.1	5	3	3	136	85.4	119.3	7.11	2.45	2	5	5	forage	fish	0	1	chase.cap.hand	successful	benthic	Night	false.pos
W1873	heave.z	testing.generic	0.1	5	4	4	136	85.4	158.55	7.91	2.8	2	5	5	forage	fish	1	1	chase.cap.hand	successful	benthic	Night	true.pos
W1873	heave.z	testing.generic	0.1	5	5	5	136	85.4	196.9	24.07	5.15	2	5	5	forage	fish	1	1	chase(miss)	unsuccessful	benthic	Night	true.pos
W1873	heave.z	testing.generic	0.1	5	2	0	136	NA	NA	NA	NA	NA	NA	NA	forage	fish	1	0	chase.cap.hand	successful	benthic	Night	false.neg
W1873	heave.z	testing.generic	0.1	5	1	1	138	85.4	104.8	7.67	1.55	1	4	5	forage	unknown	1	1	chase.cap.hand	successful	benthic	Night	true.pos
W1873	heave.z	testing.generic	0.1	5	2	2	138	85.4	123.85	6.11	1.6	1	4	5	forage	fish	1	1	chase.cap.hand	successful	benthic	Night	true.pos
W1873	heave.z	testing.generic	0.1	5	3	3	138	85.4	134.7	4.07	1.4	1	4	5	forage	unknown	1	1	cap.hand	successful	benthic	Night	true.pos
W1873	heave.z	testing.generic	0.1	5	4	4	138	85.4	180.55	12.85	1.75	1	4	5	forage	fish	1	1	chase.cap	successful	ascent	Night	true.pos
W1873	heave.z	testing.generic	0.1	5	4	5	138	85.4	187.1	8.09	1.6	1	4	5	forage	fish	0	1	chase.cap	successful	ascent	Night	false.pos
W1873	heave.z	testing.generic	0.1	5	1	1	181	85.4	164.5	2.91	1.2	1	1	2	forage	fish	1	1	chase.cap.hand	successful	ascent	Day	true.pos
W1873	heave.z	testing.generic	0.1	5	1	2	181	85.4	172.75	3.57	1.4	1	1	2	forage	fish	0	1	chase.cap.hand	successful	ascent	Day	false.pos
W1873	heave.z	testing.generic	0.1	5	1	0	182	NA	NA	NA	NA	NA	NA	NA	forage	unknown	1	0	cap.hand	successful	ascent	Day	false.neg
W1873	heave.z	testing.generic	0.1	5	0	0	183	NA	NA	NA	NA	NA	NA	NA	control	no prey	0	0	no prey	no prey	no prey	Day	true.neg
W1873	heave.z	testing.generic	0.1	5	0	0	184	NA	NA	NA	NA	NA	NA	NA	control	no prey	0	0	no prey	no prey	no prey	Day	true.neg
W1873	heave.z	testing.generic	0.1	5	0	0	225	NA	NA	NA	NA	NA	NA	NA	control	no prey	0	0	no prey	no prey	no prey	Day	true.neg
W1873	heave.z	testing.generic	0.1	5	0	0	226	NA	NA	NA	NA	NA	NA	NA	control	no prey	0	0	no prey	no prey	no prey	Day	true.neg
W1873	heave.z	testing.generic	0.1	5	0	0	228	NA	NA	NA	NA	NA	NA	NA	control	no prey	0	0	no prey	no prey	no prey	Day	true.neg
W1873	heave.z	testing.generic	0.1	5	1	0	232	NA	NA	NA	NA	NA	NA	NA	forage	unknown	1	0	chase.cap.hand	successful	benthic	Day	false.neg
W1873	heave.z	testing.generic	0.1	5	0	0	233	NA	NA	NA	NA	NA	NA	NA	control	no prey	0	0	no prey	no prey	no prey	Day	true.neg
W1873	heave.z	testing.generic	0.1	5	1	1	235	84.4	113.1	18.65	3.9	1	1	1	forage	unknown	1	1	chase.cap.hand	successful	benthic	Day	true.pos
W1873	heave.z	testing.generic	0.1	5	1	1	236	84.4	87.3	3.79	1.45	1	1	1	forage	unknown	1	1	cap.hand	successful	benthic	Day	true.pos
W1873	heave.z	testing.generic	0.1	5	1	1	269	85.3	63.3	7.56	1.55	1	1	1	forage	fish	1	1	chase.cap.hand	successful	benthic	Night	true.pos
W1873	heave.z	testing.generic	0.1	5	1	1	271	83.8	82	14.14	1.65	1	2	3	forage	fish	1	1	chase.cap.hand	successful	benthic	Night	true.pos
W1873	heave.z	testing.generic	0.1	5	1	2	271	83.8	87.15	8	2.85	2	2	3	forage	fish	0	1	chase.cap.hand	successful	benthic	Night	false.pos
W1873	heave.z	testing.generic	0.1	5	2	3	271	83.8	119.5	2.85	1.2	1	2	3	forage	fish	1	1	chase.cap.hand	successful	benthic	Night	true.pos
W1873	heave.z	testing.generic	0.1	5	0	1	272	83.8	60	4.79	1.5	1	1	3	forage	no prey	0	1	no prey	no prey	no prey	Night	false.pos
W1873	heave.z	testing.generic	0.1	5	1	2	272	83.8	141.85	28.09	4.5	2	1	3	forage	fish	1	1	chase.cap.hand	successful	ascent	Night	true.pos
W1873	heave.z	testing.generic	0.1	5	1	3	272	83.8	151.2	3.75	1.5	1	1	3	forage	fish	0	1	chase.cap.hand	successful	ascent	Night	false.pos
W1873	heave.z	testing.generic	0.1	5	1	1	273	83.8	67.25	9.74	1.8	1	3	5	forage	unknown	1	1	chase.cap.hand	successful	benthic	Night	true.pos

W1873	heave.z	testing.generic	0.1	5	1	2	273	83.8	89.9	3.4	1.45	1	3	5	forage	unknown	0	1	chase.cap.hand	successful	benthic	Night	false.pos
W1873	heave.z	testing.generic	0.1	5	2	3	273	83.8	125.8	13.37	2.65	1	3	5	forage	fish	1	1	chase.cap.hand	successful	benthic	Night	true.pos
W1873	heave.z	testing.generic	0.1	5	2	4	273	83.8	131.15	3.62	1.35	1	3	5	forage	fish	0	1	chase.cap.hand	successful	benthic	Night	false.pos
W1873	heave.z	testing.generic	0.1	5	3	5	273	83.8	221	2.21	0.85	1	3	5	forage	fish	1	1	chase.cap.hand	successful	ascent	Night	true.pos
W1873	heave.z	testing.generic	0.1	5	1	1	280	84.3	67	23.17	4.55	3	3	3	forage	fish	1	1	chase.cap.hand	successful	benthic	Night	true.pos
W1873	heave.z	testing.generic	0.1	5	2	2	280	84.3	111.9	3.32	1.35	1	3	3	forage	fish	1	1	chase.cap.hand	successful	benthic	Night	true.pos
W1873	heave.z	testing.generic	0.1	5	3	3	280	84.3	149	23.17	4.55	1	3	3	forage	fish	1	1	chase.cap.hand	successful	benthic	Night	true.pos
W1873	heave.z	testing.generic	0.1	5	2	1	290	84.8	138.5	8.06	2.9	2	2	1	forage	fish	1	1	chase.cap.hand	successful	ascent	Night	true.pos
W1873	heave.z	testing.generic	0.1	5	1	0	290	NA	NA	NA	NA	NA	NA	NA	forage	fish	1	0	chase.cap.hand	successful	benthic	Night	false.neg
W1873	heave.z	testing.generic	0.1	5	1	1	294	84.8	61.4	9.62	2.75	3	3	4	forage	fish	1	1	chase.cap.hand	successful	benthic	Night	true.pos
W1873	heave.z	testing.generic	0.1	5	2	2	294	84.8	82.25	5.37	2.25	2	3	4	forage	fish	1	1	chase.cap.hand	successful	benthic	Night	true.pos
W1873	heave.z	testing.generic	0.1	5	3	3	294	84.8	119.25	29.22	5.65	3	3	4	forage	fish	1	1	chase.cap.hand	successful	ascent	Night	true.pos
W1873	heave.z	testing.generic	0.1	5	0	4	294	84.8	169.2	1.14	0.35	1	3	4	forage	no prey	0	1	no prey	no prey	no prey	Night	false.pos
W1873	heave.z	testing.generic	0.1	5	1	1	295	84.8	68.65	7.23	1.6	1	3	3	forage	fish	1	1	chase.cap.hand	successful	benthic	Night	true.pos
W1873	heave.z	testing.generic	0.1	5	2	2	295	84.8	110.25	8.27	1.75	1	3	3	forage	fish	1	1	chase.cap.hand	successful	benthic	Night	true.pos
W1873	heave.z	testing.generic	0.1	5	3	3	295	84.8	164.9	3.53	1.4	1	3	3	forage	fish	1	1	chase.cap	successful	ascent	Night	true.pos
W1873	heave.z	testing.generic	0.1	5	1	1	297	84.8	52.95	4.49	1.7	1	6	5	forage	fish	1	1	chase.cap.hand	successful	benthic	Night	true.pos
W1873	heave.z	testing.generic	0.1	5	2	2	297	84.8	81.35	9.24	2.2	1	6	5	forage	fish	1	1	chase.cap.hand	successful	benthic	Night	true.pos
W1873	heave.z	testing.generic	0.1	5	3	3	297	84.8	88.8	4.49	1.7	1	6	5	forage	fish	1	1	chase.cap.hand	successful	benthic	Night	true.pos
W1873	heave.z	testing.generic	0.1	5	4	4	297	84.8	111.1	9.24	2.2	1	6	5	forage	fish	1	1	chase.cap.hand	successful	benthic	Night	true.pos
W1873	heave.z	testing.generic	0.1	5	5	5	297	84.8	136.75	4.49	1.7	2	6	5	forage	fish	1	1	chase.cap.hand	successful	benthic	Night	true.pos
W1873	heave.z	testing.generic	0.1	5	6	0	297	NA	NA	NA	NA	NA	NA	NA	forage	fish	1	0	chase.cap	successful	ascent	Night	false.neg
W1873	heave.z	testing.generic	0.1	5	1	1	327	84.3	65.7	4.47	1.5	1	1	1	forage	unknown	1	1	cap.hand	successful	ascent	Day	true.pos
W1873	heave.z	testing.generic	0.1	5	0	0	360	NA	NA	NA	NA	NA	NA	NA	control	no prey	0	0	no prey	no prey	no prey	Day	true.neg
W1873	heave.z	testing.generic	0.1	5	1	1	362	85.2	84.05	0.15	0.05	1	1	1	forage	fish	1	1	chase.cap.hand	successful	ascent	Night	true.pos
W1873	heave.z	testing.generic	0.1	5	1	1	363	85.2	90.75	19.11	1.7	1	2	2	forage	fish	1	1	chase.cap.hand	successful	benthic	Night	true.pos
W1873	heave.z	testing.generic	0.1	5	2	2	363	85.2	137.35	0.31	0.1	1	2	2	forage	fish	1	1	chase(miss)	unsuccessful	benthic	Night	true.pos
W1881	surge.x	testing.generic	0.1	5	1	1	49	67.5	51.25	6.26	1.5	1	3	4	forage	fish	1	1	chase.cap.hand	successful	benthic	Night	true.pos
W1881	surge.x	testing.generic	0.1	5	1	2	49	67.5	60.85	26.64	5.5	4	3	4	forage	fish	0	1	chase.cap.hand	successful	benthic	Night	false.pos
W1881	surge.x	testing.generic	0.1	5	2	3	49	67.5	137.45	46.34	7.75	1	3	4	forage	fish	1	1	chase.cap.hand	successful	benthic	Night	true.pos
W1881	surge.x	testing.generic	0.1	5	3	4	49	67.5	190.7	6.26	1.5	4	3	4	forage	fish	1	1	chase.cap.hand	successful	ascent	Night	true.pos
W1881	surge.x	testing.generic	0.1	5	1	1	50	67.5	184.1	15.97	3.05	2	1	2	forage	fish	1	1	chase.cap.hand	successful	ascent	Night	true.pos
W1881	surge.x	testing.generic	0.1	5	1	2	50	67.5	194.55	18.75	4.5	3	1	2	forage	fish	0	1	chase.cap.hand	successful	ascent	Night	false.pos
W1881	surge.x	testing.generic	0.1	5	1	1	57	68	63.3	38.31	6.2	3	2	4	forage	fish	1	1	chase.cap.hand	successful	benthic	Night	true.pos
W1881	surge.x	testing.generic	0.1	5	0	2	57	68	82.8	54.76	10.4	1	2	4	forage	no prey	0	1	no prey	no prey	no prey	Night	false.pos
W1881	surge.x	testing.generic	0.1	5	2	3	57	68	181.2	38.31	6.2	1	2	4	forage	fish	1	1	chase.cap.hand	successful	ascent	Night	true.pos
W1881	surge.x	testing.generic	0.1	5	2	4	57	68	192.2	54.76	10.4	5	2	4	forage	fish	0	1	chase.cap.hand	successful	ascent	Night	false.pos
W1881	surge.x	testing.generic	0.1	5	0	1	81	75.6	46.15	8.27	1.55	1	0	1	control	no prey	0	1	no prey	no prey	no prey	Night	false.pos
W1881	surge.x	testing.generic	0.1	5	1	1	82	75.1	78.3	4.03	1.45	1	3	5	forage	fish	1	1	chase.cap.hand	successful	benthic	Night	true.pos
W1881	surge.x	testing.generic	0.1	5	1	2	82	75.1	87.05	25.66	4.2	2	3	5	forage	fish	0	1	chase.cap.hand	successful	benthic	Night	false.pos
W1881	surge.x	testing.generic	0.1	5	2	3	82	75.1	115.15	21.71	3.2	1	3	5	forage	fish	1	1	cap.hand	successful	benthic	Night	true.pos
W1881	surge.x	testing.generic	0.1	5	3	4	82	75.1	148.1	4.03	1.45	1	3	5	forage	fish	1	1	chase.cap.hand	successful	benthic	Night	true.pos
W1881	surge.x	testing.generic	0.1	5	3	5	82	75.1	154.75	25.66	4.2	1	3	5	forage	fish	0	1	chase.cap.hand	successful	benthic	Night	false.pos
W1881	surge.x	testing.generic	0.1	5	1	1	83	75.1	78.8	48.81	4	1	4	5	forage	fish	1	1	cap.hand	successful	benthic	Night	true.pos
W1881	surge.x	testing.generic	0.1	5	2	2	83	75.1	116.7	8.96	2.95	1	4	5	forage	fish	1	1	chase(miss)	unsuccessful	benthic	Night	true.pos
W1881	surge.x	testing.generic	0.1	5	3	3	83	75.1	122.6	28.33	7	2	4	5	forage	fish	1	1	chase.cap.hand	successful	benthic	Night	true.pos
W1881	surge.x	testing.generic	0.1	5	3	4	83	75.1	133.15	11.98	3.1	3	4	5	forage	fish	0	1	chase.cap.hand	successful	benthic	Night	false.pos
W1881	surge.x	testing.generic	0.1	5	4	5	83	75.1	162.55	48.81	4	2	4	5	forage	unknown	1	1	cap.hand	successful	benthic	Night	true.pos
W1881	surge.x	testing.generic	0.1	5	1	1	107	78	59.05	29.08	5.85	2	2	5	forage	fish	1	1	chase.cap.hand	successful	benthic	Night	true.pos
W1881	surge.x	testing.generic	0.1	5	1	2	107	78	68.35	66.91	12.05	1	2	5	forage	fish	0	1	chase.cap.hand	successful	benthic	Night	false.pos
W1881	surge.x	testing.generic	0.1	5	1	3	107	78	76.35	3.57	1.45	5	2	5	forage	fish	0	1	chase.cap.hand	successful	benthic	Night	false.pos
W1881	surge.x	testing.generic	0.1	5	2	4	107	78	124.7	29.08	5.85	1	2	5	forage	fish	1	1	chase.cap.hand	successful	benthic	Night	true.pos
W1881	surge.x	testing.generic	0.1	5	0	5	107	78	188.1	66.91	12.05	1	2	5	forage	no prey	0	1	no prey	no prey	no prey	Night	false.pos
W1881	surge.x	testing.generic	0.1	5	1	1	144	79.6	85.6	30.55	6	4	3	7	forage	unknown	1	1	chase.cap.hand	successful	benthic	Day	true.pos
W1881	surge.x	testing.generic	0.1	5	0	2	144	79.6	113.85	17.32	2.85	1	3	7	forage	no prey	0	1	no prey	no prey	no prey	Day	false.pos
W1881	surge.x	testing.generic	0.1	5	0	3	144	79.6	149.1	22.37	3.3	1	3	7	forage	no prey	0	1	no prey	no prey	no prey	Day	false.pos
W1881	surge.x	testing.generic	0.1	5	2	4	144	79.6	154.15	29.36	3.25	2	3	7	forage	unknown	1	1	cap.hand	successful	benthic	Day	true.pos
W1881	surge.x	testing.generic	0.1	5	0	5	144	79.6	163.5	30.55	6	1	3	7	forage	no prey	0	1	no prey	no prey	no prey	Day	false.pos
W1881	surge.x	testing.generic	0.1	5	3	6	144	79.6	170.65	17.32	2.85	2	3	7	forage	unknown	1	1	chase(miss)	unsuccessful	benthic	Day	true.pos
W1881	surge.x	testing.generic	0.1	5	0	7	144	79.6	184.1	22.37	3.3	2	3	7	forage	no prey	0	1	no prey	no prey	no prey	Day	false.pos
W1881	surge.x	testing.generic	0.1	5	0	1	145	79.6	61.8	9.35	3	3	3	8	forage	no prey	0	1	no prey	no prey	no prey	Day	false.pos
W1881	surge.x	testing.generic	0.1	5	2	2	145	79.6	81.45	21.29	3.6	3	3	8	forage	unknown	1	1	cap.hand	successful	benthic	Day	true.pos
W1881	surge.x	testing.generic	0.1	5	2	3	145	79.6	92.6	47.36	5.5	1	3	8	forage	unknown	0	1	cap.hand	successful	benthic	Day	false.pos
W1881	surge.x	testing.generic	0.1	5	0	4	145	79.6	101	9.14	1.6	1	3	8	forage	no prey	0	1	no prey	no prey	no prey	Day	false.pos
W1881	surge.x	testing.generic	0.1	5	0	5	145	79.6	112.75	9.35	3	1	3	8	forage	no prey	0	1	no prey	no prey	no prey	Day	false.pos
W1881	surge.x	testing.generic	0.1	5	0	6	145	79.6	132.6	21.29	3.6	1	3	8	forage	no prey	0	1	no prey	no prey	no prey	Day	false.pos
W1881	surge.x	testing.generic	0.1	5	3	7	145	79.6	148.7	47.36	5.5	3	3	8	forage	unknown	1	1	chase(miss)	unsuccessful	benthic	Day	true.pos
W1881	surge.x	testing.generic	0.1	5	3	8	145	79.6	161.95	9.14	1.6	1	3	8	forage	unknown	0	1	chase(miss)	unsuccessful	benthic	Day	false.pos
W1881	surge.x	testing.generic	0.1	5	1	0	145	NA	NA	NA	NA	NA	NA	NA	forage	fish	1	0	cap.hand	successful	benthic	Day	false.neg
W1881	surge.x	testing.generic	0.1	5	1	1	146	79.6	52.3	23.9	2.85	1	4	8	forage	unknown	1	1	cap.hand	successful	benthic	Day	true.pos
W1881	surge.x	testing.generic	0.1	5	2	2	146	79.6	62.65	25.58	4.5	1	4	8	forage	fish	1	1	cap.hand	successful	benthic	Day	true.pos
W1881	surge.x	testing.generic	0.1	5	2	3	146	79.6	70.65	20.04	4.5	1	4	8	forage	fish	0	1	cap.hand	successful	benthic	Day	false.pos
W1881	surge.x	testing.generic	0.1	5	3	4	146	79.6	85.5	10.54	2.85	3	4	8	forage	fish	1	1	chase.cap.hand	successful	benthic	Day	true.pos
W1881	surge.x	testing.generic	0.1	5	0	5	146	79.6	121.25	23.9	2.85	1	4	8	forage	no prey	0	1	no prey	no prey	no prey	Day	false.pos
W1881	surge.x	testing.generic	0.1	5	4	6	146	79.6	143.2	25.58	4.5	1	4	8	forage	unknown	1	1	chase(no cap)	unsuccessful	ascent	Day	true.pos
W1881	surge.x	testing.generic	0.1	5	4	7	146	79.6	148.35	20.04	4.5	3	4	8	forage	unknown	0	1	chase.cap.hand	successful	ascent	Day	false.pos
W1881	surge.x	testing.generic	0.1	5	4	8	146	79.6	161.3	10.54	2.85	2	4	8	forage	unknown	0	1	chase.cap.hand	successful	ascent	Day	false.pos
W1881	surge.x	testing.generic	0.1	5	0	1	147	79.6	68.4	11.44	1.75	1	2	10	forage	no prey	0	1	no prey	no prey	no prey	Night	false.pos
W1881	surge.x	testing.generic	0.1	5	0	2	147	79.6	95.75	11.44	1.95	1	2	10	forage	no prey	0	1	no prey	no prey	no prey	Night	false.pos
W1881	surge.x	testing.generic	0.1	5	1	3	147	79.6	114.45	9.05	1.5	1	2	10	forage	unknown	1	1	cap.hand	successful	benthic	Night	true.pos
W1881	surge.x	testing.generic	0.1	5	0	4	147	79.6	129.15	11.44	1.75	1	2	10	forage	no prey	0	1	no prey	no prey	no prey	Night	false.pos
W1881	surge.x	testing.generic	0.1	5	0	5	147	79.6	139.15	11.44	1.95	1	2	10	forage	no prey	0	1	no prey	no prey	no prey	Night	false.pos
W1881	surge.x	testing.generic	0.1	5	2	6	147	79.6	145.7	9.05	1.5	1	2	10	forage	unknown	1	1	cap.hand	successful	benthic	Night	true.pos
W1881	surge.x	testing.generic	0.1	5	0	7	147	79.6	159.75	11.44	1.75	1	2	10	forage	no prey	0	1	no prey	no prey	no prey	Night	false.pos
W1881	surge.x	testing.generic	0.1	5	0	8	147	79.6	173.45	11.44	1.95	1	2	10	forage	no prey	0	1	no prey	no prey	no prey	Night	false.pos
W1881	surge.x	testing.generic	0.1	5	0	9	147	79.6	185.95	9.05	1.5	2	2	10	forage	no prey	0	1	no prey	no prey	no prey	Night	false.pos
W1881	surge.x	testing.generic	0.1	5	0	10	147	79.6	220.45	11.44	1.75	1	2	10	forage	no prey	0	1	no prey	no prey	no prey	Night	false.pos
W1881	surge.x	testing.generic	0.1	5	1	1	148	79.6	66.95	14.12	3	2	4	10	forage	unknown	1	1	cap.hand	successful	benthic	Night	true.pos
W1881	surge.x	testing.generic	0.1	5	2	2	148	79.6	79.3	45.01	3.9	1	4	10	forage	fish	1	1	cap.hand	successful	benthic	Night	true.pos
W1881	surge.x	testing.generic	0.1	5	2	3	148	79.6	88.35	47.18	4.15	2	4	10	forage	fish	0	1	cap.hand	successful	benthic	Night	false.pos
W1881	surge.x	testing.generic	0.1	5	0	4	148	79.6	99.6	9.97	1.6	1	4	10	forage	no prey	0	1	no prey	no prey	no prey	Night	false.pos
W1881	surge.x	testing.generic	0.1	5	0	5	148	79.6	106.15	14.12	3	1	4	10	forage	no prey	0	1	no prey	no prey	no prey	Night	false.pos
W1881	surge.x	testing.generic	0.1	5	0	6	148	79.6	116.45	45.01	3.9	1	4	10	forage	no prey	0	1	no prey	no prey	no prey	Night	false.pos
W1881	surge.x	testing.generic	0.1	5	3	7	148	79.6	127.45	47.18	4.15	1	4	10	forage	unknown	1	1	cap.hand	successful	benthic	Night	true.pos
W1881	surge.x	testing.generic	0.1	5	4	8	148	79.6	146.35	9.97	1.6	2	4	10	forage	unknown	1	1	cap.hand	successful	benthic	Night	true.pos
W1881	surge.x	testing.generic	0.1	5	4	9	148	79.6	154.9	14.12	3	1	4	10	forage	unknown	0	1	cap.hand	successful	benthic	Night	false.pos
W1881	surge.x	testing.generic	0.1	5	0	10	148	79.6	184.9	45.01	3.9	1	4	10	forage	no prey	0	1	no prey	no prey	no prey	Night	false.pos
W1881	surge.x	testing.generic	0.1	5	2	1	212	79.4	59.85	41.88	3.2	1	3	8	forage	fish	1	1	chase.cap.hand	successful	benthic	Night	true.pos
W1881	surge.x	testing.generic	0.1	5	2	2	212	79.4	65.65	11.16	1.55	1	3	8	forage	fish	0	1	chase.cap.hand	successful	benthic	Night	false.pos
W1881	surge.x	testing.generic	0.1	5	2	3	212	79.4	79.9	8.02	1.6	1	3	8	forage	fish	0	1	chase.cap.hand	successful	benthic	Night	false.pos
W1881	surge.x	testing.generic	0.1	5	2	4	212	79.4	124.25	11.21	1.6	1	3	8	forage	fish	0	1	chase.cap.hand	successful	benthic	Night	false.pos
W1881	surge.x	testing.generic	0.1	5	3	5	212	79.4	131.6	46.76	3.45	1	3	8	forage	fish	1	1	chase(miss)	unsuccessful	benthic	Night	true.pos
W1881	surge.x	testing.generic	0.1	5	3	6	212	79.4	153.1	6.11	1.5	1	3	8	forage	fish	0	1	chase(miss)	unsuccessful	benthic	Night	false.pos
W1881	surge.x	testing.generic	0.1	5	3	7	212	79.4	159.8	29.74	1.8	1	3	8	forage	fish	0	1	chase(miss)	unsuccessful	benthic	Night	false.pos
W1881	surge.x	testing.generic	0.1	5	3	8	212	79.4	179.35	3.3	1.25	1	3	8	forage	fish	0	1	chase(miss)	unsuccessful	benthic	Night	false.pos
W1881	surge.x	testing.generic	0.1	5	1	0	212	NA	NA	NA	NA	NA	NA	NA	forage	fish	1	0	chase.cap.hand	successful	benthic	Night	false.neg
W1881	surge.x	testing.generic	0.1	5	1	1	213	79.4	157.15	26.01	2.45	1	2	2	forage	fish	1	1	chase.cap.hand	successful	benthic	Night	true.pos
W1881	surge.x	testing.generic	0.1	5	2	2	213	79.4	234.9	19.28	5.2	3	2	2	forage	fish	1	1	chase.cap	successful	ascent	Night	true.pos
W1881	surge.x	testing.generic	0.1	5	1	1	214	78.9	46.25	16.61	3	2	2	9	forage	fish	1	1	chase.cap.hand	successful	benthic	Night	true.pos
W1881	surge.x	testing.generic	0.1	5	1	2	214	78.9	70.75	4.83	1.5	1	2	9	forage	fish	0	1	chase.cap.hand	successful	benthic	Night	false.pos
W1881	surge.x	testing.generic	0.1	5	2	3	214	78.9	87.2	16.61	3	1	2	9	forage	fish	1	1	chase(miss)	unsuccessful	benthic	Night	true.pos
W1881	surge.x	testing.generic	0.1	5	2	4	214	78.9	143.25	4.83	1.5	1	2	9	forage	fish	0	1	chase(miss)	unsuccessful	benthic	Night	false.pos
W1881	surge.x	testing.generic	0.1	5	2	5	214	78.9	172.8	16.61	3	1	2	9	forage	fish	0	1	chase(miss)	unsuccessful	benthic	Night	false.pos
W1881	surge.x	testing.generic	0.1	5	2	6	214	78.9	182.05	4.83	1.5	1	2	9	forage	fish	0	1	chase(miss)	unsuccessful	benthic	Night	false.pos
W1881	surge.x	testing.generic	0.1	5	2	7	214	78.9	188.7	16.61	3	1	2	9	forage	fish	0	1	chase(miss)	unsuccessful	benthic	Night	false.pos
W1881	surge.x	testing.generic	0.1	5	2	8	214	78.9	227.2	4.83	1.5	1	2	9	forage	fish	0	1	chase(miss)	unsuccessful	benthic	Night	false.pos
W1881	surge.x	testing.generic	0.1	5	2	9	214	78.9	250.65	16.61	3	1	2	9	forage	fish	0	1	chase(miss)	unsuccessful	benthic	Night	false.pos
W1881	surge.x	testing.generic	0.1	5	0	1	215	78.9	42.4	7.26	1.5	1	2	7	forage	no prey	0	1	no prey	no prey	no prey	Night	false.pos
W1881	surge.x	testing.generic	0.1	5	0	2	215	78.9	73.8	3.13	1.4	1	2	7	forage	no prey	0	1	no prey	no prey	no prey	Night	false.pos
W1881	surge.x	testing.generic	0.1	5	1	3	215	78.9	121.55	5.67	1.5	1	2	7	forage	fish	1	1	chase.cap.hand	successful	benthic	Night	true.pos
W1881	surge.x	testing.generic	0.1	5	2	4	215	78.9	155.8	11.49	2.5	1	2	7	forage	fish	1	1	chase.cap	successful	ascent	Night	true.pos
W1881	surge.x	testing.generic	0.1	5	2	5	215	78.9	169.9	21.94	1.7	1	2	7	forage	fish	0	1	chase.cap	successful	ascent	Night	false.pos
W1881	surge.x	testing.generic	0.1	5	2	6	215	78.9	182.85	8.42	1.5	1	2	7	forage	fish	0	1	chase.cap	successful	ascent	Night	false.pos
W1881	surge.x	testing.generic	0.1	5	2	7	215	78.9	228.5	20.05	1.85	1	2	7	forage	fish	0	1	chase.cap	successful	ascent	Night	false.pos
W1881	surge.x	testing.generic	0.1	5	1	1	217	79.4	53.5	6.15	1.5	1	2	11	forage	fish	1	1	chase.cap.hand	successful	benthic	Night	true.pos
W1881	surge.x	testing.generic	0.1	5	1	2	217	79.4	70.2	42.81	3.5	1	2	11	forage	fish	0	1	chase.cap.hand	successful	benthic	Night	false.pos
W1881	surge.x	testing.generic	0.1	5	0	3	217	79.4	94.95	7.31	1.55	1	2	11	forage	no prey	0	1	no prey	no prey	no prey	Night	false.pos
W1881	surge.x	testing.generic	0.1	5	0	4	217	79.4	102.1	43.98	2.45	1	2	11	forage	no prey	0	1	no prey	no prey	no prey	Night	false.pos
W1881	surge.x	testing.generic	0.1	5	0	5	217	79.4	118.2	10.59	1.55	1	2	11	forage	no prey	0	1	no prey	no prey	no prey	Night	false.pos
W1881	surge.x	testing.generic	0.1	5	0	6	217	79.4	125.8	14.13	1.75	1	2	11	forage	no prey	0	1	no prey	no prey	no prey	Night	false.pos
W1881	surge.x	testing.generic	0.1	5	0	7	217	79.4	139.3	9.08	1.55	1	2	11	forage	no prey	0	1	no prey	no prey	no prey	Night	false.pos
W1881	surge.x	testing.generic	0.1	5	2	8	217	79.4	157.25	19.58	1.6	1	2	11	forage	fish	1	1	chase.cap.hand	successful	benthic	Night	true.pos
W1881	surge.x	testing.generic	0.1	5	2	9	217	79.4	163.35	5.75	1.5	1	2	11	forage	fish	0	1	chase.cap.hand	successful	benthic	Night	false.pos
W1881	surge.x	testing.generic	0.1	5	2	10	217	79.4	209.8	25.81	2.85	1	2	11	forage	fish	0	1	chase.cap.hand	successful	benthic	Night	false.pos
W1881	surge.x	testing.generic	0.1	5	0	11	217	79.4	225.9	9.16	1.55	1	2	11	forage	no prey	0	1	no prey	no prey	no prey	Night	false.pos
W1881	surge.x	testing.generic	0.1	5	1	1	218	78.9	77.9	11.31	1.5	1	4	7	forage	fish	1	1	chase.cap.hand	successful	benthic	Night	true.pos
W1881	surge.x	testing.generic	0.1	5	1	2	218	78.9	108.3	45.33	6	1	4	7	forage	fish	0	1	chase.cap.hand	successful	benthic	Night	false.pos
W1881	surge.x	testing.generic	0.1	5	2	3	218	78.9	118.15	17.7	1.95	1	4	7	forage	fish	1	1	chase.cap.hand	successful	benthic	Night	true.pos
W1881	surge.x	testing.generic	0.1	5	3	4	218	78.9	168.65	11.31	1.5	1	4	7	forage	fish	1	1	chase.cap.hand	successful	benthic	Night	true.pos
W1881	surge.x	testing.generic	0.1	5	3	5	218	78.9	174.65	45.33	6	1	4	7	forage	fish	0	1	chase.cap.hand	successful	benthic	Night	false.pos
W1881	surge.x	testing.generic	0.1	5	3	6	218	78.9	183.8	17.7	1.95	2	4	7	forage	fish	0	1	chase.cap.hand	successful	benthic	Night	false.pos
W1881	surge.x	testing.generic	0.1	5	4	7	218	78.9	205.2	11.31	1.5	1	4	7	forage	fish	1	1	chase.cap.hand	successful	ascent	Night	true.pos
W1881	sway.y	testing.generic	0.1	5	1	1	49	67.5	48.95	23.6	4.7	3	3	4	forage	fish	1	1	chase.cap.hand	successful	benthic	Night	true.pos
W1881	sway.y	testing.generic	0.1	5	1	2	49	67.5	59.05	69.17	11.35	6	3	4	forage	fish	0	1	chase.cap.hand	successful	benthic	Night	false.pos
W1881	sway.y	testing.generic	0.1	5	2	3	49	67.5	137.5	93.4	11.35	1	3	4	forage	fish	1	1	chase.cap.hand	successful	benthic	Night	true.pos
W1881	sway.y	testing.generic	0.1	5	3	4	49	67.5	186.75	23.6	4.7	5	3	4	forage	fish	1	1	chase.cap.hand	successful	ascent	Night	true.pos
W1881	sway.y	testing.generic	0.1	5	0	1	50	67.5	53	3.92	1.45	1	1	4	forage	no prey	0	1	no prey	no prey	no prey	Night	false.pos
W1881	sway.y	testing.generic	0.1	5	1	2	50	67.5	169.55	43.6	5.95	1	1	4	forage	fish	1	1	chase.cap.hand	successful	ascent	Night	true.pos
W1881	sway.y	testing.generic	0.1	5	1	3	50	67.5	183.8	3.92	1.45	1	1	4	forage	fish	0	1	chase.cap.hand	successful	ascent	Night	false.pos
W1881	sway.y	testing.generic	0.1	5	1	4	50	67.5	192	43.6	5.95	4	1	4	forage	fish	0	1	chase.cap.hand	successful	ascent	Night	false.pos
W1881	sway.y	testing.generic	0.1	5	1	1	57	68	63.5	77.53	8.4	2	2	4	forage	fish	1	1	chase.cap.hand	successful	benthic	Night	true.pos
W1881	sway.y	testing.generic	0.1	5	0	2	57	68	82.3	44.76	3.95	2	2	4	forage	no prey	0	1	no prey	no prey	no prey	Night	false.pos
W1881	sway.y	testing.generic	0.1	5	2	3	57	68	181.5	16.34	3.1	2	2	4	forage	fish	1	1	chase.cap.hand	successful	ascent	Night	true.pos
W1881	sway.y	testing.generic	0.1	5	2	4	57	68	192.2	140.18	12.7	2	2	4	forage	fish	0	1	chase.cap.hand	successful	ascent	Night	false.pos
W1881	sway.y	testing.generic	0.1	5	0	1	81	75.6	260.1	2.76	1.2	1	0	1	control	no prey	0	1	no prey	no prey	no prey	Night	false.pos
W1881	sway.y	testing.generic	0.1	5	1	1	82	75.1	87.05	54.95	7.2	3	3	4	forage	fish	1	1	chase.cap.hand	successful	benthic	Night	true.pos
W1881	sway.y	testing.generic	0.1	5	2	2	82	75.1	115.2	50.12	6.85	1	3	4	forage	fish	1	1	cap.hand	successful	benthic	Night	true.pos

W1881	sway.y	testing.generic	0.1	5	3	3	82	75.1	148.2	54.95	7.2	1	3	4	forage	fish	1	1	chase.cap.hand	successful	benthic	Night	true.pos
W1881	sway.y	testing.generic	0.1	5	3	4	82	75.1	154.75	50.12	6.85	6	3	4	forage	fish	0	1	chase.cap.hand	successful	benthic	Night	false.pos
W1881	sway.y	testing.generic	0.1	5	1	1	83	75.1	79.7	68.08	4.1	1	4	6	forage	fish	1	1	cap.hand	successful	benthic	Night	true.pos
W1881	sway.y	testing.generic	0.1	5	2	2	83	75.1	116.45	3.79	1.45	1	4	6	forage	fish	1	1	chase(miss)	unsuccessful	benthic	Night	true.pos
W1881	sway.y	testing.generic	0.1	5	3	3	83	75.1	126.05	5.07	1.55	1	4	6	forage	fish	1	1	chase.cap.hand	successful	benthic	Night	true.pos
W1881	sway.y	testing.generic	0.1	5	3	4	83	75.1	133.05	51.69	4.25	1	4	6	forage	fish	0	1	chase.cap.hand	successful	benthic	Night	false.pos
W1881	sway.y	testing.generic	0.1	5	3	5	83	75.1	141.8	75.13	7.85	1	4	6	forage	fish	0	1	chase.cap.hand	successful	benthic	Night	false.pos
W1881	sway.y	testing.generic	0.1	5	4	6	83	75.1	162.15	48.73	6.6	1	4	6	forage	unknown	1	1	cap.hand	successful	benthic	Night	true.pos
W1881	sway.y	testing.generic	0.1	5	1	1	107	78	56.85	146.38	23.9	12	2	3	forage	fish	1	1	chase.cap.hand	successful	benthic	Night	true.pos
W1881	sway.y	testing.generic	0.1	5	2	2	107	78	99.05	88.35	16.7	10	2	3	forage	fish	1	1	chase.cap.hand	successful	benthic	Night	true.pos
W1881	sway.y	testing.generic	0.1	5	0	3	107	78	188	5.85	1.5	1	2	3	forage	no prey	0	1	no prey	no prey	no prey	Night	false.pos
W1881	sway.y	testing.generic	0.1	5	0	1	144	79.6	56.6	4.59	1.5	1	3	11	forage	no prey	0	1	no prey	no prey	no prey	Day	false.pos
W1881	sway.y	testing.generic	0.1	5	0	2	144	79.6	71.25	23.54	5.6	1	3	11	forage	no prey	0	1	no prey	no prey	no prey	Day	false.pos
W1881	sway.y	testing.generic	0.1	5	1	3	144	79.6	79.7	26.78	5	1	3	11	forage	unknown	1	1	chase.cap.hand	successful	benthic	Day	true.pos
W1881	sway.y	testing.generic	0.1	5	1	4	144	79.6	85.55	7.34	1.85	3	3	11	forage	unknown	0	1	chase.cap.hand	successful	benthic	Day	false.pos
W1881	sway.y	testing.generic	0.1	5	1	5	144	79.6	102.95	4.59	1.5	1	3	11	forage	unknown	0	1	chase.cap.hand	successful	benthic	Day	false.pos
W1881	sway.y	testing.generic	0.1	5	0	6	144	79.6	113.9	23.54	5.6	1	3	11	forage	no prey	0	1	no prey	no prey	no prey	Day	false.pos
W1881	sway.y	testing.generic	0.1	5	0	7	144	79.6	133.9	26.78	5	1	3	11	forage	no prey	0	1	no prey	no prey	no prey	Day	false.pos
W1881	sway.y	testing.generic	0.1	5	0	8	144	79.6	149.25	7.34	1.85	3	3	11	forage	no prey	0	1	no prey	no prey	no prey	Day	false.pos
W1881	sway.y	testing.generic	0.1	5	0	9	144	79.6	163.3	4.59	1.5	1	3	11	forage	no prey	0	1	no prey	no prey	no prey	Day	false.pos
W1881	sway.y	testing.generic	0.1	5	3	10	144	79.6	170.7	23.54	5.6	1	3	11	forage	unknown	1	1	chase(miss)	unsuccessful	benthic	Day	true.pos
W1881	sway.y	testing.generic	0.1	5	2	11	144	79.6	186.05	26.78	5	1	3	11	forage	unknown	1	0	cap.hand	successful	benthic	Day	false.neg
W1881	sway.y	testing.generic	0.1	5	0	1	145	79.6	61.5	9.16	1.6	1	3	8	forage	no prey	0	1	no prey	no prey	no prey	Day	false.pos
W1881	sway.y	testing.generic	0.1	5	1	2	145	79.6	70.25	24.99	4.7	1	3	8	forage	fish	1	1	cap.hand	successful	benthic	Day	true.pos
W1881	sway.y	testing.generic	0.1	5	2	3	145	79.6	85.65	12.07	2	1	3	8	forage	unknown	1	1	cap.hand	successful	benthic	Day	true.pos
W1881	sway.y	testing.generic	0.1	5	2	4	145	79.6	92.55	9.16	1.6	1	3	8	forage	unknown	0	1	cap.hand	successful	benthic	Day	false.pos
W1881	sway.y	testing.generic	0.1	5	0	5	145	79.6	101.05	24.99	4.7	1	3	8	forage	no prey	0	1	no prey	no prey	no prey	Day	false.pos
W1881	sway.y	testing.generic	0.1	5	0	6	145	79.6	112.85	12.07	2	1	3	8	forage	no prey	0	1	no prey	no prey	no prey	Day	false.pos
W1881	sway.y	testing.generic	0.1	5	3	7	145	79.6	148.55	9.16	1.6	3	3	8	forage	unknown	1	1	chase(miss)	unsuccessful	benthic	Day	true.pos
W1881	sway.y	testing.generic	0.1	5	3	8	145	79.6	162.15	24.99	4.7	1	3	8	forage	unknown	0	1	chase(miss)	unsuccessful	benthic	Day	false.pos
W1881	sway.y	testing.generic	0.1	5	1	1	146	79.6	53.1	53.1	3.9	1	4	10	forage	unknown	1	1	cap.hand	successful	benthic	Day	true.pos
W1881	sway.y	testing.generic	0.1	5	2	2	146	79.6	62.6	25.24	3.75	1	4	10	forage	fish	1	1	cap.hand	successful	benthic	Day	true.pos
W1881	sway.y	testing.generic	0.1	5	2	3	146	79.6	71.95	21.34	4.5	1	4	10	forage	fish	0	1	cap.hand	successful	benthic	Day	false.pos
W1881	sway.y	testing.generic	0.1	5	2	4	146	79.6	77.65	6.59	2.1	1	4	10	forage	fish	0	1	cap.hand	successful	benthic	Day	false.pos
W1881	sway.y	testing.generic	0.1	5	3	5	146	79.6	89.35	20.97	2.15	2	4	10	forage	fish	1	1	chase.cap.hand	successful	benthic	Day	true.pos
W1881	sway.y	testing.generic	0.1	5	0	6	146	79.6	106.45	53.1	3.9	1	4	10	forage	no prey	0	1	no prey	no prey	no prey	Day	false.pos
W1881	sway.y	testing.generic	0.1	5	4	7	146	79.6	142.85	25.24	3.75	1	4	10	forage	unknown	1	1	chase.cap.hand	successful	ascent	Day	true.pos
W1881	sway.y	testing.generic	0.1	5	4	8	146	79.6	148.45	21.34	4.5	4	4	10	forage	unknown	0	1	chase.cap.hand	successful	ascent	Day	false.pos
W1881	sway.y	testing.generic	0.1	5	4	9	146	79.6	161.9	6.59	2.1	2	4	10	forage	unknown	0	1	chase.cap.hand	successful	ascent	Day	false.pos
W1881	sway.y	testing.generic	0.1	5	4	10	146	79.6	169.9	20.97	2.15	1	4	10	forage	unknown	0	1	chase.cap.hand	successful	ascent	Day	false.pos
W1881	sway.y	testing.generic	0.1	5	0	1	147	79.6	68.5	12.68	2.4	1	2	8	forage	no prey	0	1	no prey	no prey	no prey	Night	false.pos
W1881	sway.y	testing.generic	0.1	5	1	2	147	79.6	114.55	2.3	0.9	1	2	8	forage	unknown	1	1	cap.hand	successful	benthic	Night	true.pos
W1881	sway.y	testing.generic	0.1	5	0	3	147	79.6	128.8	5.38	1.5	1	2	8	forage	no prey	0	1	no prey	no prey	no prey	Night	false.pos
W1881	sway.y	testing.generic	0.1	5	0	4	147	79.6	138.55	12.68	2.4	1	2	8	forage	no prey	0	1	no prey	no prey	no prey	Night	false.pos
W1881	sway.y	testing.generic	0.1	5	2	5	147	79.6	145.7	2.3	0.9	2	2	8	forage	unknown	1	1	cap.hand	successful	benthic	Night	true.pos
W1881	sway.y	testing.generic	0.1	5	0	6	147	79.6	159.9	5.38	1.5	1	2	8	forage	no prey	0	1	no prey	no prey	no prey	Night	false.pos
W1881	sway.y	testing.generic	0.1	5	0	7	147	79.6	186.3	12.68	2.4	1	2	8	forage	no prey	0	1	no prey	no prey	no prey	Night	false.pos
W1881	sway.y	testing.generic	0.1	5	0	8	147	79.6	220.45	2.3	0.9	1	2	8	forage	no prey	0	1	no prey	no prey	no prey	Night	false.pos
W1881	sway.y	testing.generic	0.1	5	1	1	148	79.6	61.95	6.55	2.05	1	4	11	forage	unknown	1	1	cap.hand	successful	benthic	Night	true.pos
W1881	sway.y	testing.generic	0.1	5	1	2	148	79.6	67	107.31	10.65	1	4	11	forage	unknown	0	1	cap.hand	successful	benthic	Night	false.pos
W1881	sway.y	testing.generic	0.1	5	1	3	148	79.6	73.6	71.43	5.25	1	4	11	forage	unknown	0	1	cap.hand	successful	benthic	Night	false.pos
W1881	sway.y	testing.generic	0.1	5	2	4	148	79.6	79	5.34	1.5	4	4	11	forage	fish	1	1	cap.hand	successful	benthic	Night	true.pos
W1881	sway.y	testing.generic	0.1	5	0	5	148	79.6	100	6.55	2.05	1	4	11	forage	no prey	0	1	no prey	no prey	no prey	Night	false.pos
W1881	sway.y	testing.generic	0.1	5	0	6	148	79.6	106.6	107.31	10.65	1	4	11	forage	no prey	0	1	no prey	no prey	no prey	Night	false.pos
W1881	sway.y	testing.generic	0.1	5	0	7	148	79.6	116.5	71.43	5.25	1	4	11	forage	no prey	0	1	no prey	no prey	no prey	Night	false.pos
W1881	sway.y	testing.generic	0.1	5	3	8	148	79.6	128.8	5.34	1.5	1	4	11	forage	unknown	1	1	cap.hand	successful	benthic	Night	true.pos
W1881	sway.y	testing.generic	0.1	5	4	9	148	79.6	146.2	6.55	2.05	2	4	11	forage	unknown	1	1	cap.hand	successful	benthic	Night	true.pos
W1881	sway.y	testing.generic	0.1	5	4	10	148	79.6	154.9	107.31	10.65	1	4	11	forage	unknown	0	1	cap.hand	successful	benthic	Night	false.pos
W1881	sway.y	testing.generic	0.1	5	0	11	148	79.6	184.6	71.43	5.25	1	4	11	forage	no prey	0	1	no prey	no prey	no prey	Night	false.pos
W1881	sway.y	testing.generic	0.1	5	1	1	212	79.4	46.85	4.47	1.45	1	3	10	forage	fish	1	1	chase.cap.hand	successful	benthic	Night	true.pos
W1881	sway.y	testing.generic	0.1	5	2	2	212	79.4	59.35	9.52	3.25	1	3	10	forage	fish	1	1	chase.cap.hand	successful	benthic	Night	true.pos
W1881	sway.y	testing.generic	0.1	5	2	3	212	79.4	65.6	2.84	1.3	1	3	10	forage	fish	0	1	chase.cap.hand	successful	benthic	Night	false.pos
W1881	sway.y	testing.generic	0.1	5	2	4	212	79.4	80.15	4.47	1.45	1	3	10	forage	fish	0	1	chase.cap.hand	successful	benthic	Night	false.pos
W1881	sway.y	testing.generic	0.1	5	2	5	212	79.4	120.6	9.52	3.25	2	3	10	forage	fish	0	1	chase.cap.hand	successful	benthic	Night	false.pos
W1881	sway.y	testing.generic	0.1	5	3	6	212	79.4	132.25	2.84	1.3	1	3	10	forage	fish	1	1	chase(miss)	unsuccessful	benthic	Night	true.pos
W1881	sway.y	testing.generic	0.1	5	3	7	212	79.4	154	4.47	1.45	1	3	10	forage	fish	0	1	chase(miss)	unsuccessful	benthic	Night	false.pos
W1881	sway.y	testing.generic	0.1	5	3	8	212	79.4	159.9	9.52	3.25	1	3	10	forage	fish	0	1	chase(miss)	unsuccessful	benthic	Night	false.pos
W1881	sway.y	testing.generic	0.1	5	3	9	212	79.4	226.3	2.84	1.3	1	3	10	forage	fish	0	1	chase(miss)	unsuccessful	benthic	Night	false.pos
W1881	sway.y	testing.generic	0.1	5	3	10	212	79.4	268.15	4.47	1.45	1	3	10	forage	fish	0	1	chase(miss)	unsuccessful	benthic	Night	false.pos
W1881	sway.y	testing.generic	0.1	5	1	1	213	79.4	87.75	4.34	1.5	1	2	4	forage	fish	1	1	chase.cap.hand	successful	benthic	Night	true.pos
W1881	sway.y	testing.generic	0.1	5	1	2	213	79.4	157.35	22.54	2.55	2	2	4	forage	fish	0	1	chase.cap.hand	successful	benthic	Night	false.pos
W1881	sway.y	testing.generic	0.1	5	2	3	213	79.4	222.1	13.37	4.2	3	2	4	forage	fish	1	1	chase.cap	successful	ascent	Night	true.pos
W1881	sway.y	testing.generic	0.1	5	2	4	213	79.4	234.9	30.77	6.6	3	2	4	forage	fish	0	1	chase.cap	successful	ascent	Night	false.pos
W1881	sway.y	testing.generic	0.1	5	1	1	214	78.9	46.35	6.63	1.55	1	2	14	forage	fish	1	1	chase.cap.hand	successful	benthic	Night	true.pos
W1881	sway.y	testing.generic	0.1	5	1	2	214	78.9	70.6	57.15	4.6	2	2	14	forage	fish	0	1	chase.cap.hand	successful	benthic	Night	false.pos
W1881	sway.y	testing.generic	0.1	5	2	3	214	78.9	87.4	6.39	2.3	1	2	14	forage	fish	1	1	chase(miss)	unsuccessful	benthic	Night	true.pos
W1881	sway.y	testing.generic	0.1	5	2	4	214	78.9	104.55	4.64	1.5	1	2	14	forage	fish	0	1	chase(miss)	unsuccessful	benthic	Night	false.pos
W1881	sway.y	testing.generic	0.1	5	2	5	214	78.9	116.55	6.63	1.55	1	2	14	forage	fish	0	1	chase(miss)	unsuccessful	benthic	Night	false.pos
W1881	sway.y	testing.generic	0.1	5	2	6	214	78.9	143.45	57.15	4.6	1	2	14	forage	fish	0	1	chase(miss)	unsuccessful	benthic	Night	false.pos
W1881	sway.y	testing.generic	0.1	5	2	7	214	78.9	165.4	6.39	2.3	1	2	14	forage	fish	0	1	chase(miss)	unsuccessful	benthic	Night	false.pos
W1881	sway.y	testing.generic	0.1	5	2	8	214	78.9	182.05	4.64	1.5	1	2	14	forage	fish	0	1	chase(miss)	unsuccessful	benthic	Night	false.pos
W1881	sway.y	testing.generic	0.1	5	2	9	214	78.9	188.6	6.63	1.55	1	2	14	forage	fish	0	1	chase(miss)	unsuccessful	benthic	Night	false.pos
W1881	sway.y	testing.generic	0.1	5	2	10	214	78.9	202.1	57.15	4.6	1	2	14	forage	fish	0	1	chase(miss)	unsuccessful	benthic	Night	false.pos
W1881	sway.y	testing.generic	0.1	5	2	11	214	78.9	208.75	6.39	2.3	1	2	14	forage	fish	0	1	chase(miss)	unsuccessful	benthic	Night	false.pos
W1881	sway.y	testing.generic	0.1	5	2	12	214	78.9	227.45	4.64	1.5	1	2	14	forage	fish	0	1	chase(miss)	unsuccessful	benthic	Night	false.pos
W1881	sway.y	testing.generic	0.1	5	2	13	214	78.9	248.7	6.63	1.55	2	2	14	forage	fish	0	1	chase(miss)	unsuccessful	benthic	Night	false.pos
W1881	sway.y	testing.generic	0.1	5	2	14	214	78.9	258.3	57.15	4.6	1	2	14	forage	fish	0	1	chase(miss)	unsuccessful	benthic	Night	false.pos
W1881	sway.y	testing.generic	0.1	5	0	1	215	78.9	42.7	7.31	1.6	1	2	11	forage	no prey	0	1	no prey	no prey	no prey	Night	false.pos
W1881	sway.y	testing.generic	0.1	5	0	2	215	78.9	69.55	2.07	0.85	2	2	11	forage	no prey	0	1	no prey	no prey	no prey	Night	false.pos
W1881	sway.y	testing.generic	0.1	5	1	3	215	78.9	88.35	12.4	3.1	1	2	11	forage	fish	1	1	chase.cap.hand	successful	benthic	Night	true.pos
W1881	sway.y	testing.generic	0.1	5	1	4	215	78.9	99.9	8.78	2.75	1	2	11	forage	fish	0	1	chase.cap.hand	successful	benthic	Night	false.pos
W1881	sway.y	testing.generic	0.1	5	1	5	215	78.9	113.5	7.31	1.6	1	2	11	forage	fish	0	1	chase.cap.hand	successful	benthic	Night	false.pos
W1881	sway.y	testing.generic	0.1	5	1	6	215	78.9	121.65	2.07	0.85	1	2	11	forage	fish	0	1	chase.cap.hand	successful	benthic	Night	false.pos
W1881	sway.y	testing.generic	0.1	5	2	7	215	78.9	157.95	12.4	3.1	1	2	11	forage	fish	1	1	chase.cap	successful	ascent	Night	true.pos
W1881	sway.y	testing.generic	0.1	5	2	8	215	78.9	169.75	8.78	2.75	1	2	11	forage	fish	0	1	chase.cap	successful	ascent	Night	false.pos
W1881	sway.y	testing.generic	0.1	5	2	9	215	78.9	182.4	7.31	1.6	2	2	11	forage	fish	0	1	chase.cap	successful	ascent	Night	false.pos
W1881	sway.y	testing.generic	0.1	5	2	10	215	78.9	209.6	2.07	0.85	1	2	11	forage	fish	0	1	chase.cap	successful	ascent	Night	false.pos
W1881	sway.y	testing.generic	0.1	5	2	11	215	78.9	226.45	12.4	3.1	2	2	11	forage	fish	0	1	chase.cap	successful	ascent	Night	false.pos
W1881	sway.y	testing.generic	0.1	5	1	1	217	79.4	53.25	5.21	1.5	1	2	14	forage	fish	1	1	chase.cap.hand	successful	benthic	Night	true.pos
W1881	sway.y	testing.generic	0.1	5	1	2	217	79.4	70.35	3.71	1.65	1	2	14	forage	fish	0	1	chase.cap.hand	successful	benthic	Night	false.pos
W1881	sway.y	testing.generic	0.1	5	0	3	217	79.4	82.6	3.41	1.25	1	2	14	forage	no prey	0	1	no prey	no prey	no prey	Night	false.pos
W1881	sway.y	testing.generic	0.1	5	0	4	217	79.4	94.9	42.81	4.4	1	2	14	forage	no prey	0	1	no prey	no prey	no prey	Night	false.pos
W1881	sway.y	testing.generic	0.1	5	0	5	217	79.4	102.2	11.26	1.6	1	2	14	forage	no prey	0	1	no prey	no prey	no prey	Night	false.pos
W1881	sway.y	testing.generic	0.1	5	0	6	217	79.4	118.4	5.21	1.5	1	2	14	forage	no prey	0	1	no prey	no prey	no prey	Night	false.pos
W1881	sway.y	testing.generic	0.1	5	0	7	217	79.4	125.95	3.71	1.65	1	2	14	forage	no prey	0	1	no prey	no prey	no prey	Night	false.pos
W1881	sway.y	testing.generic	0.1	5	0	8	217	79.4	137.85	3.41	1.25	2	2	14	forage	no prey	0	1	no prey	no prey	no prey	Night	false.pos
W1881	sway.y	testing.generic	0.1	5	2	9	217	79.4	157.15	42.81	4.4	1	2	14	forage	fish	1	1	chase.cap.hand	successful	benthic	Night	true.pos
W1881	sway.y	testing.generic	0.1	5	2	10	217	79.4	163.45	11.26	1.6	1	2	14	forage	fish	0	1	chase.cap.hand	successful	benthic	Night	false.pos
W1881	sway.y	testing.generic	0.1	5	2	11	217	79.4	169.6	5.21	1.5	2	2	14	forage	fish	0	1	chase.cap.hand	successful	benthic	Night	false.pos
W1881	sway.y	testing.generic	0.1	5	2	12	217	79.4	195.2	3.71	1.65	1	2	14	forage	fish	0	1	chase.cap.hand	successful	benthic	Night	false.pos
W1881	sway.y	testing.generic	0.1	5	2	13	217	79.4	206.65	3.41	1.25	2	2	14	forage	fish	0	1	chase.cap.hand	successful	benthic	Night	false.pos
W1881	sway.y	testing.generic	0.1	5	0	14	217	79.4	225.6	42.81	4.4	1	2	14	forage	no prey	0	1	no prey	no prey	no prey	Night	false.pos
W1881	sway.y	testing.generic	0.1	5	1	1	218	78.9	49.3	9.05	1.55	1	4	10	forage	fish	1	1	chase.cap.hand	successful	benthic	Night	true.pos
W1881	sway.y	testing.generic	0.1	5	1	2	218	78.9	77.55	13.54	2.05	1	4	10	forage	fish	0	1	chase.cap.hand	successful	benthic	Night	false.pos
W1881	sway.y	testing.generic	0.1	5	1	3	218	78.9	108.3	10.15	1.55	1	4	10	forage	fish	0	1	chase.cap.hand	successful	benthic	Night	false.pos
W1881	sway.y	testing.generic	0.1	5	2	4	218	78.9	119.2	50.71	3.7	1	4	10	forage	fish	1	1	chase.cap.hand	successful	benthic	Night	true.pos
W1881	sway.y	testing.generic	0.1	5	2	5	218	78.9	134.05	3.33	1.45	1	4	10	forage	fish	0	1	chase.cap.hand	successful	benthic	Night	false.pos
W1881	sway.y	testing.generic	0.1	5	2	6	218	78.9	152.25	6.34	1.5	1	4	10	forage	fish	0	1	chase.cap.hand	successful	benthic	Night	false.pos
W1881	sway.y	testing.generic	0.1	5	3	7	218	78.9	168.7	44.32	2.9	1	4	10	forage	fish	1	1	chase.cap.hand	successful	benthic	Night	true.pos
W1881	sway.y	testing.generic	0.1	5	3	8	218	78.9	175.15	11.73	1.6	1	4	10	forage	fish	0	1	chase.cap.hand	successful	benthic	Night	false.pos
W1881	sway.y	testing.generic	0.1	5	3	9	218	78.9	187.45	96.61	6.4	1	4	10	forage	fish	0	1	chase.cap.hand	successful	benthic	Night	false.pos
W1881	sway.y	testing.generic	0.1	5	4	10	218	78.9	205.2	22.5	1.85	1	4	10	forage	fish	1	1	chase.cap.hand	successful	ascent	Night	true.pos
W1881	heave.z	testing.generic	0.1	5	1	1	49	67.5	51.4	3.62	1.4	1	3	5	forage	fish	1	1	chase.cap.hand	successful	benthic	Night	true.pos
W1881	heave.z	testing.generic	0.1	5	1	2	49	67.5	59	5.17	2.15	1	3	5	forage	fish	0	1	chase.cap.hand	successful	benthic	Night	false.pos
W1881	heave.z	testing.generic	0.1	5	1	3	49	67.5	68.1	29.8	6.3	2	3	5	forage	fish	0	1	chase.cap.hand	successful	benthic	Night	false.pos
W1881	heave.z	testing.generic	0.1	5	2	4	49	67.5	137.55	3.62	1.4	1	3	5	forage	fish	1	1	chase.cap.hand	successful	benthic	Night	true.pos
W1881	heave.z	testing.generic	0.1	5	3	5	49	67.5	190.75	5.17	2.15	4	3	5	forage	fish	1	1	chase.cap.hand	successful	ascent	Night	true.pos
W1881	heave.z	testing.generic	0.1	5	1	1	50	67.5	194.55	5.18	1.9	2	1	1	forage	fish	1	1	chase.cap.hand	successful	ascent	Night	true.pos
W1881	heave.z	testing.generic	0.1	5	1	1	57	68	66.05	14.41	2.7	1	2	3	forage	fish	1	1	chase.cap.hand	successful	benthic	Night	true.pos
W1881	heave.z	testing.generic	0.1	5	0	2	57	68	82.6	27.95	6	1	2	3	forage	no prey	0	1	no prey	no prey	no prey	Night	false.pos
W1881	heave.z	testing.generic	0.1	5	2	3	57	68	192.2	14.41	2.7	2	2	3	forage	fish	1	1	chase.cap.hand	successful	ascent	Night	true.pos
W1881	heave.z	testing.generic	0.1	5	0	0	81	NA	NA	NA	NA	NA	NA	NA	control	no prey	0	0	no prey	no prey	no prey	Night	true.neg
W1881	heave.z	testing.generic	0.1	5	1	1	82	75.1	87.05	14.35	4.6	3	3	3	forage	fish	1	1	chase.cap.hand	successful	benthic	Night	true.pos
W1881	heave.z	testing.generic	0.1	5	2	2	82	75.1	115.3	11.33	3.05	1	3	3	forage	fish	1	1	cap.hand	successful	benthic	Night	true.pos
W1881	heave.z	testing.generic	0.1	5	3	3	82	75.1	155.05	14.35	4.6	1	3	3	forage	fish	1	1	chase.cap.hand	successful	benthic	Night	true.pos
W1881	heave.z	testing.generic	0.1	5	1	1	83	75.1	79.65	24.81	3.7	1	4	4	forage	fish	1	1	cap.hand	successful	benthic	Night	true.pos
W1881	heave.z	testing.generic	0.1	5	3	2	83	75.1	122.5	30.42	7.85	1	4	4	forage	fish	1	1	chase.cap.hand	successful	benthic	Night	true.pos
W1881	heave.z	testing.generic	0.1	5	3	3	83	75.1	131.85	17.38	1.95	4	4	4	forage	fish	0	1	chase.cap.hand	successful	benthic	Night	false.pos
W1881	heave.z	testing.generic	0.1	5	4	4	83	75.1	162.25	24.81	3.7	1	4	4	forage	unknown	1	1	cap.hand	successful	benthic	Night	true.pos
W1881	heave.z	testing.generic	0.1	5	2	0	83	NA	NA	NA	NA	NA	NA	NA	forage	fish	1	0	chase(miss)	unsuccessful	benthic	Night	false.neg
W1881	heave.z	testing.generic	0.1	5	1	1	107	78	68.35	0.79	0.3	1	2	4	forage	fish	1	1	chase.cap.hand	successful	benthic	Night	true.pos
W1881	heave.z	testing.generic	0.1	5	1	2	107	78	73.75	10.72	3.1	2	2	4	forage	fish	0	1	chase.cap.hand	successful	benthic	Night	false.pos
W1881	heave.z	testing.generic	0.1	5	1	3	107	78	83.45	6.2	1.7	3	2	4	forage	fish	0	1	chase.cap.hand	successful	benthic	Night	false.pos
W1881	heave.z	testing.generic	0.1	5	2	4	107	78	123.2	3.75	1.65	2	2	4	forage	fish	1	1	chase.cap.hand	successful	benthic	Night	true.pos
W1881	heave.z	testing.generic	0.1	5	1	1	144	79.6	85.45	3.46	1.4	2	3	5	forage	unknown	1	1	chase.cap.hand	successful	benthic	Day	true.pos
W1881	heave.z	testing.generic	0.1	5	1	2	144	79.6	91.9	5.36	1.5	1	3	5	forage	unknown	0	1	chase.cap.hand	successful	benthic	Day	false.pos
W1881	heave.z	testing.generic	0.1	5	1	3	144	79.6	102.9	3.46	1.4	1	3	5	forage	unknown	0	1	chase.cap.hand	successful	benthic	Day	false.pos
W1881	heave.z	testing.generic	0.1	5	0	4	144	79.6	114.25	5.36	1.5	1	3	5	forage	no prey	0	1	no prey	no prey	no prey	Day	false.pos
W1881	heave.z	testing.generic	0.1	5	3	5	144	79.6	170.2	3.46	1.4	1	3	5	forage	unknown	1	1	chase(miss)	unsuccessful	benthic	Day	true.pos
W1881	heave.z	testing.generic	0.1	5	2	0	144	NA	NA	NA	NA	NA	NA	NA	forage	unknown	1	0	cap.hand	successful	benthic	Day	false.neg
W1881	heave.z	testing.generic	0.1	5	1	1	145	79.6	70.5	3.02	1.3	1	3	6	forage	fish	1	1	cap.hand	successful	benthic	Day	true.pos
W1881	heave.z	testing.generic	0.1	5	2	2	145	79.6	87.35	8.71	2.8	1	3	6	forage	unknown	1	1	cap.hand	successful	benthic	Day	true.pos
W1881	heave.z	testing.generic	0.1	5	0	3	145	79.6	100.9	3.47	1.4	1	3	6	forage	no prey	0	1	no prey	no prey	no prey	Day	false.pos
W1881	heave.z	testing.generic	0.1	5	0	4	145	79.6	112.8	3.02	1.3	1	3	6	forage	no prey	0	1	no prey	no prey	no prey	Day	false.pos
W1881	heave.z	testing.generic	0.1	5	3	5	145	79.6	148.55	8.71	2.8	2	3	6	forage	unknown	1	1	chase(miss)	unsuccessful	benthic	Day	true.pos
W1881	heave.z	testing.generic	0.1	5	3	6	145	79.6	162.35	3.47	1.4	1	3	6	forage	unknown	0	1	chase(miss)	unsuccessful	benthic	Day	false.pos
W1881	heave.z	testing.generic	0.1	5	1	1	146	79.6	52.3	19.1	3.6	1	4	7	forage	unknown	1	1	cap.hand	successful	benthic	Day	true.pos

W1881	heave.z	testing.generic	0.1	5	2	2	146	79.6	62.15	14.68	4.2	1	4	7	forage	fish	1	1	cap.hand	successful	benthic	Day	true.pos
W1881	heave.z	testing.generic	0.1	5	2	3	146	79.6	71.7	2.63	1.2	1	4	7	forage	fish	0	1	cap.hand	successful	benthic	Day	false.pos
W1881	heave.z	testing.generic	0.1	5	3	4	146	79.6	85.75	19.1	3.6	3	4	7	forage	fish	1	1	chase.cap.hand	successful	benthic	Day	true.pos
W1881	heave.z	testing.generic	0.1	5	4	5	146	79.6	142.8	14.68	4.2	1	4	7	forage	unknown	1	1	chase.cap.hand	successful	ascent	Day	true.pos
W1881	heave.z	testing.generic	0.1	5	4	6	146	79.6	155	2.63	1.2	1	4	7	forage	unknown	0	1	chase.cap.hand	successful	ascent	Day	false.pos
W1881	heave.z	testing.generic	0.1	5	4	7	146	79.6	161.35	19.1	3.6	1	4	7	forage	unknown	0	1	chase.cap.hand	successful	ascent	Day	false.pos
W1881	heave.z	testing.generic	0.1	5	0	1	147	79.6	68.35	8.41	1.55	1	2	6	forage	no prey	0	1	no prey	no prey	no prey	Night	false.pos
W1881	heave.z	testing.generic	0.1	5	0	2	147	79.6	96.1	6.48	1.5	1	2	6	forage	no prey	0	1	no prey	no prey	no prey	Night	false.pos
W1881	heave.z	testing.generic	0.1	5	1	3	147	79.6	114.4	9.79	1.6	1	2	6	forage	unknown	1	1	cap.hand	successful	benthic	Night	true.pos
W1881	heave.z	testing.generic	0.1	5	2	4	147	79.6	144.75	2.74	1.25	1	2	6	forage	unknown	1	1	cap.hand	successful	benthic	Night	true.pos
W1881	heave.z	testing.generic	0.1	5	0	5	147	79.6	159.8	6.04	1.5	1	2	6	forage	no prey	0	1	no prey	no prey	no prey	Night	false.pos
W1881	heave.z	testing.generic	0.1	5	0	6	147	79.6	186.2	3.17	1.3	1	2	6	forage	no prey	0	1	no prey	no prey	no prey	Night	false.pos
W1881	heave.z	testing.generic	0.1	5	1	1	148	79.6	66.95	4.32	1.65	2	4	9	forage	unknown	1	1	cap.hand	successful	benthic	Night	true.pos
W1881	heave.z	testing.generic	0.1	5	2	2	148	79.6	79.25	47.66	7.15	4	4	9	forage	fish	1	1	cap.hand	successful	benthic	Night	true.pos
W1881	heave.z	testing.generic	0.1	5	0	3	148	79.6	99.65	8.33	2.7	1	4	9	forage	no prey	0	1	no prey	no prey	no prey	Night	false.pos
W1881	heave.z	testing.generic	0.1	5	0	4	148	79.6	105.9	5.28	1.5	1	4	9	forage	no prey	0	1	no prey	no prey	no prey	Night	false.pos
W1881	heave.z	testing.generic	0.1	5	0	5	148	79.6	116.15	4.32	1.65	1	4	9	forage	no prey	0	1	no prey	no prey	no prey	Night	false.pos
W1881	heave.z	testing.generic	0.1	5	3	6	148	79.6	127.4	47.66	7.15	1	4	9	forage	unknown	1	1	cap.hand	successful	benthic	Night	true.pos
W1881	heave.z	testing.generic	0.1	5	4	7	148	79.6	146.25	8.33	2.7	2	4	9	forage	unknown	1	1	cap.hand	successful	benthic	Night	true.pos
W1881	heave.z	testing.generic	0.1	5	4	8	148	79.6	154.5	5.28	1.5	1	4	9	forage	unknown	0	1	cap.hand	successful	benthic	Night	false.pos
W1881	heave.z	testing.generic	0.1	5	0	9	148	79.6	184.7	4.32	1.65	1	4	9	forage	no prey	0	1	no prey	no prey	no prey	Night	false.pos
W1881	heave.z	testing.generic	0.1	5	2	1	212	79.4	58.55	27.93	3.4	1	3	5	forage	fish	1	1	chase.cap.hand	successful	benthic	Night	true.pos
W1881	heave.z	testing.generic	0.1	5	2	2	212	79.4	65.6	3.77	1.5	1	3	5	forage	fish	0	1	chase.cap.hand	successful	benthic	Night	false.pos
W1881	heave.z	testing.generic	0.1	5	3	3	212	79.4	132.55	29.95	3.55	1	3	5	forage	fish	1	1	chase(miss)	unsuccessful	benthic	Night	true.pos
W1881	heave.z	testing.generic	0.1	5	3	4	212	79.4	153.1	2.81	1.25	1	3	5	forage	fish	0	1	chase(miss)	unsuccessful	benthic	Night	false.pos
W1881	heave.z	testing.generic	0.1	5	3	5	212	79.4	159.75	15.36	1.75	1	3	5	forage	fish	0	1	chase(miss)	unsuccessful	benthic	Night	false.pos
W1881	heave.z	testing.generic	0.1	5	1	0	212	NA	NA	NA	NA	NA	NA	NA	forage	fish	1	0	chase.cap.hand	successful	benthic	Night	false.neg
W1881	heave.z	testing.generic	0.1	5	1	1	213	79.4	157.25	7.98	1.55	1	2	2	forage	fish	1	1	chase.cap.hand	successful	benthic	Night	true.pos
W1881	heave.z	testing.generic	0.1	5	2	2	213	79.4	234.85	28.14	5.1	3	2	2	forage	fish	1	1	chase.cap	successful	ascent	Night	true.pos
W1881	heave.z	testing.generic	0.1	5	1	1	214	78.9	70.55	30.44	3.05	1	2	3	forage	fish	1	1	chase.cap.hand	successful	benthic	Night	true.pos
W1881	heave.z	testing.generic	0.1	5	2	2	214	78.9	181.95	0.84	0.35	1	2	3	forage	fish	1	1	chase(miss)	unsuccessful	benthic	Night	true.pos
W1881	heave.z	testing.generic	0.1	5	2	3	214	78.9	227.45	6.8	1.5	1	2	3	forage	fish	0	1	chase(miss)	unsuccessful	benthic	Night	false.pos
W1881	heave.z	testing.generic	0.1	5	2	1	215	78.9	169.4	5.74	1.5	1	2	4	forage	fish	1	1	chase.cap	successful	ascent	Night	true.pos
W1881	heave.z	testing.generic	0.1	5	2	2	215	78.9	182.45	16.28	3.1	1	2	4	forage	fish	0	1	chase.cap	successful	ascent	Night	false.pos
W1881	heave.z	testing.generic	0.1	5	2	3	215	78.9	209.8	5.74	1.5	1	2	4	forage	fish	0	1	chase.cap	successful	ascent	Night	false.pos
W1881	heave.z	testing.generic	0.1	5	2	4	215	78.9	226.45	16.28	3.1	2	2	4	forage	fish	0	1	chase.cap	successful	ascent	Night	false.pos
W1881	heave.z	testing.generic	0.1	5	1	0	215	NA	NA	NA	NA	NA	NA	NA	forage	fish	1	0	chase.cap.hand	successful	benthic	Night	false.neg
W1881	heave.z	testing.generic	0.1	5	1	1	217	79.4	70.2	4.93	1.6	1	2	9	forage	fish	1	1	chase.cap.hand	successful	benthic	Night	true.pos
W1881	heave.z	testing.generic	0.1	5	0	2	217	79.4	82.1	2.69	1.2	1	2	9	forage	no prey	0	1	no prey	no prey	no prey	Night	false.pos
W1881	heave.z	testing.generic	0.1	5	0	3	217	79.4	95.25	4.02	1.4	1	2	9	forage	no prey	0	1	no prey	no prey	no prey	Night	false.pos
W1881	heave.z	testing.generic	0.1	5	0	4	217	79.4	101.95	8.3	1.65	1	2	9	forage	no prey	0	1	no prey	no prey	no prey	Night	false.pos
W1881	heave.z	testing.generic	0.1	5	0	5	217	79.4	125.85	6.29	1.5	1	2	9	forage	no prey	0	1	no prey	no prey	no prey	Night	false.pos
W1881	heave.z	testing.generic	0.1	5	2	6	217	79.4	157	11.2	1.6	1	2	9	forage	fish	1	1	chase.cap.hand	successful	benthic	Night	true.pos
W1881	heave.z	testing.generic	0.1	5	2	7	217	79.4	163.6	9.5	1.5	1	2	9	forage	fish	0	1	chase.cap.hand	successful	benthic	Night	false.pos
W1881	heave.z	testing.generic	0.1	5	2	8	217	79.4	209.85	12.74	2.2	1	2	9	forage	fish	0	1	chase.cap.hand	successful	benthic	Night	false.pos
W1881	heave.z	testing.generic	0.1	5	0	9	217	79.4	225.65	2.99	1.35	1	2	9	forage	no prey	0	1	no prey	no prey	no prey	Night	false.pos
W1881	heave.z	testing.generic	0.1	5	1	1	218	78.9	48.95	2.24	1.1	1	4	8	forage	fish	1	1	chase.cap.hand	successful	benthic	Night	true.pos
W1881	heave.z	testing.generic	0.1	5	1	2	218	78.9	77.9	12.47	3.35	1	4	8	forage	fish	0	1	chase.cap.hand	successful	benthic	Night	false.pos
W1881	heave.z	testing.generic	0.1	5	1	3	218	78.9	108.1	39.33	6	1	4	8	forage	fish	0	1	chase.cap.hand	successful	benthic	Night	false.pos
W1881	heave.z	testing.generic	0.1	5	2	4	218	78.9	118.1	13.36	1.8	2	4	8	forage	fish	1	1	chase.cap.hand	successful	benthic	Night	true.pos
W1881	heave.z	testing.generic	0.1	5	2	5	218	78.9	152.6	2.24	1.1	1	4	8	forage	fish	0	1	chase.cap.hand	successful	benthic	Night	false.pos
W1881	heave.z	testing.generic	0.1	5	3	6	218	78.9	168.55	12.47	3.35	1	4	8	forage	fish	1	1	chase.cap.hand	successful	benthic	Night	true.pos
W1881	heave.z	testing.generic	0.1	5	3	7	218	78.9	183.75	39.33	6	2	4	8	forage	fish	0	1	chase.cap.hand	successful	benthic	Night	false.pos
W1881	heave.z	testing.generic	0.1	5	4	8	218	78.9	205.25	13.36	1.8	1	4	8	forage	fish	1	1	chase.cap.hand	successful	ascent	Night	true.pos
