# Supplementary material for: Identification of Prey Captures in Australian Fur Seals (Arctocephalus pusillus doriferus) Using Head-Mounted Accelerometers: Field Validation with Animal-Borne Video Cameras
Source: PLoS One. 2015 Jun 24;10(6):e0128789. doi: 10.1371/journal.pone.0128789 (PMC4479472; doi:10.1371/journal.pone.0128789)
Supplement: S1 Text — (DOCX) [file pone.0128789.s003.docx]

**Supporting Information S1. Description of Function 1 that identified attempted prey captures (APC) and details on how video was matched to accelerometer data.** Identification of APC from the accelerometry data was performed using a custom-written function in R modified from Viviant et al. (2010) to include integration of the area under the peak of variance, differences in species foraging behaviour and variation among a greater number of animals (referred to as Function 1, Fig. 2, available on request from authors). Function 1 was comprised of a suite of several custom-written functions and routines nested within each other in R. Each peak in variance of acceleration that crossed the variance threshold value was counted as an individual APC (Fig. 2 D). If two such consecutive peaks were greater than the minimum interval threshold apart, then the peaks were counted as separate APCs (2 separate prey, Fig. 2 C). If two consecutive peaks were separated by less than the minimum duration threshold, then the peaks were grouped into a single APC. We interpreted this as multiple head movements during an attempt to capture the same prey or potential prey handling. This assumption was verified independently from video for individual APCs on a random subsample of dives from all animals (approximately 10% of training dives). The exact time of the APC was considered to be at the apex of the first peak of the accelerometer data (measured at 20 Hz). The actual consumption of prey theoretically occurred a fraction of a second to a couple of seconds after the head movement was detected by the accelerometer. However, the detection values were the same or lower from post hoc testing of using the last peak or halfway point peak. We also calculated the integral of the variance signal (the cumulative area below the variance peak, g^2^) for each prey capture, as a measure of the cumulative amplitude or strength of each APC. If there were multiple peaks in an APC, the sum of all integrals was calculated to provide a cumulative integral of that event. The start and end point of the integral was matched to a corresponding time vector, which also provided a measure of the duration of each APC. The duration of the APC estimated from the integral duration was an underestimate of the actual APC duration because the integral duration only included the durations of peaks occurring above the variance threshold.

Additional data processing was required to remove artifacts in clock alignment among the TDR, video camera, and accelerometer. The time stamps of the TDR, accelerometer, and video data were aligned visually to within ± 1 s using the raw surge acceleration with Eonfusion software (Eonfusion, v.1.2, www.myriax.com**)** and customized functions in R for each individual dive before running Function 1. The APC identified by the accelerometer were matched to events identified on video data using custom matching functions and nested loops in R. Accuracy of automating this process was verified by plotting Figure 2 with the video APC times overlaid for each individual dive on the surge axis at 0.1 variance threshold and 5 s minimum interval parameters. All dives in both the training and testing subset were plotted and verified that matching was accurate. First, the video windows for each specific APC (start of chase to end of handling if successful or end of chase if unsuccessful) were matched with each dive from the TDR and the corresponding variance of acceleration. Next, the matching function searched within a spreadsheet database of the enumerated video clips for that specific dive to find the specific APC on video that included the estimated time of the APC on the accelerometer. Dataloggers were aligned to within ± 1 s (see above), but to further mitigate this a ± 5 s buffer was added to either side of the video window.
